# Community Survey Results Show that Standardisation of Preclinical Imaging Techniques Remains a Challenge

**DOI:** 10.1007/s11307-022-01790-6

**Published:** 2022-12-08

**Authors:** Adriana A. S. Tavares, Laura Mezzanotte, Wendy McDougald, Monique R. Bernsen, Christian Vanhove, Markus Aswendt, Giovanna D. Ielacqua, Felix Gremse, Carmel M. Moran, Geoff Warnock, Claudia Kuntner, Marc C. Huisman

**Affiliations:** 1 BHF-University Centre for Cardiovascular Science, University of Edinburgh, Edinburgh, UK; 2 Edinburgh Imaging, University of Edinburgh, Edinburgh, UK; 3 Department of Radiology and Nuclear Medicine, Erasmus MC University Medical Center Rotterdam, Rotterdam, The Netherlands; 4 Siemens, Molecular Imaging, Hoffman Estates, IL USA; 5 AMIE Core Facility, Erasmus MC University Medical Center Rotterdam, Rotterdam, The Netherlands; 6 Faculty of Engineering and Architecture, Department of Electronics and Information Systems, Ghent University, Ghent, Belgium; 7 Faculty of Medicine, Dept. of Neurology, University of Cologne, and University Hospital Cologne, Cologne, Germany; 8 Max-Delbrück Center for Molecular Medicine, in the Helmholtz Association, Berlin, Germany; 9 Gremse-IT GmbH, Aachen, Germany; 10 Experimental Molecular Imaging, RWTH Aachen University Clinic, Aachen, Germany; 11 University of Zurich, Zurich, Switzerland; 12 Department of Biomedical Imaging and Image-Guided Therapy, Medical University of Vienna, Vienna, Austria; 13 Department of Radiology and Nuclear Medicine, Amsterdam UMC, Vrije Universiteit Amsterdam, Amsterdam, The Netherlands; 14 Cancer Center Amsterdam, Amsterdam, The Netherlands

**Keywords:** Standardisation, Preclinical imaging, Community, Optical, MRI, CT, PET, SPECT, Ultrasound

## Abstract

**Purpose:**

To support acquisition of accurate, reproducible and high-quality preclinical imaging data, various standardisation resources have been developed over the years. However, it is unclear the impact of those efforts in current preclinical imaging practices. To better understand the *status quo* in the field of preclinical imaging standardisation, the STANDARD group of the European Society of Molecular Imaging (ESMI) put together a community survey and a forum for discussion at the European Molecular Imaging Meeting (EMIM) 2022. This paper reports on the results from the STANDARD survey and the forum discussions that took place at EMIM2022.

**Procedures:**

The survey was delivered to the community by the ESMI office and was promoted through the Society channels, email lists and webpages. The survey contained seven sections organised as generic questions and imaging modality-specific questions. The generic questions focused on issues regarding data acquisition, data processing, data storage, publishing and community awareness of international guidelines for animal research. Specific questions on practices in optical imaging, PET, CT, SPECT, MRI and ultrasound were further included.

**Results:**

Data from the STANDARD survey showed that 47% of survey participants do not have or do not know if they have QC/QA guidelines at their institutes. Additionally, a large variability exists in the ways data are acquired, processed and reported regarding general aspects as well as modality-specific aspects. Moreover, there is limited awareness of the existence of international guidelines on preclinical (imaging) research practices.

**Conclusions:**

Standardisation of preclinical imaging techniques remains a challenge and hinders the transformative potential of preclinical imaging to augment biomedical research pipelines by serving as an easy vehicle for translation of research findings to the clinic. Data collected in this project show that there is a need to promote and disseminate already available tools to standardise preclinical imaging practices.

**Supplementary Information:**

The online version contains supplementary material available at 10.1007/s11307-022-01790-6.

## Introduction

Major advances in instrumentation and technology over the past decades have enabled the development and rapid expansion of a whole new field in medical imaging; that is the preclinical imaging field. Research activities in this new field using all major modalities, namely, positron emission tomography (PET), computed tomography (CT), magnetic resonance imaging (MRI), single photon emission computed tomography (SPECT), ultrasound and optical imaging; have flourished and continue to expand [1–3]. Preclinical imaging techniques have been used to observe and understand pathophysiological processes in animal models of human disease [4, 5] as well as to test new procedures, methods and probes [6, 7]. They have been instrumental to pharmaceutical companies looking to discover and develop new therapeutic interventions [8]. Unfortunately, the generalised acceptance that preclinical imaging does not aim to provide diagnostic information, unlike clinical imaging, and rather aims at methodological developments, has hindered efforts to deliver high quality, reproducible, reliable and translatable information from preclinical imaging studies.

Albeit not focused on directly producing diagnostic information, preclinical imaging uses animal models to understand a disease's underlying biology and pathology. According to the latest European Union (EU) statistics, a large number of animals (10.4 million in 2019 alone in the EU) are used for medical research. With the rapid advent of preclinical imaging [4], many of these would be part of research projects making use of preclinical imaging techniques, which in turn has a proven reduction effect on animal numbers due to repetitive imaging and less need of multiple subgroups to cover a disease development timeline [9]. Importantly, the ethical rule of the 3Rs (reduction, refinement and replacement) exists since 1959 [10] and all reputable funding bodies and scientific journals require adherence to said ethical principles. Yet, it has been recognised that, more often than not, preclinical imaging standards fall shorter than those expected and used in the clinical setting [11, 12]. This is an important problem that needs to be understood and addressed by the preclinical imaging community. To that end, the STANDARD group of the European Society for Molecular Imaging (ESMI) has put together a survey directed at the preclinical imaging community with three main aims: (1) to gather knowledge on the current state-of-the-art of preclinical imaging quality control (QC), quality assurance (QA) and standardisation procedures routinely used at different sites; (2) to evoke discussion on current status of preclinical imaging standardisation and what is needed to increase impact of preclinical imaging findings in the translational pipeline towards the clinic; and (3) to initiate a consensus community-led paper on best practice when collecting, analysing and publishing preclinical imaging data.

This paper will present the results from the STANDARD survey and the key outcomes from the community discussions that took place during the STANDARD session of EMIM 2022. Furthermore, here we provide structured guidance on already available resources to support best-practice collection, analysis and publication of preclinical imaging data.

## Materials and Methods

### Survey Structure and Questions

The STANDARD community survey contained seven sections organised as generic questions and imaging modality-specific questions.

The generic questions were focussed on understanding current practices regarding data acquisition, processing and management in the field of preclinical imaging. They also aimed to assess community awareness on key available international guidelines for animal research (i.e. ARRIVE guidelines [13, 14]), radionuclide imaging (i.e. AQARA guidelines [15]) and data sharing practices (i.e. FAIR guidelines [16]). Finally, the generic questions of the questionnaire aimed to understand *status quo* in publishing and sharing preclinical imaging data, as well as capturing the community views on how important accreditation practices are for the preclinical imaging field.

Six different preclinical imaging modalities were included in the STANDARD survey. These were optical imaging, PET, CT, SPECT, MRI and ultrasound. Specific questions per modality were organised into six sections of the survey.

### Survey Participants

A total of 151 colleagues working in the field of preclinical imaging participated in the STANDARD survey. The majority of those participating in the survey were principal investigators or group leaders (50%) followed by post-doctoral scientists (28%), PhD students (10%), technicians (8%) and service engineers (4%). Geographical distribution of survey participants varied across three continents (Europe, America and Asia) and included participants from Germany (25%); the UK and Belgium (11% per country); the Netherlands (10%); Spain and Italy (7% per country); France and the USA (6% per country); Switzerland and Austria (3% per country); Denmark, Israel and Norway (2% per country); and China, Croatia, Czech Republic, Greece, Japan, Poland and Ukraine (< 1% per country).

Survey participants were required to answer the generic questions, but could skip the modality-specific questions if that was not applicable to them.

### Survey Data Collection and Analysis

The survey was delivered to the community by the ESMI office and was promoted through the Society channels, email lists and webpages. The STANDARD group also promoted this survey via social media and professional pages or mailing lists. SurveyMonkey (Momentive, USA) was used as the platform to post questions and collect results. The survey started on the 10^th^ December 2021 and ended on the 22^nd^ January 2022. Data was extracted from SurveyMonkey as Excel and PDF extracts. Graph plotting summarising all data collected was conducted using Prism 9.3 (GraphPad Software, USA).

### EMIM 2022 STANDARD Session

On the 15^th^ March 2022, during the EMIM 2022 in Thessaloniki, Greece, the STANDARD study group session was structured as follow: (1) overview of survey aims and presentation of results from the generic questions; (2) presentation of results from each modality-specific questions; and (3) roundtable discussion to gather community feedback on results presented and future directions. Outcomes of this roundtable discussion was collated and will be discussed in this paper.

## Results

All responses to all questions in the STANDARD survey are presented in the Supplementary File 1. Personal data and contact information from survey participants were removed from Supplementary File 1 to comply with data protection requirements. A summary of the key findings is provided in the results sections below.

### Outcome from Generic Survey Questions

**Fig. 1.**
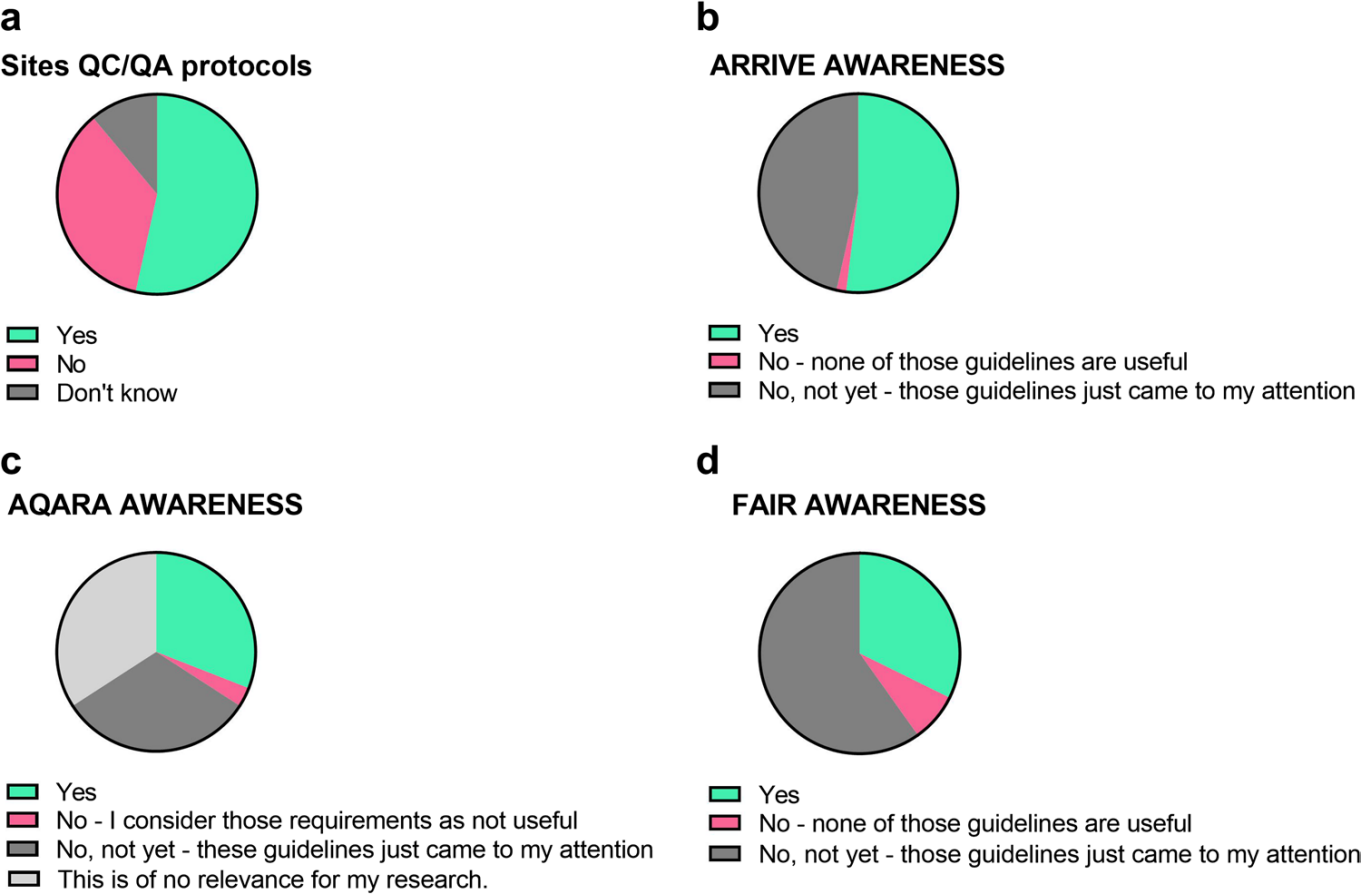
Many preclinical imaging sites still need to implement QC/QA protocols **(a)** and many preclinical imaging researchers are not aware of the ARRIVE **(b)**, AQARA **(c)** and FAIR **(d)** guidelines.

Data from the STANDARD survey showed that 47% of survey participants don’t have or don’t know if they have QC/QA guidelines at their institutes (Fig. 1a). However, among those who have QC/QA guidelines at their institutes, the vast majority (69%) keeps records of QC/QA performance of scanners, maintenance, and system failures. When asked about the importance of preclinical imaging standardisation/accreditation and reporting QC/QA results when publishing preclinical imaging data, survey participants rated this neither essential nor necessary with average score of 2 and 2.7 out of 5, respectively.

With regards to the use of standard operating procedures (SOPs), 68%, 52% and 40% of participants said they used SOPs for acquisition, reconstruction and analysis respectively. A total of 30% of survey participants state they did not use SOPs for any parts of their preclinical imaging studies. When publishing their preclinical imaging data, the majority of the survey participants report acquisition parameters (96%), image analysis methods (92%) and reconstruction parameters (76%). Only a very small percentage (0.8%) reports on none of these parameters/methods.

Various approaches are used for handling and storing preclinical imaging data. The most common data formats, based on survey results, were DICOM (64%), scanner vendor specific format (60%), NIfTI (36%), BIDS (4%) and others (15%), including jpeg and tiff files. Most survey participants store their preclinical imaging data in central network storage facilities (73%) followed by NAS (15%), PACS (10%) or XNAT (5%). The remaining survey participants do not use central storage and instead rely on local hard drive storage. Most survey participants stated they archive their preclinical imaging data (79%), albeit 18% only do that using local hard drives, with only a small minority not archiving imaging data (3%).

The top three most used software packages for preclinical image analysis were ImageJ, Matlab and PMOD; followed by vendor specific software packages; and vivoQuant. AMIDE, Python, FSL and SPM are also popular software packages among preclinical image users.

A total of 46% of survey participants were not aware of the ARRIVE guidelines and 2% did not think said guidelines are useful (Fig. 1b). Among those working with nuclear medicine techniques (PET and SPECT), half was not aware of the AQARA guidelines and a small minority did not think that the AQARA guidelines are useful (Fig. 1c). The vast majority of survey participants was not aware of the FAIR guidelines (Fig. 1d).

### Optical Imaging

Nearly 35% of the survey participants declared use of optical imaging instrumentation both for bioluminescence and fluorescence acquisition, with 53% of those indicating that the instruments used at their centres undergo maintenance and are calibrated once a year on average. When reporting quantification of signals from acquired images, 74% of the participants use photon fluxes, although 84% of the participants also consider reporting relative differences between photon fluxes or average radiance as an acceptable method for semi-quantitate analysis of optical imaging datasets. Furthermore, 81% of the survey participants also consider it acceptable to use a luminescent standard as a reference for quantitative analysis of optical imaging datasets. Importantly, it should be noted here that photon fluxes and/or counts are strictly dependent on the light detector and the geometry of the instrumentation; therefore, comparison of datasets collected using different imaging instrumentation remain unachievable without the use of standards. Around 90% of participants declare that they are in favour of the adoption of luminescent standards and/or phantoms (preferentially with a price range of €500–1000) as a mean to standardize optical imaging protocols, compare performances of instrumentations and compare datasets.

Some survey questions were designed to inform the development of guidelines for reporting optical imaging experiments in scientific journals. Results from the survey showed that the community ranked highly the need to report the instrument used, the time of acquisition, filters, fields of view, camera aperture, dose of substrates, as well as description of the reconstruction parameters used for 3D analysis. This suggests that the imaging community, represented by the survey participants, felt that without proper description of imaging procedures in scientific articles, research methods and outputs are not reproducible.

### PET Imaging

**Fig. 2.**
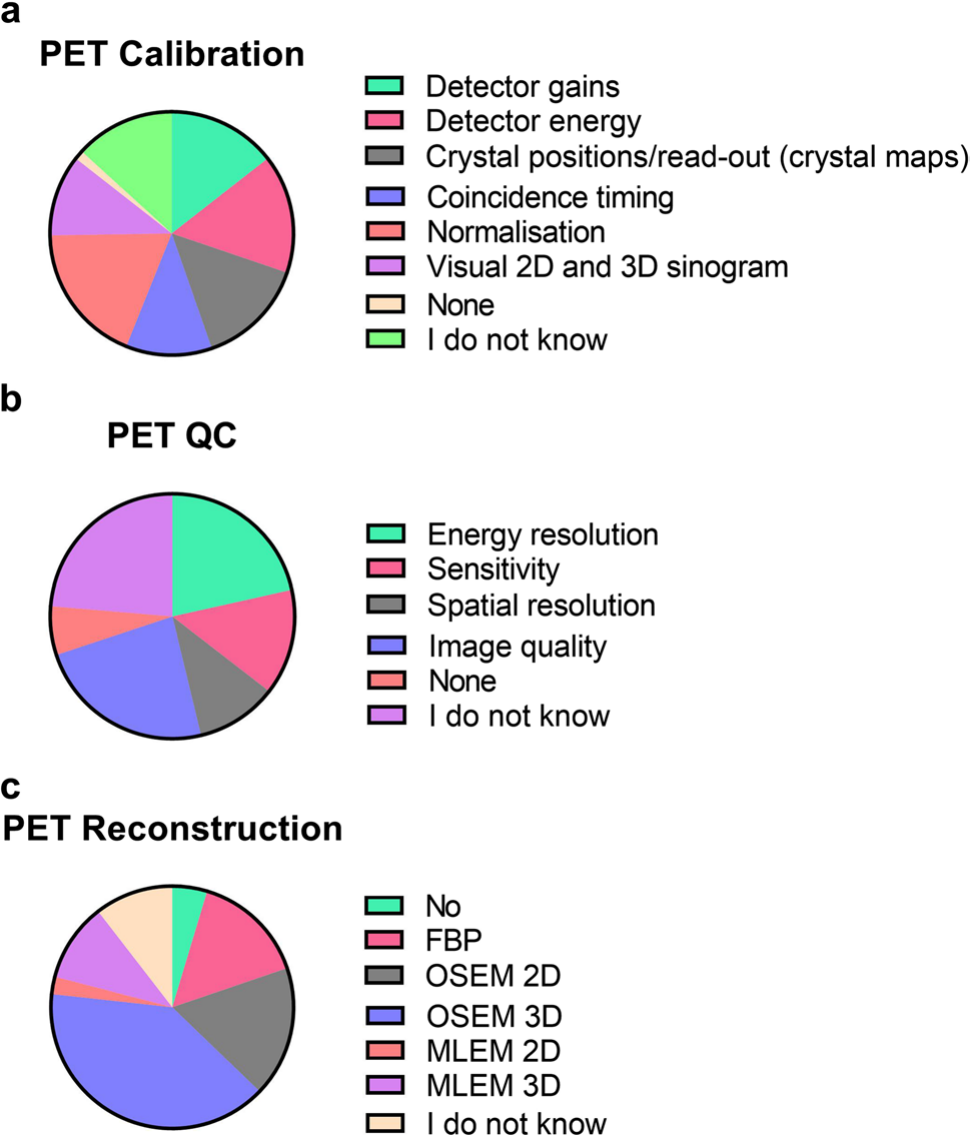
PET survey participants’ responses when asked about scanner calibration protocol **(a)**, typical QC protocol **(b)** and routine reconstruction methods **(c)** used at their different imaging facilities.

Nearly 50% of the survey participants indicated they use PET. Of those 77% carry out scanner calibrations (quarterly, 26%; biannually, 23% or annually, 28%). However, 3% did not perform regular scanner calibration and 19% responded that they did not know if regular scanner calibration was performed (Fig. 2a). Moreover, the majority of users regularly perform scanner QC (34% daily, 20% weekly and 21% monthly). Yet, 5% report performing no QC and 20% reported that they did not know (Fig. 2b).

Additionally, the majority of participants (57%) perform cross-calibration between dose calibrator, PET scanner and/or gamma counter, but 16% responded that cross-calibration did not occur and 27% responded that they did not know if it occurred. On the days images are acquired, 62% of participants indicated performing a visual inspection (artefacts), 47% a detector check and 27% performed a co-registration check with CT (if applicable). A total of 7% of participants responded performing none and 24% did not know (Fig. 2c).

### SPECT Imaging

Survey results showed that 20% of survey participants are working with preclinical SPECT devices. Most users (88%) are using preclinical SPECT for in vivo imaging experiments, whereby the majority (85%) of the experiments involves quantitative imaging. Ex vivo quantitative SPECT imaging experiments are performed by 31% of the survey participants. To check quantitative accuracy, cross-calibration between dose calibrator and SPECT scanner is performed by 65% of the participants, most frequently every quarter (31%). Cross-calibration also includes SPECT scanner versus a gamma counter in 46% of the SPECT users.

Only 36%, 40%, 44% and 48% of the survey participants uses QC procedures such as photopeak drift, uniformity testing, collimator checking and multimodal registration, respectively. Nonetheless, yearly (manufacturer) maintenance of the SPECT scanner is being performed by 73% of the participants and even more than once a year by 31% of the participants. Only 4% of SPECT users does not perform regular maintenance on their SPECT system.

The most common methods for image reconstruction, based on survey results, were OSEM 3D (36%), and MLEM 3D (20%) followed by OSEM 2D (12%) FBP (12%) and MLEM 2D (4%). Furthermore, 40% of the SPECT users did not know which reconstruction algorithm was being used.

### MRI Imaging

**Fig. 3.**
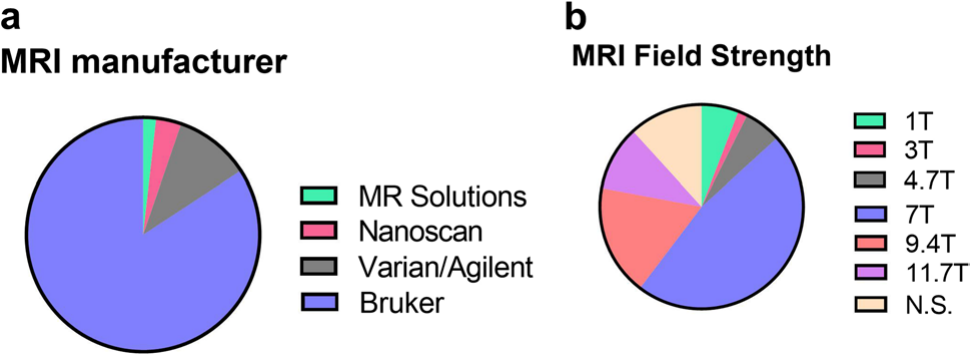
MRI survey participants’ responses when asked about MRI manufacturer **(a)** and MRI field strength **(b)** used at their different imaging facilities.

Survey results regarding the MRI part of the questionnaire showed that over half (52%) of the participants are working with preclinical MRI devices. The vast majority of the users have Bruker manufactured scanners (84%), followed by Varian/Agilent devices (10%), Nanoscan (3%) and MR Solutions (2%) (Fig. 3a). Field strengths of the MR scanners range from a minimum of 1 Tesla (6%) to a maximum of 11.7 T devices (10% of the users), although most of the scanners used are either 7 T, 47% or 9.4 T, 18%, with much smaller percentages of participants using 4.7 T and 3 T devices (6% and 2% respectively) (Fig. 3b). The most common scans acquired are T2 (23%) and T1 (23%) followed by diffusion-weighted imaging (DWI) (15%), perfusion (14%) and functional MRI (fMRI) (17%).

**Fig. 4.**
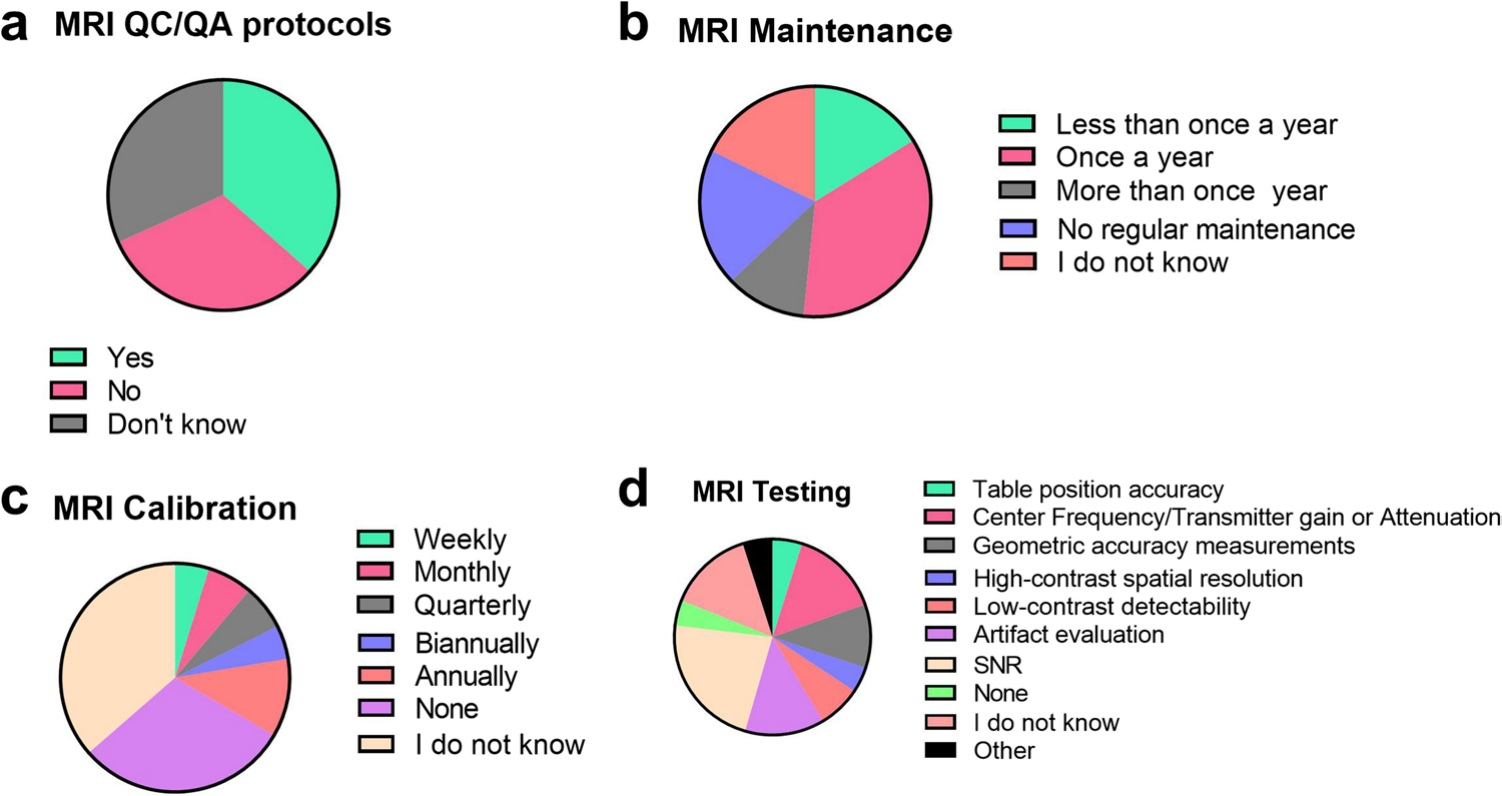
MRI survey participants’ responses when asked about MRI QC/QA protocols **(a)**, maintenance **(b)**, calibration **(c)** and testing **(d)** at their different imaging facilities.

When asked if survey participants follow any QA procedures, 63% of the respondents either do not follow any procedure (32%) or do not know/never heard of it (32%) (Fig. 4a). The yearly frequency of scanner-maintenance performed by the manufacturer is in most of the laboratories once a year (35%), but a fairly big percentage (19%) of users declare that no regular maintenance is performed while 11% do it more than once a year. Furthermore, 18% of the survey participants responding that they do not know the frequency of regular scanner maintenance visits by the scanner manufacturer (Fig. 4b).

Regarding scanner calibration, over two-thirds (67%) of the users do not know the calibration frequency (36%) or declare to not perform regular calibration (30%) while other participants perform it annually (11%), biannually (5%), quarterly (6%), monthly (6%) or weekly (5%) (Fig. 4c).

Quality control is performed with a wide range of phantoms: manufacturer-supplied ones, water-based, agar-based, homemade are the most commonly used. Among regular QC testing, there are signal-to-noise ratio (SNR) measurements (53%), artefacts qualitative evaluation (38%), isocenter frequency/transmitter gain or attenuation (35%) and geometric accuracy measurements (25%) (Fig. 4d).

### CT Imaging

Approximately one-third of survey participants (29%) use preclinical µCT imaging systems, with a broad distribution of systems: Molecubes (19%), Siemens Inveon (19%), Bruker Skyscan (17%), MILabs (14%), Mediso (8%) and a few others (11%). The majority of participants (53%) indicated yearly maintenance by the manufacturer, 18% reported maintenance more than once a year, 18% reported no regular maintenance, 6% less often than once per year and 6% did not know the maintenance frequency. Frequency of calibration was reported to be weekly (6%), monthly (26%), quarterly (9%), biannually (14%), yearly (23%) or without regular calibration (9%). For calibration or quality control, a Hounsfield-Unit (HU) phantom was most frequently used (57%), followed by a resolution phantom (29%), a geometry phantom (26%) and a bone mineral density phantom (17%) with 6% survey participants reporting that no phantom was used at all.

**Fig. 5.**
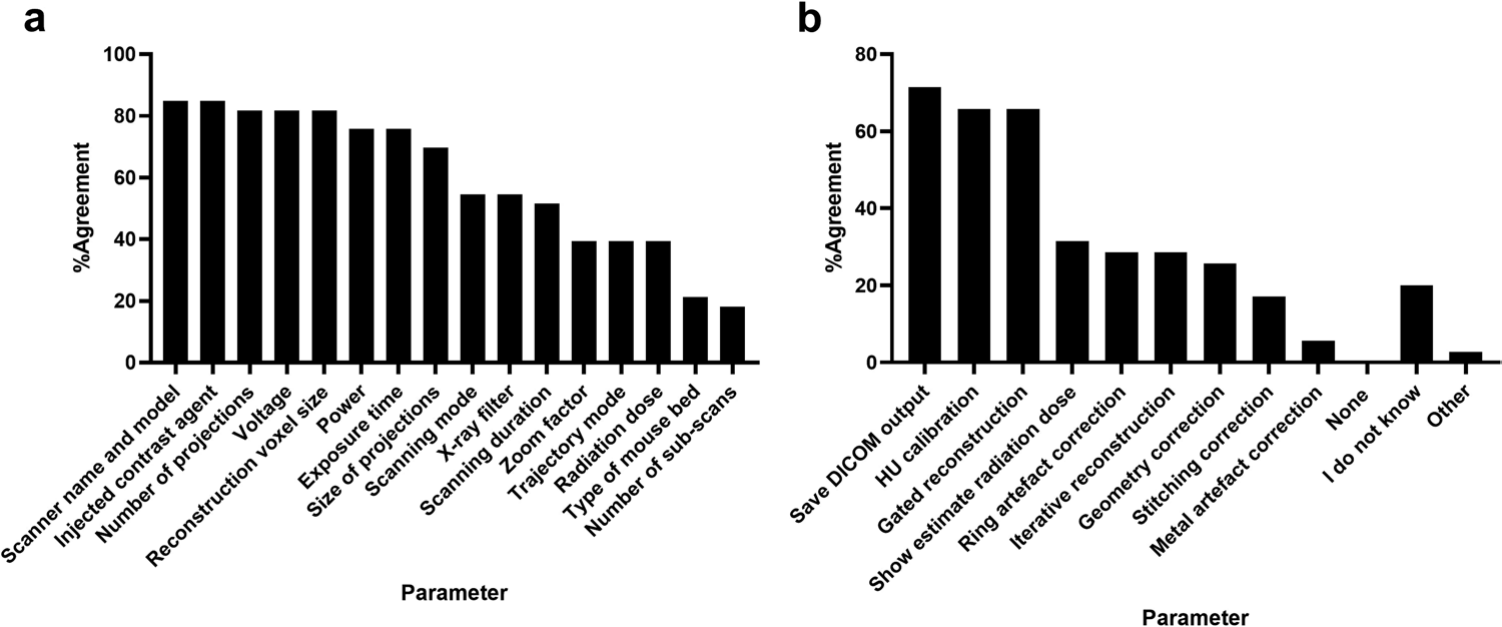
CT survey participants’ responses identify several important experimental descriptors that should be used when reporting CT imaging data in scientific publications **(a)**. Furthermore, results showed that many CT imaging systems are supplied with a basic reconstruction software without ring, metal, stitching, and geometry artefact correction and without an iterative reconstruction software **(b)**.

Participants found most of the suggested parameters important to report in a publication, i.e. 11 of the 16 parameters were checked by 50% or more participants (Fig. 5a). Feedback on available reconstruction software features indicated substantial room for improvement. While the majority of systems had DICOM output, HU-calibration, and gated reconstruction, most systems do not provide geometry, ring, metal or stitching artefact correction (Fig. 5b).

### Ultrasound Imaging

Survey results showed that only 13% of users were working with preclinical ultrasound devices with only 63% of these users using ultrasound scanners specifically designed for small animal imaging applications. Half of the ultrasound users stated that their scanners underwent annual scanner maintenance, while 44% of remaining users either did not know if the scanner underwent regular maintenance or the scanner had no regular maintenance. With respect to routine maintenance checks, over 75% of users routinely checked transducer cables and housing, 69% checked for transducer cracks and discolorations, while 50% routinely checked the images to detect dead transducer elements within the probes. Twenty-five percent of users did not know if any routine checks were performed on the scanner.

## Discussion

This survey clearly showed that there is nescience regarding standardization and guidelines in preclinical imaging research. Even though the ARRIVE guidelines have existed for more than 10 years [14], 47% of survey participants were unaware of them. In addition, guidelines on reporting preclinical imaging experiments [17] and small animal imaging quality control [18] have been available for many years (Table 1) but have not reached the right audience. For some modalities like optical imaging, other hurdles such as limited access or availability of phantoms, which are now emerging [19], further contribute to the lack of standardization [20]. Additionally, variability in preclinical imaging outcomes due to variations on acquisition protocols might still be observed even when a particular vendor dominates the market (e.g. preclinical MRI [21]).

**Table 1:** Available resources to support best-practice collection, analysis and publication of preclinical imaging data

Year of publication	Guidance provided/topic covered (in chronological order of publication)	Modality	Reference
2008	Scanner performance and standard (NEMA NU 4–2008)	PET	[29]
2010	ARRIVE guidelines for reporting animal research	All	[14]
2013	Methods descriptors used in preclinical imaging papers	All	[17]
2015	Animal handling, anaesthesia, QC and system performance	PET, SPECT	[30]
2016	Findable, Accessible, Interoperable, and Reusable (FAIR) data principles	All	[16]
2017	Small animal scanner QC	PET, SPECT, CT, MRI and optical	[18]
2018	PREPARE principles	All	[23]
2020	Latest 3R guidance, Arrive 2.0	All	[13]
2020	AQARA principles	PET, SPECT	[15]
2020	Standardisation of routine acquisition and reconstruction protocols	PET	[11]
2022	Preclinical scanner performance evaluation	Ultrasound	[31]
**Glossary** **3R:** The principles of the 3Rs (Replacement, Reduction and Refinement) were developed > 50 years ago and provide a framework for performing humane animal research **AQARA:** As quantitative as reasonably achievable (AQARA) principles propose standards for reporting radionuclide-based images in medical journals **ARRIVE:** The ARRIVE guidelines (Animal Research: Reporting of In Vivo Experiments) are a checklist of recommendations to improve the reporting of research involving animals **FAIR:** guidelines to improve the **F**indability, **A**ccessibility, **I**nteroperability, and **R**euse (FAIR) of digital assets **NEMA NU 4–2008:** National Electrical Manufacturers Association's (NEMA) NU 4–2008 standard specifies methodology for evaluating the performance of small animal PET scanners (not available since Oct 2022) **PREPARE:** Planning Research and Experimental Procedures on Animals: Recommendations for Excellence (PREPARE). It covers the three broad areas which determine the quality of the preparation for animal studies: formulation of the study, dialogue between scientists and the animal facility, and methods to be used for animal studies			

The ESMI standardization group was founded to tackle the awareness of standardization in preclinical imaging, which resulted in the publication of reviews [20] and scanner specific standardization studies, including phantoms and animals [11, 22]. Nevertheless, we have not reached the goal—which is the necessity of implementing standardization in preclinical imaging to obtain valid and reproducible images and data. Following from the data collected via the STANDARD survey and discussion at the EMIM 2022, it appears that this gap between available guidelines and the execution of these guidelines is related to the following aspects:

- Lack of Communication

The preclinical imaging field is a multidisciplinary field where biologists, chemists, physicists, pharmacists, physicians, veterinarians, bioengineers, computer scientists and many others work together. Depending on the training background of the people in charge of the imaging scanners, some guidelines will not be brought to their attention. For example, a physicist might be aware of the AQARA and QA/QC guideline but not of the ARRIVE guidelines, especially if coming from clinical imaging. Therefore, the preclinical imaging community would benefit from knowledge exchange between the different disciplines to promote the existing guidelines.

- Institutional Cost–Benefit

The cost–benefit analysis in standardization includes the number of person-months (PM) spent on scanner QA/QC, study protocol preparation (including the ARRIVE or PREPARE guidelines [13, 23]), image analysis and archiving. Standardization will not be performed if it is not clear that the benefit outweighs the costs. On the other hand, the benefits of embedding standardisation into preclinical imaging routines are reproducible and valid measurements and results. This would save an enormous amount of time (and money) by building on valid existing and published findings without duplicating studies.

- Community Standards and Regulatory Requirements

In clinical imaging, the external demand concerning standardization comes from regulatory aspects (e.g. radiation protection), funding agencies and publishers, and community-lead initiatives (e.g. EARL [24–26]). In preclinical imaging, the external demand concerning standardization was just recently started by some key journal publishers, who explicitly require the ARRIVE guidelines to be followed before submitting a scientific paper. Recently, following on from this survey results, expert panels composed of some members of the ESMI STANDARD group and the Physics Committee of the European Association of Nuclear Medicine (EANM) have initiated a collaboration to produce joint EANM-ESMI procedure guidelines for implementing an efficient preclinical PET and SPECT QC programme. We expect these guidelines will be published soon and anticipate that they will pave the way for standardisation of preclinical imaging modalities by fomenting other initiatives by expert panels in different preclinical imaging modalities.

- Translational Hiatus

Although preclinical imaging techniques like those covered in this paper (PET, SPECT, CT, US, MRI and optical imaging) are broadly translatable to the clinic, the requirements and recommendations to enhance reproducibility in a given clinical imaging protocol (e.g. MR neuroimaging, [27]), might not be directly translated to the preclinical environment due to additional cofounding factors such as animal handling and anaesthesia [28].

## Conclusions

Various resources are available to support efforts towards standardisation of preclinical imaging, many of which have been developed by members of the STANDARD team almost 10 years ago. Despite availability of said resources, the recently conducted STANDARD survey shows that standardisation of preclinical imaging techniques, wide implementation and use of QC/QA programmes and overall understanding of key guidelines in preclinical research (e.g. ARRIVE, FAIR) remain a challenge for the community. Important barriers to delivering standardisation efforts have been identified and wider dissemination of available tools alongside continued education of the community are needed to fully deliver on the preclinical standardisation promise.

## Supplementary Information

Below is the link to the electronic supplementary material.

Supplementary file1 (PDF 345 KB)

## Data Availability

All data generated or analysed during this study are included in this published article (and its supplementary information files).
