# Supplementary material for: Community Survey Results Show that Standardisation of Preclinical Imaging Techniques Remains a Challenge
Source: Mol Imaging Biol. 2022 Dec 8;25(3):560–8. doi: 10.1007/s11307-022-01790-6 (PMC10172263; doi:10.1007/s11307-022-01790-6)
Supplement: Supplementary file 1 — Supplementary file1 (PDF 345 KB) [file 11307_2022_1790_MOESM1_ESM.pdf]

Q1 Are there binding/recommended guidelines on QC/QA for preclinical scanner(s) in your institute?

Answered: 144 Skipped: 7

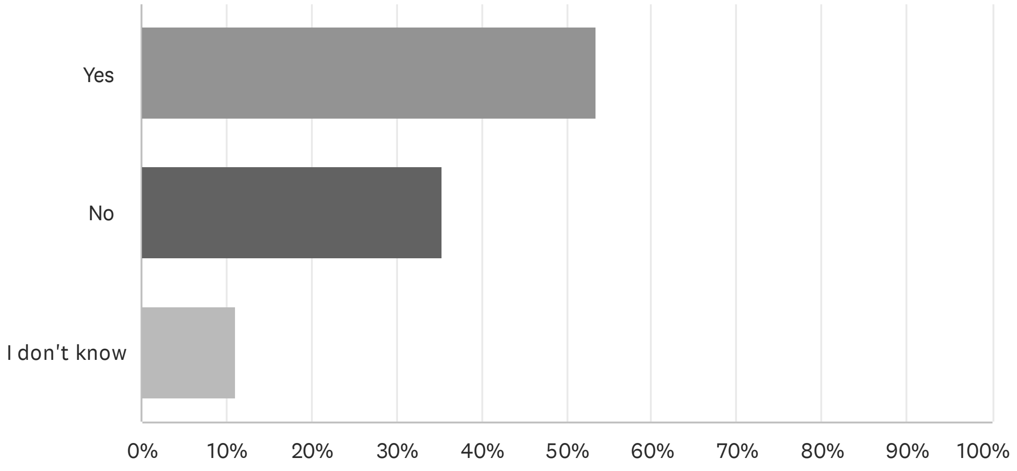

ANSWER CHOICES	RESPONSES	
Yes	53.47%	77
No	35.42%	51
I don't know	11.11%	16
TOTAL		144

Q2 Do you keep records of QC/QA performance of scanners, maintenance, and system failures?

Answered: 144 Skipped: 7

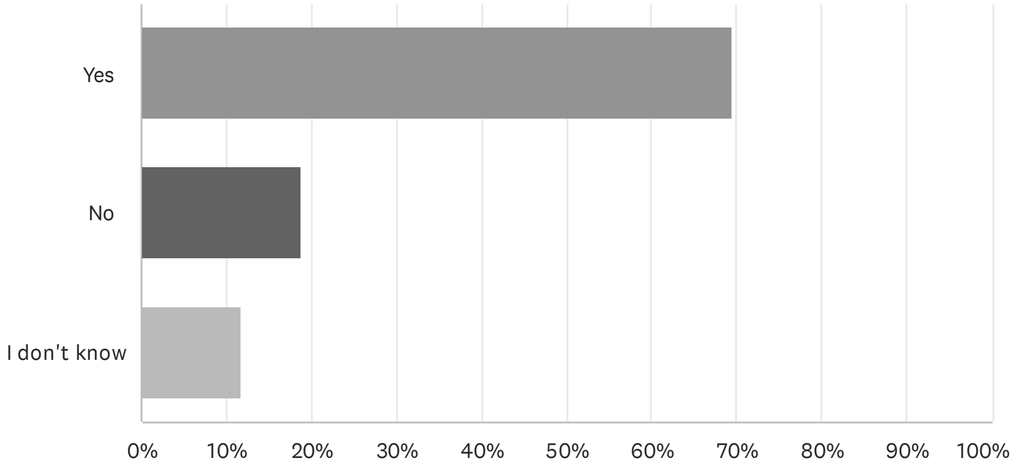

ANSWER CHOICES	RESPONSES	
Yes	69.44%	100
No	18.75%	27
I don't know	11.81%	17
TOTAL		144

Q3 How important do you consider a standardised accreditation for preclinical imaging scanners?

Answered: 117 Skipped: 34

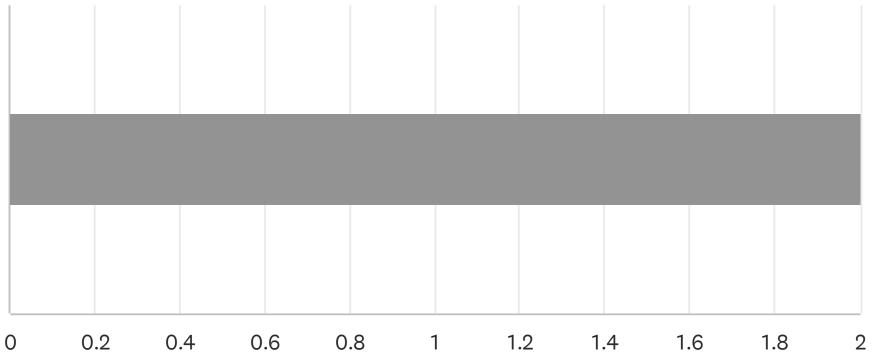

ANSWER CHOICES	AVERAGE NUMBER	TOTAL NUMBER	RESPONSES
	2	234	117
Total Respondents: 117			

#		DATE
1	2	1/26/2022 4:39 PM
2	3	1/26/2022 11:43 AM
3	1	1/26/2022 9:51 AM
4	1	1/26/2022 9:03 AM
5	1	1/25/2022 9:25 AM
6	1	1/24/2022 8:13 PM
7	3	1/24/2022 2:33 PM
8	4	1/24/2022 2:12 PM
9	1	1/24/2022 2:03 PM
10	1	1/24/2022 11:38 AM
11	1	1/24/2022 11:35 AM
12	3	1/24/2022 11:25 AM
13	2	1/24/2022 8:51 AM
14	2	1/24/2022 8:21 AM
15	1	1/22/2022 3:05 PM
16	1	1/22/2022 10:17 AM
17	3	1/21/2022 4:12 PM
18	2	1/21/2022 2:19 PM
19	3	1/21/2022 2:12 PM
20	3	1/21/2022 2:09 PM
21	2	1/21/2022 2:09 PM
22	1	1/21/2022 2:08 PM

23	2	1/21/2022 1:49 PM
24	1	1/21/2022 1:41 PM
25	1	1/21/2022 1:34 PM
26	4	1/21/2022 1:28 PM
27	3	1/18/2022 11:15 AM
28	3	1/17/2022 10:21 AM
29	1	1/15/2022 9:52 PM
30	1	1/12/2022 8:30 AM
31	1	1/5/2022 1:44 PM
32	1	1/3/2022 12:03 PM
33	3	12/22/2021 8:53 AM
34	1	12/21/2021 12:02 PM
35	3	12/21/2021 10:35 AM
36	3	12/20/2021 11:56 PM
37	1	12/20/2021 10:20 AM
38	2	12/16/2021 5:28 PM
39	2	12/16/2021 9:13 AM
40	1	12/16/2021 8:26 AM
41	2	12/14/2021 3:57 PM
42	1	12/14/2021 1:29 PM
43	2	12/14/2021 11:48 AM
44	1	12/14/2021 10:35 AM
45	1	12/14/2021 9:23 AM
46	3	12/14/2021 9:21 AM
47	3	12/13/2021 6:41 PM
48	1	12/13/2021 3:49 PM
49	1	12/13/2021 3:40 PM
50	2	12/13/2021 3:35 PM
51	1	12/13/2021 2:28 PM
52	4	12/13/2021 1:23 PM
53	1	12/13/2021 12:25 PM
54	2	12/13/2021 11:44 AM
55	1	12/13/2021 10:56 AM
56	2	12/13/2021 10:48 AM
57	1	12/13/2021 10:17 AM
58	4	12/13/2021 9:48 AM
59	1	12/13/2021 9:48 AM
60	1	12/13/2021 9:17 AM
61	2	12/13/2021 9:16 AM
62	2	12/13/2021 8:32 AM
63	1	12/13/2021 7:28 AM

64	2	12/13/2021 6:48 AM
65	2	12/12/2021 10:07 PM
66	3	12/12/2021 5:36 PM
67	3	12/12/2021 1:59 AM
68	3	12/12/2021 1:25 AM
69	1	12/11/2021 2:16 PM
70	3	12/11/2021 8:19 AM
71	1	12/11/2021 6:14 AM
72	2	12/10/2021 8:30 PM
73	2	12/10/2021 6:51 PM
74	3	12/10/2021 6:41 PM
75	1	12/10/2021 6:06 PM
76	3	12/10/2021 4:45 PM
77	1	12/10/2021 4:43 PM
78	4	12/10/2021 4:23 PM
79	2	12/10/2021 4:21 PM
80	1	12/10/2021 4:02 PM
81	2	12/10/2021 3:57 PM
82	1	12/10/2021 3:46 PM
83	2	12/10/2021 3:40 PM
84	2	12/10/2021 3:19 PM
85	3	12/10/2021 3:16 PM
86	3	12/10/2021 3:08 PM
87	4	12/10/2021 3:05 PM
88	1	12/10/2021 2:45 PM
89	4	12/10/2021 2:39 PM
90	3	12/10/2021 2:33 PM
91	2	12/10/2021 2:26 PM
92	3	12/10/2021 2:26 PM
93	2	12/10/2021 2:26 PM
94	2	12/10/2021 2:21 PM
95	1	12/10/2021 2:13 PM
96	2	12/10/2021 2:12 PM
97	2	12/10/2021 2:10 PM
98	2	12/10/2021 2:09 PM
99	4	12/10/2021 1:58 PM
100	3	12/10/2021 1:49 PM
101	1	12/10/2021 1:46 PM
102	2	12/10/2021 1:45 PM
103	2	12/10/2021 1:44 PM
104	3	12/10/2021 1:44 PM

105	2	12/10/2021 1:43 PM
106	1	12/10/2021 1:41 PM
107	2	12/10/2021 1:38 PM
108	3	12/10/2021 1:35 PM
109	1	12/10/2021 1:35 PM
110	1	12/10/2021 1:34 PM
111	3	12/10/2021 1:33 PM
112	4	12/10/2021 1:33 PM
113	1	12/10/2021 1:31 PM
114	2	12/10/2021 1:29 PM
115	2	12/10/2021 1:29 PM
116	2	12/10/2021 1:11 PM
117	1	12/10/2021 12:56 PM

Q4 Where do you store your raw and processed imaging data?(multiple answers possible)

Answered: 128 Skipped: 23

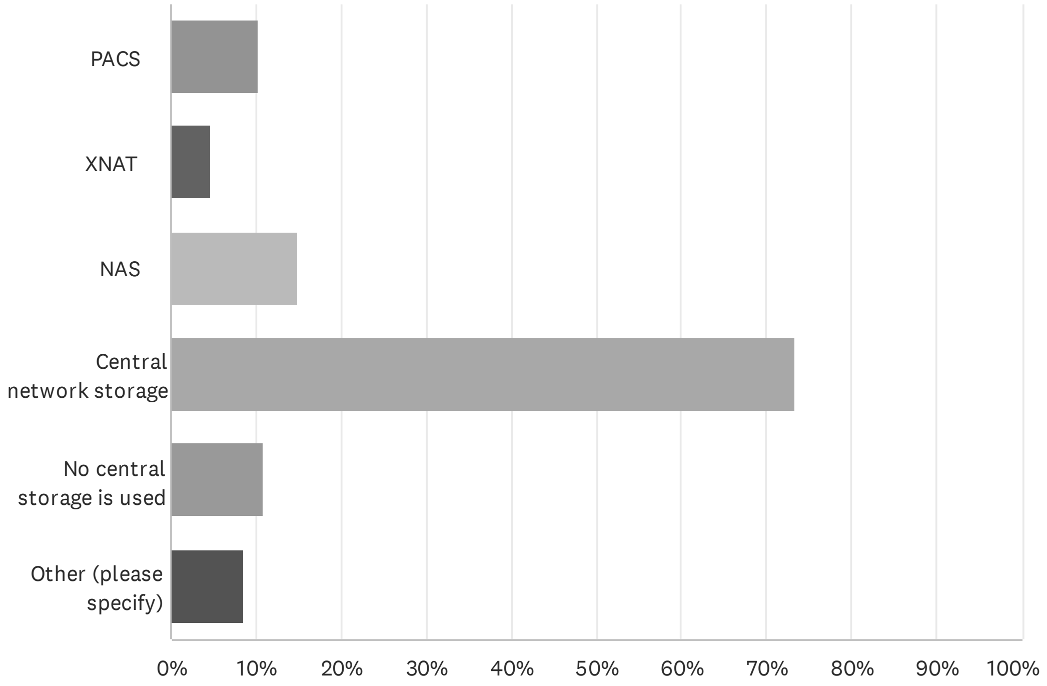

ANSWER CHOICES	RESPONSES	
PACS	10.16%	13
XNAT	4.69%	6
NAS	14.84%	19
Central network storage	73.44%	94
No central storage is used	10.94%	14
Other (please specify)	8.59%	11
Total Respondents: 128		

#	OTHER (PLEASE SPECIFY)	DATE
1	XNAT is under consideration for part of the scanners, but not for multiphoton microscopy data, which is currently stored locally on the network and on drives. We are considering storage in anDREa, a platform embedded in Microsoft Azure.	1/26/2022 9:08 AM
2	Scanner PC	1/24/2022 11:37 AM
3	Dedicated offline PC	1/10/2022 6:17 PM
4	backup in external drives	12/13/2021 3:50 PM
5	FAST archive	12/13/2021 10:27 AM
6	RAID System (5 Harddrives)	12/10/2021 7:05 PM
7	encrypted object storage	12/10/2021 7:04 PM
8	acquisition PC temporarily	12/10/2021 6:06 PM

9	National Research Counsil	12/10/2021 2:52 PM
10	external hard disks	12/10/2021 2:14 PM
11	external drive	12/10/2021 1:35 PM

Q5 Which data format do you use?(multiple answers possible)

Answered: 127 Skipped: 24

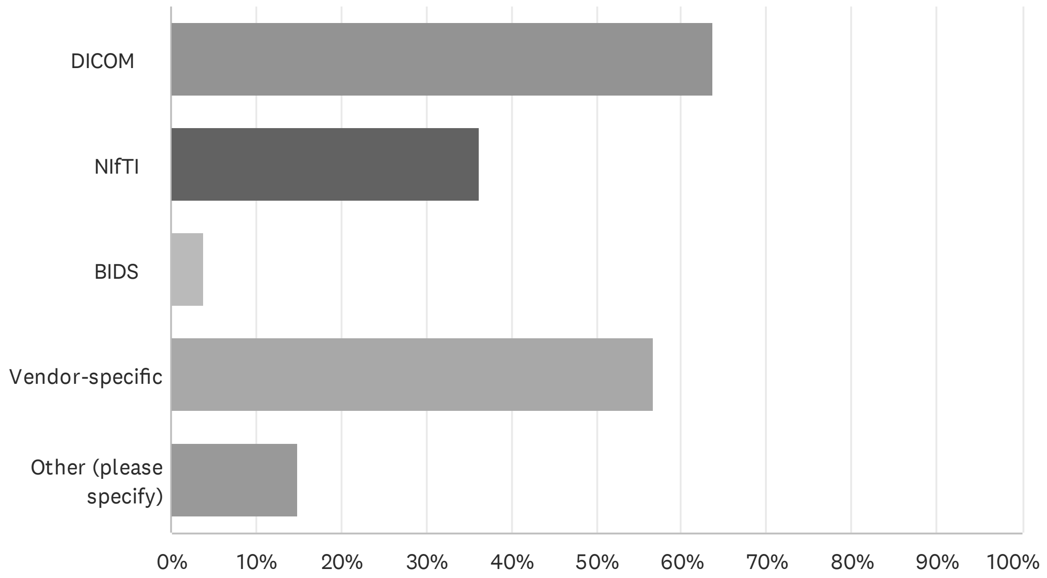

ANSWER CHOICES	RESPONSES	
DICOM	63.78%	81
NIfTI	36.22%	46
BIDS	3.94%	5
Vendor-specific	56.69%	72
Other (please specify)	14.96%	19
Total Respondents: 127		

#	OTHER (PLEASE SPECIFY)	DATE
1	ome.tif for the microscopy preclinical data	1/26/2022 9:08 AM
2	TIF, JPG, PNG	1/24/2022 8:54 AM
3	Matlab	1/5/2022 5:18 PM
4	Self format	12/14/2021 9:22 AM
5	HDF5	12/13/2021 4:52 PM
6	interfile	12/13/2021 10:57 AM
7	bip	12/13/2021 9:18 AM
8	CZI, OME-TIFF	12/10/2021 7:04 PM
9	ECAT .v	12/10/2021 6:06 PM
10	Matlab	12/10/2021 4:44 PM
11	raw data (Siemens format)	12/10/2021 3:20 PM
12	Research data analysys	12/10/2021 2:52 PM
13	tiff, bmp	12/10/2021 2:27 PM
14	BIQ (custom format)	12/10/2021 2:12 PM

15	I don't know	12/10/2021 1:48 PM
16	matlab	12/10/2021 1:46 PM
17	matlab file or tiff	12/10/2021 1:32 PM
18	Interfile	12/10/2021 1:29 PM
19	listmode	12/10/2021 1:12 PM

Q6 Do you use a Standard Operating Procedure (SOP) for(multiple answers possible)

Answered: 128 Skipped: 23

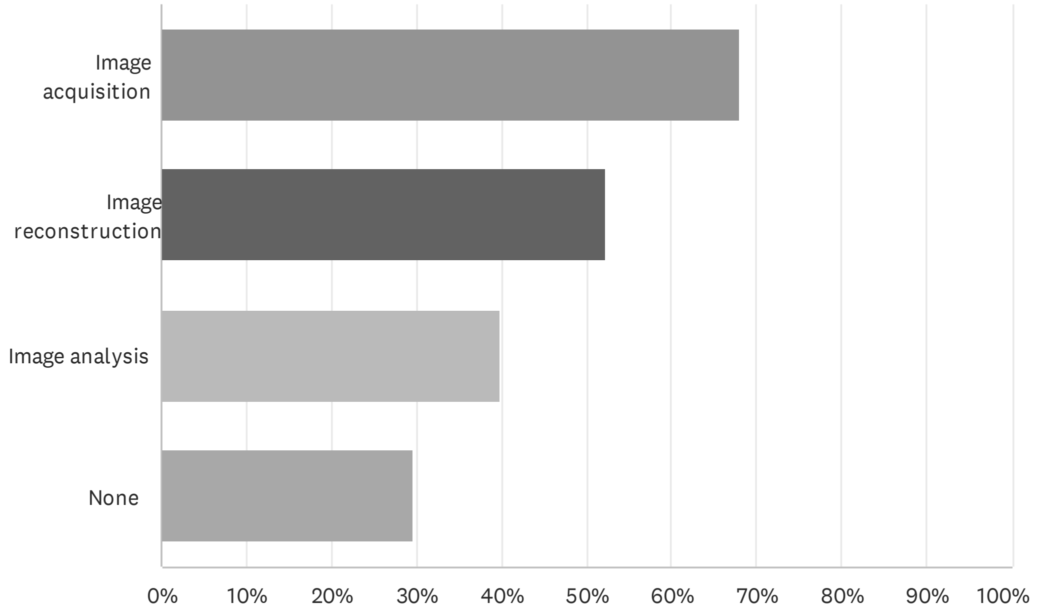

ANSWER CHOICES	RESPONSES	
Image acquisition	67.97%	87
Image reconstruction	52.34%	67
Image analysis	39.84%	51
None	29.69%	38
Total Respondents: 128		

Q7 Which processing tools do you use e.g. MATLAB, PMod, Vivoquant, ImageJ, Analyze, Vendor's software?

Answered: 119 Skipped: 32

#	RESPONSES	DATE
1	ImageJ, Osirix, AMIDE, Imalytics	1/27/2022 11:03 AM
2	ImageJ, Vendor's software, AMIDE, Slicer	1/26/2022 11:14 PM
3	Matlab	1/26/2022 4:39 PM
4	Pmod	1/26/2022 11:44 AM
5	Imalytics	1/26/2022 9:52 AM
6	For multiphoton intravital microscopy: ImageJ, Arivis 4D packages, Matlab.	1/26/2022 9:08 AM
7	MATLAB, ImageJ	1/25/2022 9:26 AM
8	Matlab; imagej; living image	1/24/2022 8:15 PM
9	Inveon	1/24/2022 5:45 PM
10	matlab, python	1/24/2022 2:13 PM
11	PMod, ImageJ	1/24/2022 2:04 PM
12	Matlab, ImageJ, Vendor's software	1/24/2022 11:37 AM
13	Vendors software	1/24/2022 11:27 AM
14	ImageJ, KNIME, python, R, 3Dslicer	1/24/2022 8:54 AM
15	ImageJ, PMod, Osirix, Amide, Vendor's software	1/24/2022 8:22 AM
16	Pmod	1/22/2022 10:18 AM
17	MATLAB, Vendor,ImageJ,Imalytics	1/21/2022 2:13 PM
18	MATLAB, PMOD, ImageJ, Inveon (Siemens)	1/21/2022 2:10 PM
19	Matlab, SPM12, MagnAn	1/21/2022 2:09 PM
20	ImageJ, TARQUIN, python	1/21/2022 1:45 PM
21	what ever works pmod, matlab etc	1/21/2022 1:38 PM
22	Pmod, ImageJ (Fiji), ITK-SNAP	1/21/2022 1:35 PM
23	Matlab, Pmod, Vivoquant, vendor software, python	1/21/2022 1:29 PM
24	ImageJ, Pmod, MATLAB, PV, Mevislab, ...	1/18/2022 11:17 AM
25	vivoquant, interview fusion, MMWKS	1/17/2022 10:22 AM
26	MATLAB, PMod, Vivoquant, ImageJ, Paravision, Top spin, Interview fusion	1/17/2022 9:40 AM
27	PMOD, Vivoquant, python	1/15/2022 9:53 PM
28	PMOD	1/12/2022 8:31 AM
29	Vendor's software	1/10/2022 6:17 PM
30	Matlab, ImageJ	1/5/2022 5:18 PM
31	PMod,Amide	1/5/2022 1:45 PM
32	ParaVision	1/3/2022 12:27 PM
33	Paravision, Matlab, ImageJ, Amira, DTIStudio	12/22/2021 8:55 AM
34	pmod, spm-matlab	12/21/2021 12:02 PM

35	PMOD, Vendor's software	12/21/2021 10:36 AM
36	MATLAb	12/20/2021 11:59 PM
37	Vendor's software	12/16/2021 5:28 PM
38	MATLAB, ImageJ	12/16/2021 12:19 PM
39	Vendor's software, Matlab, SPM, Image J	12/16/2021 11:26 AM
40	ANTs, FSL, ITKsnap, python, ImageJ, Slicer	12/16/2021 8:27 AM
41	pmod, IRW, matlab, spm, imagej, self-written code	12/14/2021 3:57 PM
42	ImageJ, ParaVision	12/14/2021 1:30 PM
43	Vivoquant	12/14/2021 11:49 AM
44	Medis Suite, ImageJ Plug-in	12/14/2021 9:24 AM
45	matlab, Image J	12/14/2021 9:22 AM
46	ImageJ, MATLAB, Vendor's software	12/13/2021 6:43 PM
47	MATLAB, Python, ImageJ, Vendors software	12/13/2021 4:52 PM
48	PMod	12/13/2021 3:50 PM
49	vivoquant	12/13/2021 3:41 PM
50	Matlab, ImageJ, PMOD, Mevislab, FSL, ITKsnap,	12/13/2021 3:37 PM
51	MATLAB, PMod, Living Image, ImageJ, Vendor's software: Inveon Research Workplace	12/13/2021 2:28 PM
52	vivoquant and interview-fusion	12/13/2021 1:45 PM
53	Python, ImageJ, FSL, MATLAB	12/13/2021 1:24 PM
54	MATLAB, SPM, FSL, MRTRIX,...	12/13/2021 12:26 PM
55	ImageJ	12/13/2021 11:47 AM
56	pmod, vivoquant, imalytics, analyse	12/13/2021 11:45 AM
57	ImageJ, PMOD	12/13/2021 10:57 AM
58	MATLAB, ImageJ, FSL, MRTrix, AFNI	12/13/2021 10:50 AM
59	PMOD, IRW, SPM, Vendor's software, Munich Heart	12/13/2021 10:27 AM
60	ImageJ, Inveon Research Workstation (vendor)	12/13/2021 10:18 AM
61	MATLAB, LCModel, Python, ImageJ	12/13/2021 8:47 AM
62	AMIDE, ImageJ, PMOD	12/13/2021 8:41 AM
63	FSL, BrainVoyager, SPM, PMod, PV7.0	12/13/2021 7:30 AM
64	ANTS, FSL, MATLAB	12/13/2021 6:49 AM
65	Vendor's software	12/12/2021 5:37 PM
66	PMod, Vendor's software	12/12/2021 2:01 AM
67	Vivoquant, ImagJ	12/12/2021 1:26 AM
68	Matlab and ImageJ	12/11/2021 6:04 PM
69	Python, ImageJ	12/11/2021 2:17 PM
70	Matlab, python, R, imageJ	12/11/2021 8:19 AM
71	AFNI	12/11/2021 6:14 AM
72	All the above and other. Imaris, etc	12/10/2021 8:31 PM
73	PMod	12/10/2021 7:05 PM
74	MATLAB, ImageJ, Python, Arivis 4D	12/10/2021 7:04 PM
75	PMod, ImageJ	12/10/2021 6:52 PM

76	PMOD, Vivoquant, ImageJ, Bruker	12/10/2021 6:42 PM
77	Vendor's software, Brainvisa/Anatomist	12/10/2021 6:06 PM
78	PMod	12/10/2021 4:46 PM
79	Matlab	12/10/2021 4:44 PM
80	ParaVision, ImageJ	12/10/2021 4:21 PM
81	imageJ, matlab	12/10/2021 3:57 PM
82	MATLAB	12/10/2021 3:47 PM
83	Vendor's software	12/10/2021 3:40 PM
84	IRW	12/10/2021 3:37 PM
85	Matlab, ImageJ	12/10/2021 3:20 PM
86	Matlab, ImageJ	12/10/2021 3:17 PM
87	MATLAB, ImageJ, Analyze, Living Image	12/10/2021 3:08 PM
88	FSL, MatLab, CVI	12/10/2021 3:06 PM
89	Adalta Wolfram	12/10/2021 2:52 PM
90	Vendor's software + locally developed tools	12/10/2021 2:46 PM
91	Matlab, pmod, imagej	12/10/2021 2:40 PM
92	ImageJ, vendor's software, matlab... many, depending on the application and experience of user	12/10/2021 2:27 PM
93	MATLAB, FSL, SPM, AFNI	12/10/2021 2:27 PM
94	PMOD	12/10/2021 2:14 PM
95	pmod , in house routines	12/10/2021 2:13 PM
96	Matlab	12/10/2021 2:12 PM
97	MATLAB	12/10/2021 2:10 PM
98	Matlab	12/10/2021 2:09 PM
99	Xstrahl murislice/muriplan	12/10/2021 2:01 PM
100	Malab, Python, VivoQuant, Image J	12/10/2021 1:58 PM
101	ImageJ, VGStudio, MatLab, self developed software	12/10/2021 1:50 PM
102	I make optical imaging and I use M3vision and 3DViewer which is dictated by optical system producer .	12/10/2021 1:48 PM
103	Vivoquant, Horos	12/10/2021 1:46 PM
104	Vivoquant, PMOD, Vendors software	12/10/2021 1:46 PM
105	Matlab	12/10/2021 1:46 PM
106	Matlab, Vivoquant, Fusion, PMOD	12/10/2021 1:45 PM
107	python	12/10/2021 1:44 PM
108	MATLAB, ImageJ, multiple Vendor Software, Osirix/Horos, ICY, DTI-Studio, Imalytics, Segment	12/10/2021 1:41 PM
109	Vivoquant	12/10/2021 1:39 PM
110	mod	12/10/2021 1:35 PM
111	Vendors software, Image J	12/10/2021 1:35 PM
112	Amide, pmod	12/10/2021 1:35 PM
113	MATLAB, ImageJ, Vendor's Software	12/10/2021 1:35 PM
114	VivoquANT	12/10/2021 1:34 PM

115	MATLAB	12/10/2021 1:32 PM
116	ImageJ, QuPath, Vendors software, Imalytics	12/10/2021 1:30 PM
117	Matlab, PMOD, AMIDE, 3D Slicer	12/10/2021 1:29 PM
118	PMOD, MATLAB	12/10/2021 1:12 PM
119	Inveon	12/10/2021 12:57 PM

Q8 When publishing your data, do you report on:(tick all items of relevance)

Answered: 127 Skipped: 24

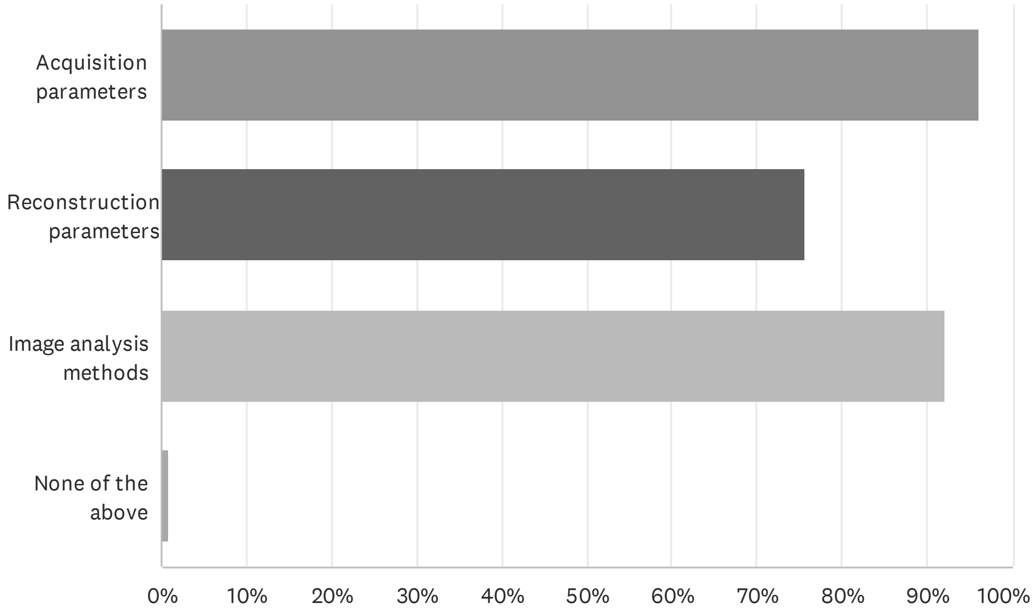

ANSWER CHOICES	RESPONSES	
Acquisition parameters	96.06%	122
Reconstruction parameters	75.59%	96
Image analysis methods	92.13%	117
None of the above	0.79%	1
Total Respondents: 127		

Q9 How important do you consider that a (medical) journal requires authors to report/state on QC/QA programmes used at their imaging centre?

Answered: 93 Skipped: 58

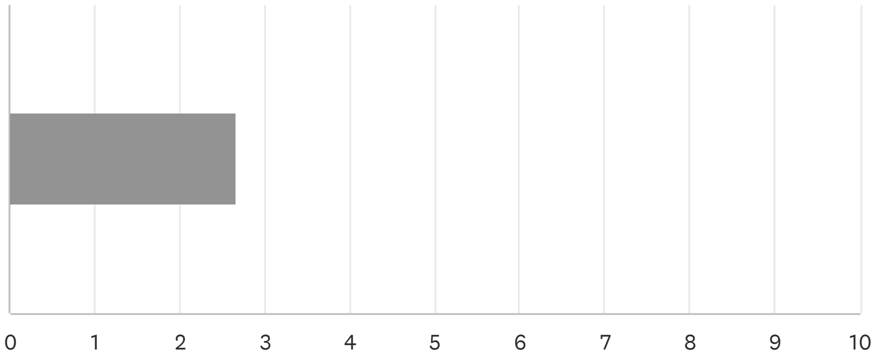

ANSWER CHOICES	AVERAGE NUMBER	TOTAL NUMBER	RESPONSES
	3	248	93
Total Respondents: 93			

#		DATE
1	3	1/27/2022 11:04 AM
2	5	1/26/2022 11:45 AM
3	2	1/26/2022 9:53 AM
4	1	1/26/2022 9:11 AM
5	3	1/24/2022 8:16 PM
6	3	1/24/2022 2:14 PM
7	1	1/24/2022 2:05 PM
8	2	1/24/2022 11:39 AM
9	3	1/24/2022 11:30 AM
10	2	1/24/2022 8:56 AM
11	2	1/24/2022 8:23 AM
12	4	1/21/2022 2:21 PM
13	1	1/21/2022 2:14 PM
14	1	1/21/2022 2:12 PM
15	3	1/21/2022 1:52 PM
16	3	1/21/2022 1:38 PM
17	3	1/21/2022 1:35 PM
18	5	1/21/2022 1:31 PM
19	4	1/18/2022 11:18 AM
20	3	1/17/2022 12:21 PM
21	3	1/17/2022 10:25 AM

22	3	1/17/2022 9:42 AM
23	1	1/5/2022 5:19 PM
24	3	12/22/2021 8:56 AM
25	2	12/21/2021 12:02 PM
26	5	12/21/2021 12:01 AM
27	4	12/16/2021 5:30 PM
28	3	12/16/2021 12:27 PM
29	3	12/16/2021 11:29 AM
30	5	12/16/2021 8:29 AM
31	1	12/15/2021 10:48 AM
32	2	12/14/2021 3:59 PM
33	3	12/14/2021 1:31 PM
34	2	12/14/2021 12:03 PM
35	3	12/14/2021 9:23 AM
36	1	12/13/2021 6:44 PM
37	2	12/13/2021 3:53 PM
38	2	12/13/2021 3:48 PM
39	4	12/13/2021 3:39 PM
40	3	12/13/2021 1:26 PM
41	3	12/13/2021 12:27 PM
42	2	12/13/2021 11:49 AM
43	3	12/13/2021 10:58 AM
44	3	12/13/2021 10:54 AM
45	2	12/13/2021 10:28 AM
46	3	12/13/2021 9:19 AM
47	3	12/13/2021 8:51 AM
48	3	12/13/2021 8:43 AM
49	3	12/13/2021 6:50 AM
50	3	12/12/2021 5:38 PM
51	3	12/12/2021 2:07 AM
52	3	12/12/2021 1:52 AM
53	3	12/11/2021 6:05 PM
54	1	12/11/2021 2:18 PM
55	3	12/11/2021 8:21 AM
56	1	12/11/2021 6:14 AM
57	3	12/11/2021 3:12 AM
58	3	12/10/2021 6:46 PM
59	2	12/10/2021 6:06 PM
60	3	12/10/2021 4:26 PM
61	2	12/10/2021 4:21 PM
62	2	12/10/2021 3:59 PM

63	3	12/10/2021 3:41 PM
64	3	12/10/2021 3:41 PM
65	2	12/10/2021 3:28 PM
66	3	12/10/2021 3:19 PM
67	3	12/10/2021 3:11 PM
68	1	12/10/2021 3:07 PM
69	3	12/10/2021 2:57 PM
70	1	12/10/2021 2:48 PM
71	3	12/10/2021 2:44 PM
72	4	12/10/2021 2:28 PM
73	3	12/10/2021 2:28 PM
74	4	12/10/2021 2:28 PM
75	3	12/10/2021 2:15 PM
76	2	12/10/2021 2:15 PM
77	1	12/10/2021 2:13 PM
78	3	12/10/2021 2:12 PM
79	5	12/10/2021 2:11 PM
80	3	12/10/2021 1:59 PM
81	3	12/10/2021 1:51 PM
82	3	12/10/2021 1:50 PM
83	4	12/10/2021 1:49 PM
84	2	12/10/2021 1:49 PM
85	3	12/10/2021 1:48 PM
86	2	12/10/2021 1:45 PM
87	1	12/10/2021 1:43 PM
88	2	12/10/2021 1:40 PM
89	1	12/10/2021 1:37 PM
90	3	12/10/2021 1:37 PM
91	3	12/10/2021 1:37 PM
92	3	12/10/2021 1:36 PM
93	2	12/10/2021 1:31 PM

Q10 Do you archive your published raw imaging data?

Answered: 126 Skipped: 25

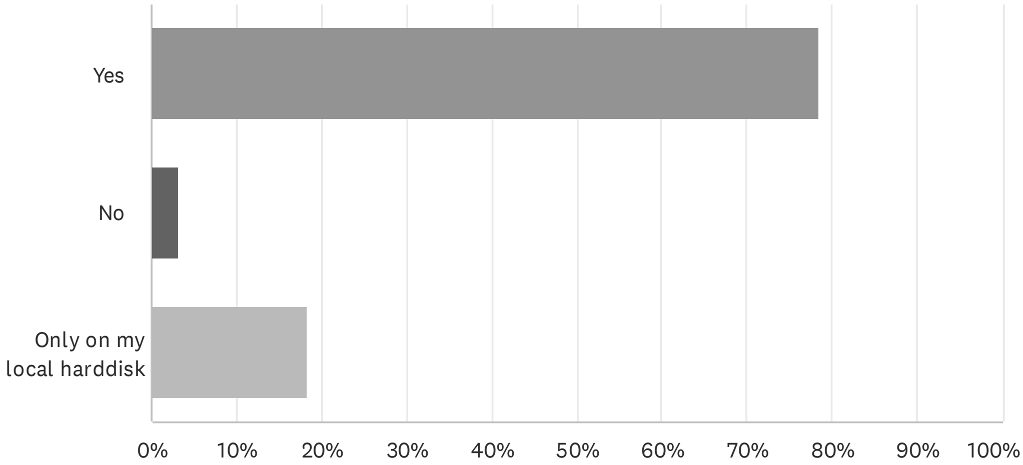

ANSWER CHOICES	RESPONSES	
Yes	78.57%	99
No	3.17%	4
Only on my local harddisk	18.25%	23
TOTAL		126

Q11 Would you agree to publish details of your acquisition/processing protocols in accordance with a community-lead consensus procedure?

Answered: 127 Skipped: 24

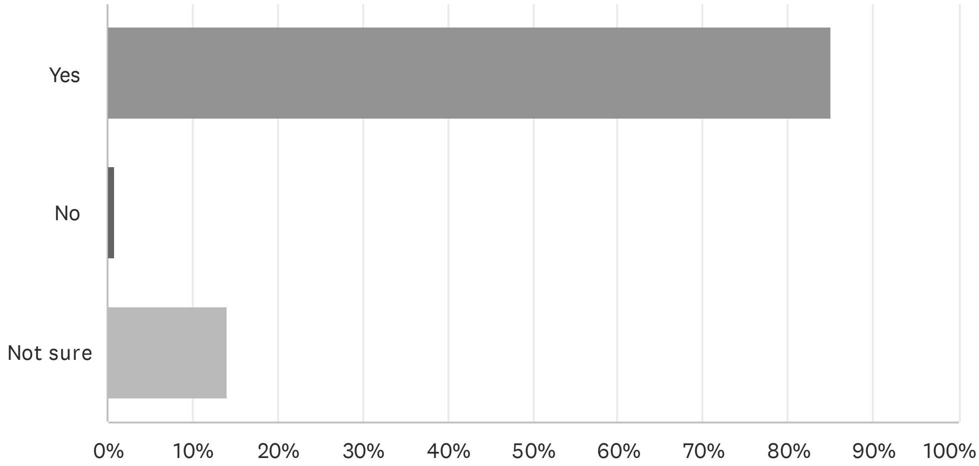

ANSWER CHOICES	RESPONSES	
Yes	85.04%	108
No	0.79%	1
Not sure	14.17%	18
TOTAL		127

Q12 When publishing, do you follow certain reporting guidelines for in vivo imaging experiments in animals e.g. ARRIVE*?The ARRIVE guidelines (Animal Research: Reporting of In Vivo Experiments) are a checklist of recommendations to improve the reporting of research involving animals originally published in PLOS Biology, July 2020. https://arriveguidelines.org/

Answered: 127 Skipped: 24

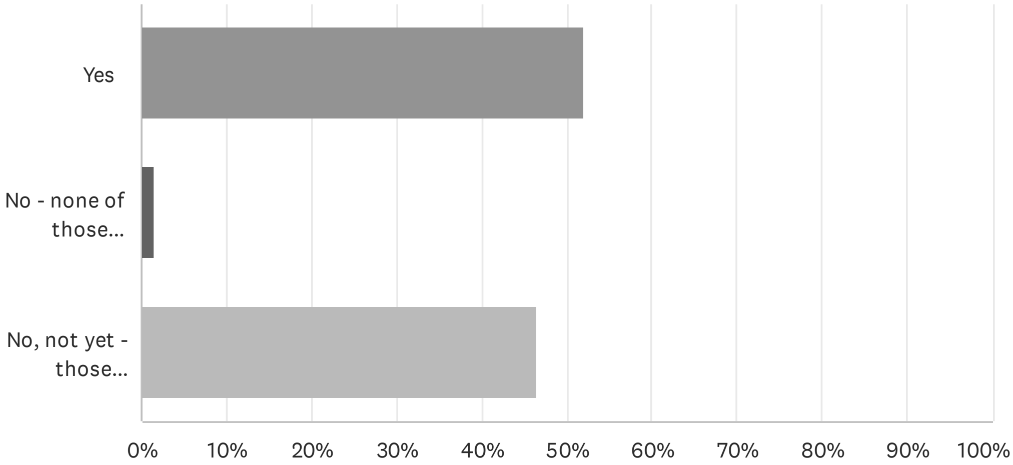

ANSWER CHOICES	RESPONSES	
Yes	51.97%	66
No - none of those guidelines are useful	1.57%	2
No, not yet - those guidelines just came to my attention	46.46%	59
TOTAL		127

Q13 Do you or would you agree to follow the AQARA* Requirements for Radionuclide-Based Images?*The AQARA principle – proposing standard requirements for radionuclide-based images in medical journals. J Nucl Med. 2020;61(1):1-2 https://jnm.snmjournals.org/AQARA

Answered: 126 Skipped: 25

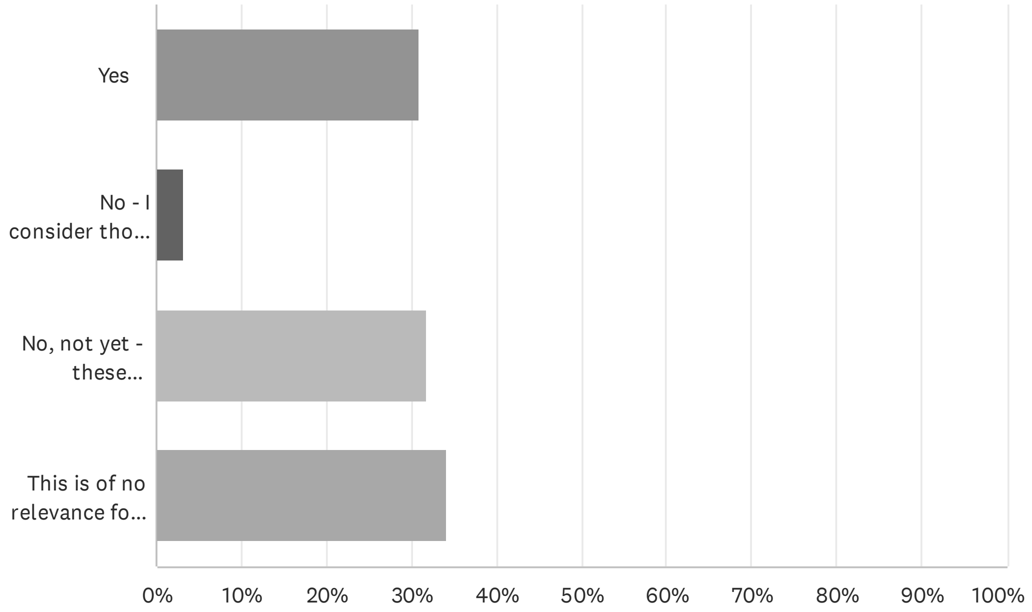

ANSWER CHOICES	RESPONSES	
Yes	30.95%	39
No - I consider those requirements as not useful	3.17%	4
No, not yet - these guidelines just came to my attention	31.75%	40
This is of no relevance for my research.	34.13%	43
TOTAL		126

Q14 Do you share your imaging data publicly (in accordance to established guidelines*)?*e.g. The FAIR Guiding Principles for scientific data management and stewardship published in Scientific Data in 2016 www.nature.com/articles/sdata201618

Answered: 127 Skipped: 24

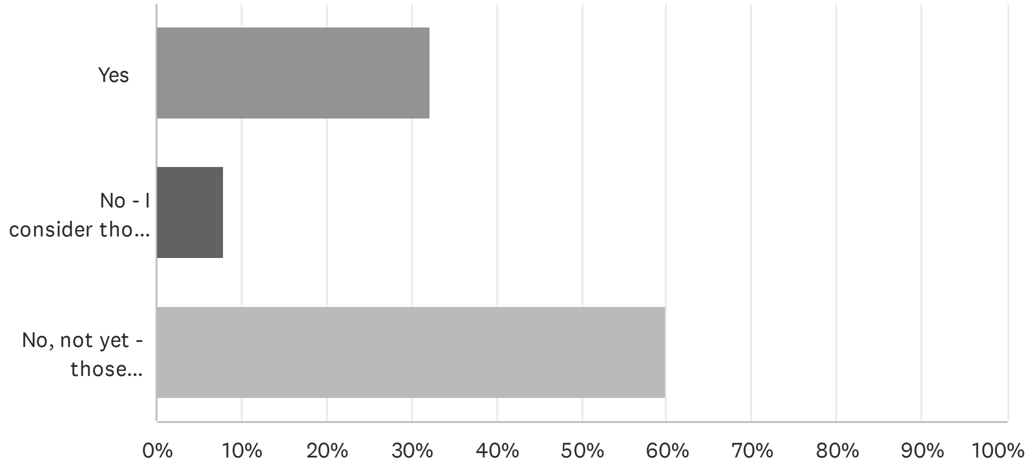

ANSWER CHOICES	RESPONSES	
Yes	32.28%	41
No - I consider those guidelines as not useful	7.87%	10
No, not yet - those guidelines just came to my attention	59.84%	76
TOTAL		127

Q15 Are you working with/using preclinical Ultrasound Device(s)?

Answered: 133 Skipped: 18

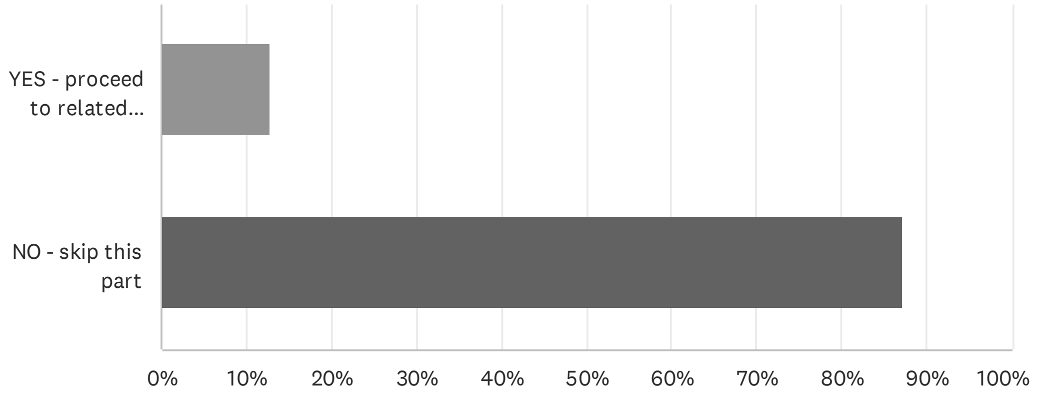

ANSWER CHOICES	RESPONSES	
YES - proceed to related questions	12.78%	17
NO - skip this part	87.22%	116
Total Respondents: 133		

Q16 Which ultrasound scanner(s)/transducers do you use for preclinical ultrasound scanning?

Answered: 16 Skipped: 135

#	RESPONSES	DATE
1	Vevo 770, Vevo 2100, Sharp T1	1/27/2022 2:08 AM
2	vevo2100, vevo 3100	1/21/2022 2:23 PM
3	Vevo 3000	1/21/2022 2:16 PM
4	fuji	1/21/2022 1:41 PM
5	Verasonics	1/5/2022 5:20 PM
6	Vevo 3100 MX550D	12/16/2021 5:33 PM
7	Iconeus	12/16/2021 11:31 AM
8	AUTC fUSI scanner	12/14/2021 9:24 AM
9	vevo 2100	12/13/2021 1:52 PM
10	GE	12/13/2021 8:51 AM
11	vevo	12/10/2021 2:30 PM
12	Aixplorer and Verasonics	12/10/2021 2:15 PM
13	Vevo 2100	12/10/2021 2:00 PM
14	Siemens Juniper, 11M3, 18H5, 8V4	12/10/2021 1:57 PM
15	Fuji-Visualsonic 2100/LAZR	12/10/2021 1:46 PM
16	Fujifilm's Vevo 2100 and 3100	12/10/2021 1:32 PM

Q17 What is the frequency of regular scanner-maintenance by the manufacturer?

Answered: 16 Skipped: 135

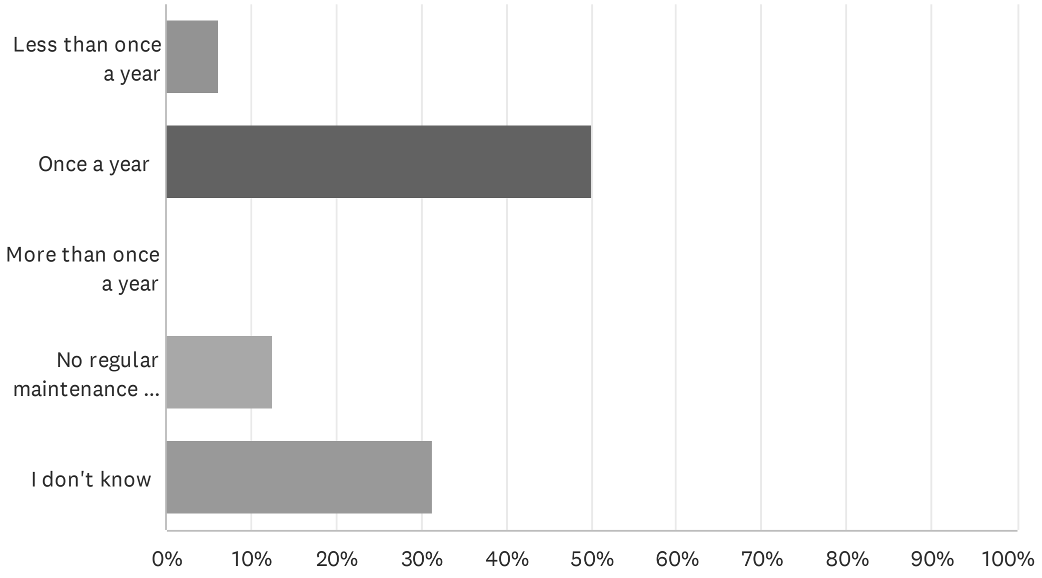

ANSWER CHOICES	RESPONSES	
Less than once a year	6.25%	1
Once a year	50.00%	8
More than once a year	0.00%	0
No regular maintenance by the manfacturer	12.50%	2
I don't know	31.25%	5
TOTAL		16

Q18 As part of your routine checks, do you* regularly...*you, your colleagues, or service engineer

Answered: 16 Skipped: 135

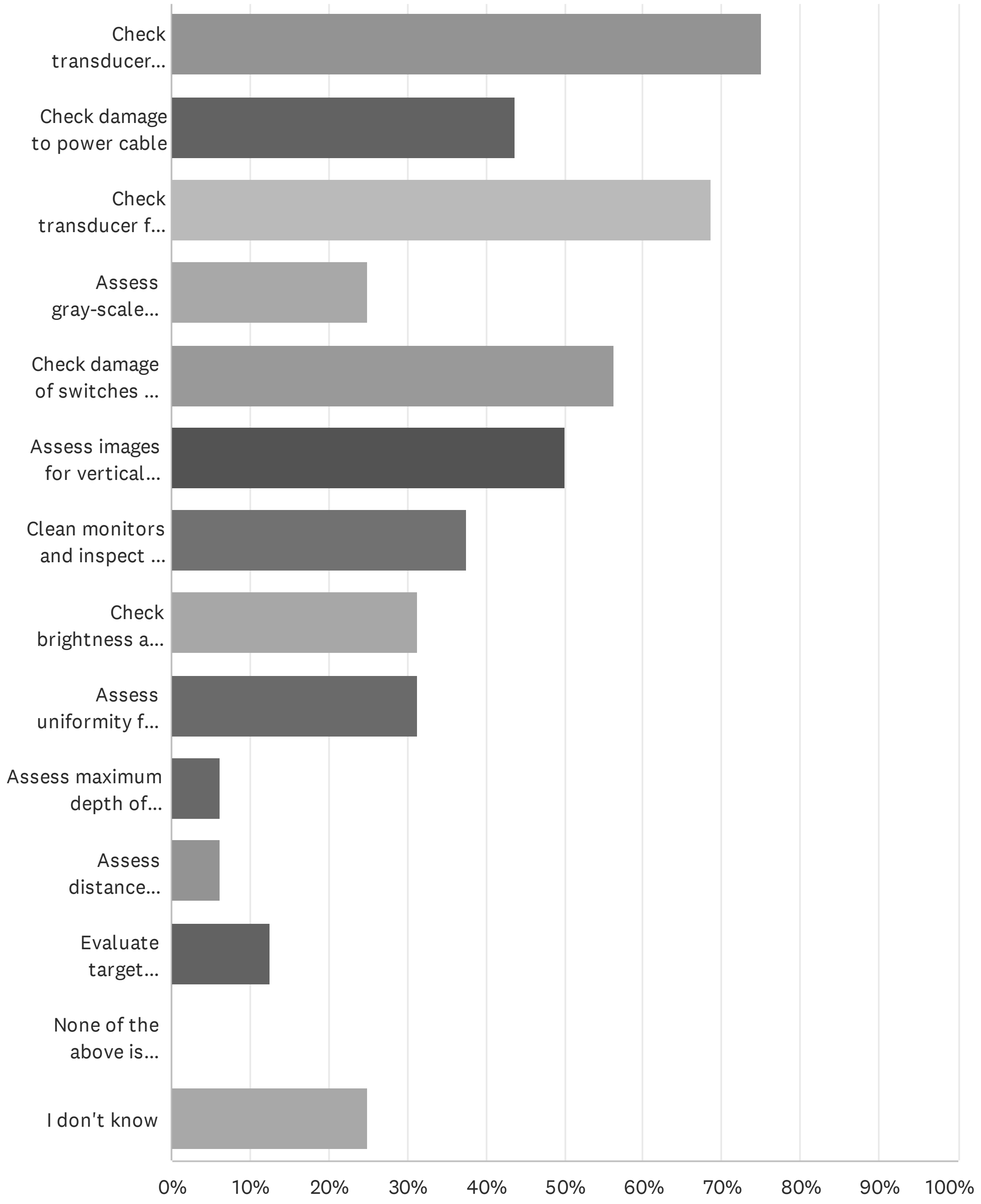

ANSWER CHOICES	RESPONSES	
Check transducer cables, housing, transmitting surfaces for damage	75.00%	12
Check damage to power cable	43.75%	7
Check transducer for cracks, separations, and discolorations	68.75%	11
Assess gray-scale settings of monitors	25.00%	4
Check damage of switches and knobs on scanner console	56.25%	9
Assess images for vertical shadows and streaks caused by dead elements within the transducer probe	50.00%	8
Clean monitors and inspect for cracks	37.50%	6
Check brightness and contrast controls of monitors	31.25%	5
Assess uniformity for each transducer*	31.25%	5
Assess maximum depth of visualisation for each transducer	6.25%	1
Assess distance measurement accuracy for each transducer	6.25%	1
Evaluate target resolution for each transducer	12.50%	2
None of the above is regularly checked	0.00%	0
I don't know	25.00%	4
Total Respondents: 16		

Q19 In your opinion - which parameters should be included in the description of experiments to ensure reproducibility.

Answered: 15 Skipped: 136

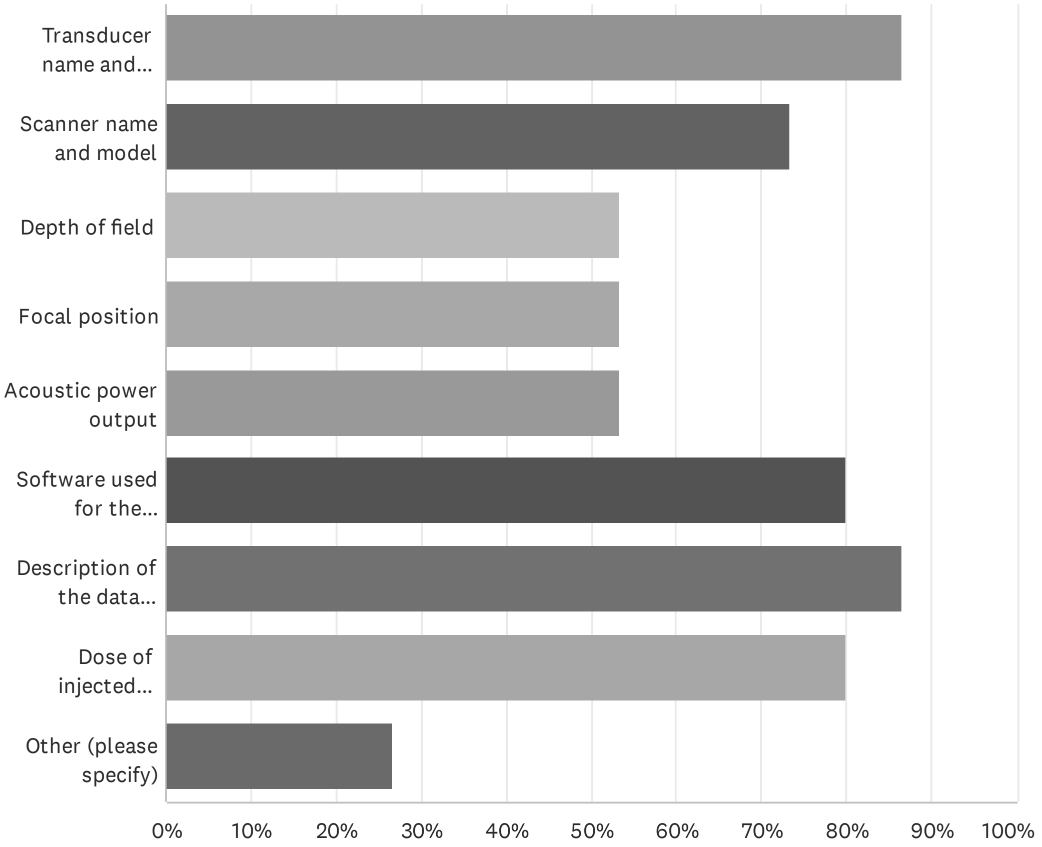

ANSWER CHOICES	RESPONSES	
Transducer name and frequency	86.67%	13
Scanner name and model	73.33%	11
Depth of field	53.33%	8
Focal position	53.33%	8
Acoustic power output	53.33%	8
Software used for the analysis	80.00%	12
Description of the data analysis	86.67%	13
Dose of injected contrast agent (if applicable)	80.00%	12
Other (please specify)	26.67%	4
Total Respondents: 15		

#	OTHER (PLEASE SPECIFY)	DATE
1	Frames per second in motion analysis	1/27/2022 2:08 AM
2	cardiac frequency, animal body temperature, breath frequency	12/13/2021 1:52 PM
3	(I'd need to check with the people doing this, I'm just the PI who has not done acquisition)	12/10/2021 2:30 PM

4	I don't know	12/10/2021 2:00 PM

Q20 Are you working with/using preclinical Optical Device(s)?

Answered: 132 Skipped: 19

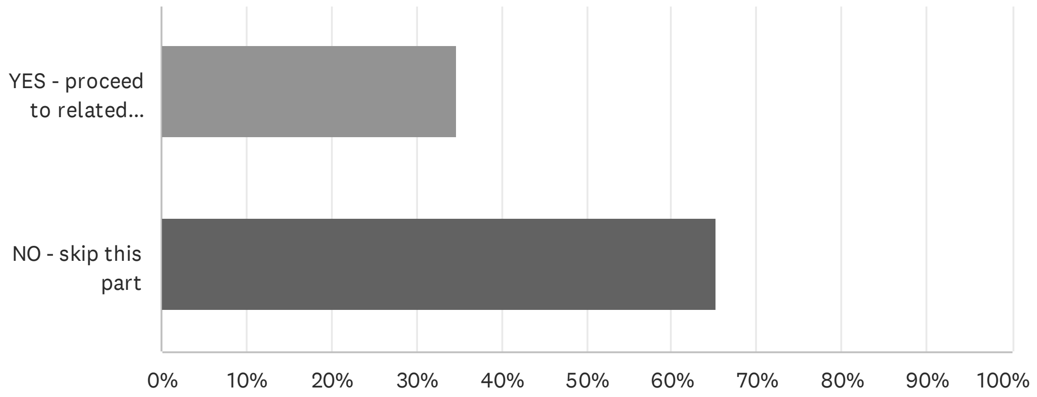

ANSWER CHOICES	RESPONSES	
YES - proceed to related questions	34.85%	46
NO - skip this part	65.15%	86
Total Respondents: 132		

Q21 What kind of imaging experiments do you perform?(tick all relevant items)

Answered: 44 Skipped: 107

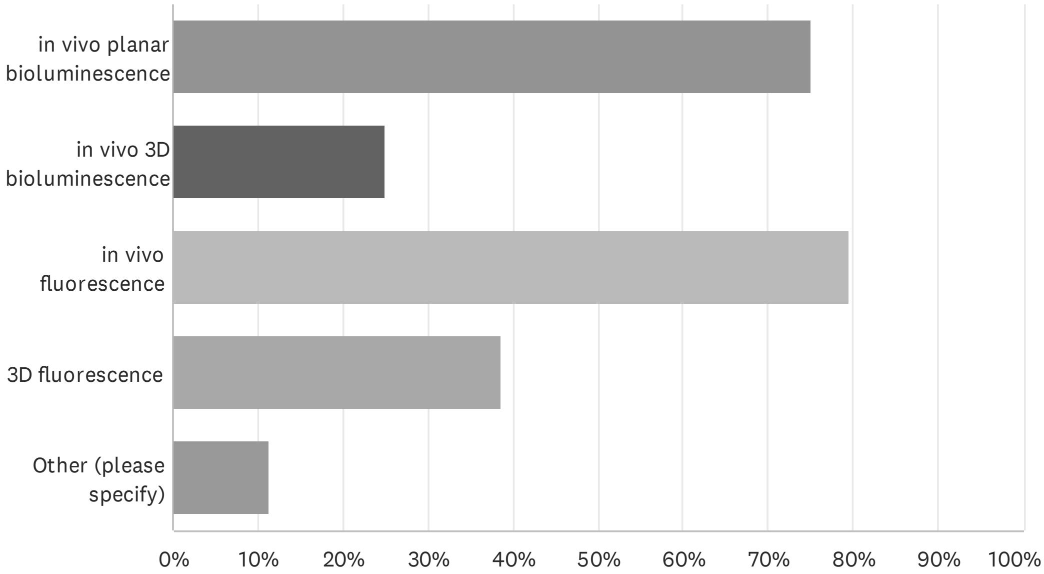

ANSWER CHOICES	RESPONSES	
in vivo planar bioluminescence	75.00%	33
in vivo 3D bioluminescence	25.00%	11
in vivo fluorescence	79.55%	35
3D fluorescence	38.64%	17
Other (please specify)	11.36%	5
Total Respondents: 44		

#	OTHER (PLEASE SPECIFY)	DATE
1	OCT, OPT	1/18/2022 11:21 AM
2	photoacoustic imaging	12/13/2021 4:54 PM
3	optoacoustic	12/10/2021 4:47 PM
4	intravital microscopy (FCFM)	12/10/2021 2:31 PM
5	Cherenkove effect	12/10/2021 1:54 PM

Q22 What is the frequency of regular scanner-maintenance by the manufacturer?'

Answered: 43 Skipped: 108

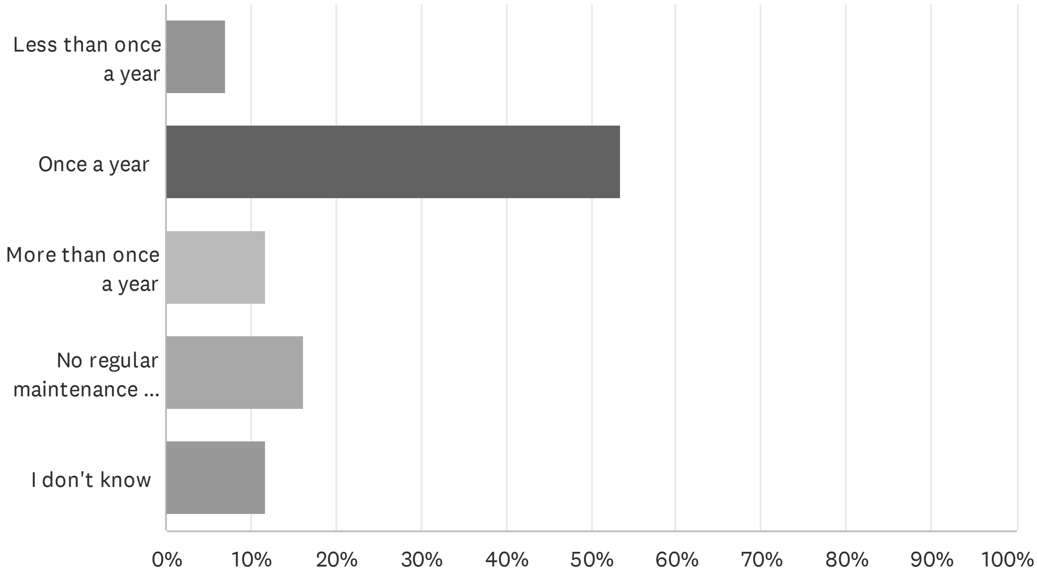

ANSWER CHOICES	RESPONSES	
Less than once a year	6.98%	3
Once a year	53.49%	23
More than once a year	11.63%	5
No regular maintenance by the manfacturer	16.28%	7
I don't know	11.63%	5
TOTAL		43

Q23 How ofter is the scanner calibrated?

Answered: 43 Skipped: 108

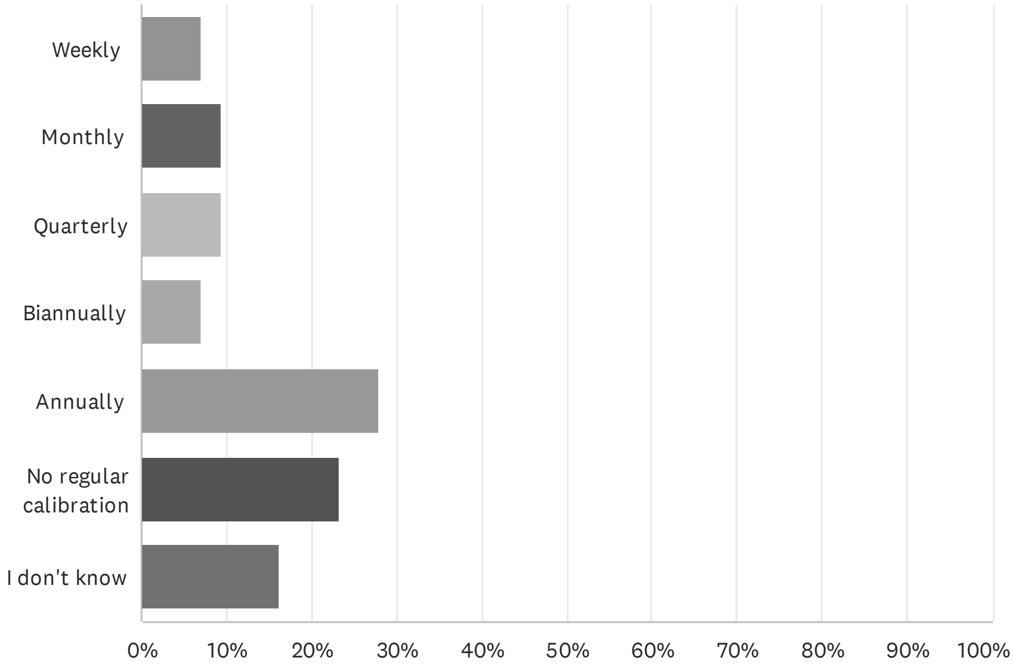

ANSWER CHOICES	RESPONSES	
Weekly	6.98%	3
Monthly	9.30%	4
Quarterly	9.30%	4
Biannually	6.98%	3
Annually	27.91%	12
No regular calibration	23.26%	10
I don't know	16.28%	7
TOTAL		43

Q24 If available - would you use a fluorescent standard for relative quantitative data analysis

Answered: 43 Skipped: 108

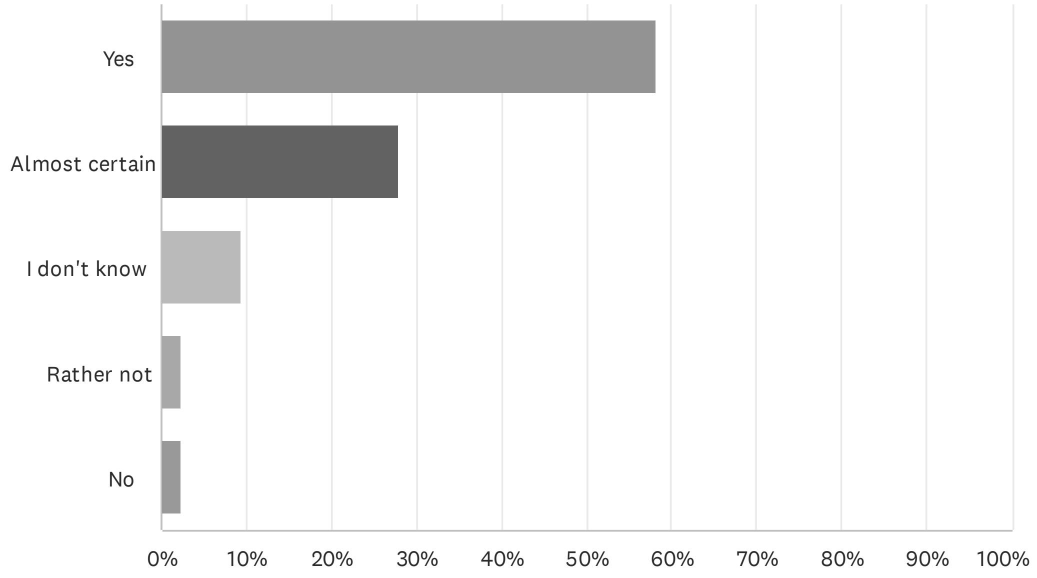

ANSWER CHOICES	RESPONSES	
Yes	58.14%	25
Almost certain	27.91%	12
I don't know	9.30%	4
Rather not	2.33%	1
No	2.33%	1
TOTAL		43

Q25 If available - would you use a luminescent standard for relative quantitative data analysis

Answered: 42 Skipped: 109

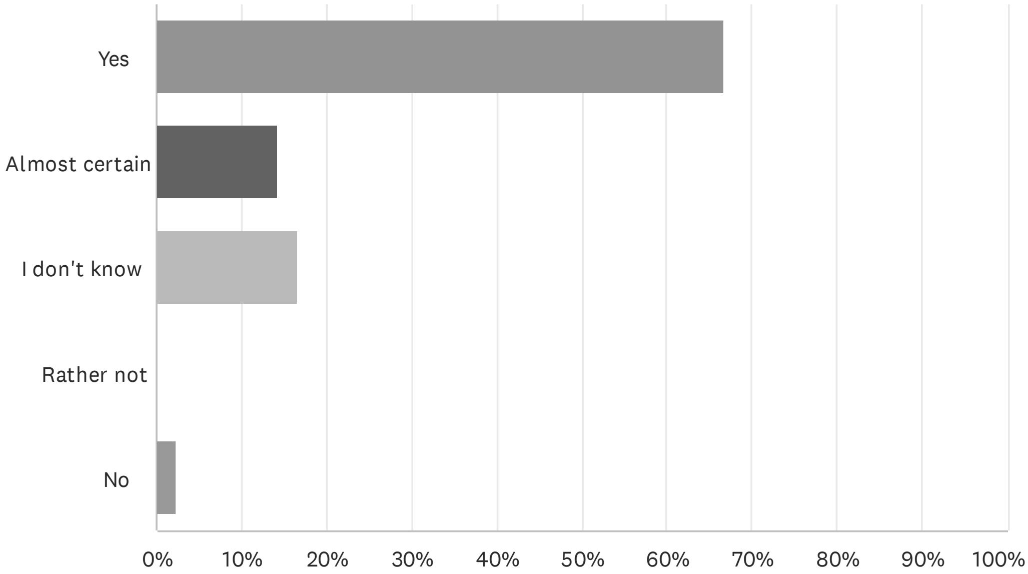

ANSWER CHOICES	RESPONSES	
Yes	66.67%	28
Almost certain	14.29%	6
I don't know	16.67%	7
Rather not	0.00%	0
No	2.38%	1
TOTAL		42

Q26 If the answer above positive - what would you be willing to pay for such a tool?

Answered: 39 Skipped: 112

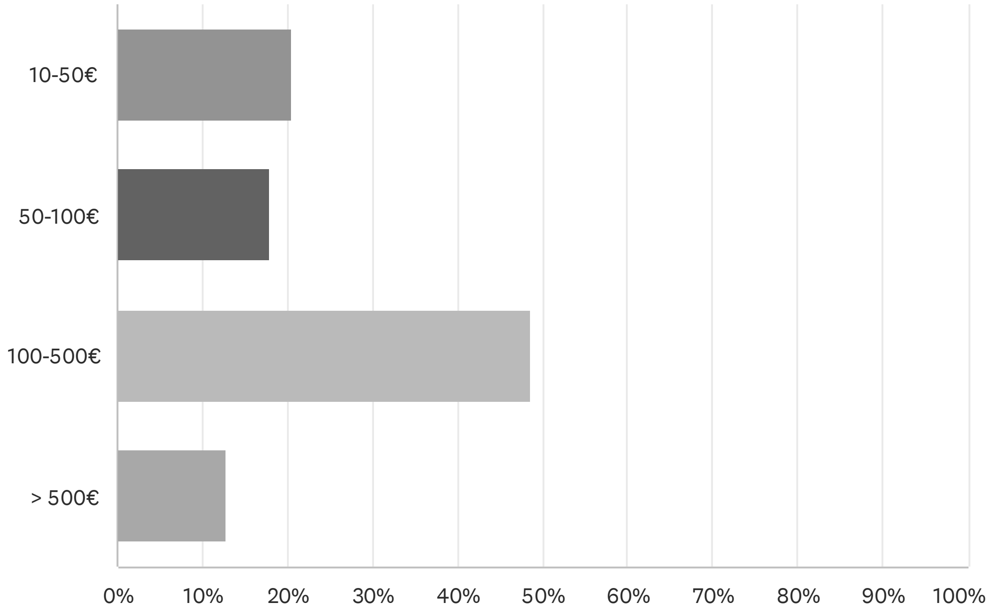

ANSWER CHOICES	RESPONSES	
10-50€	20.51%	8
50-100€	17.95%	7
100-500€	48.72%	19
> 500€	12.82%	5
TOTAL		39

Q27 Would you report photon/fluxes in comparing data sets?

Answered: 43 Skipped: 108

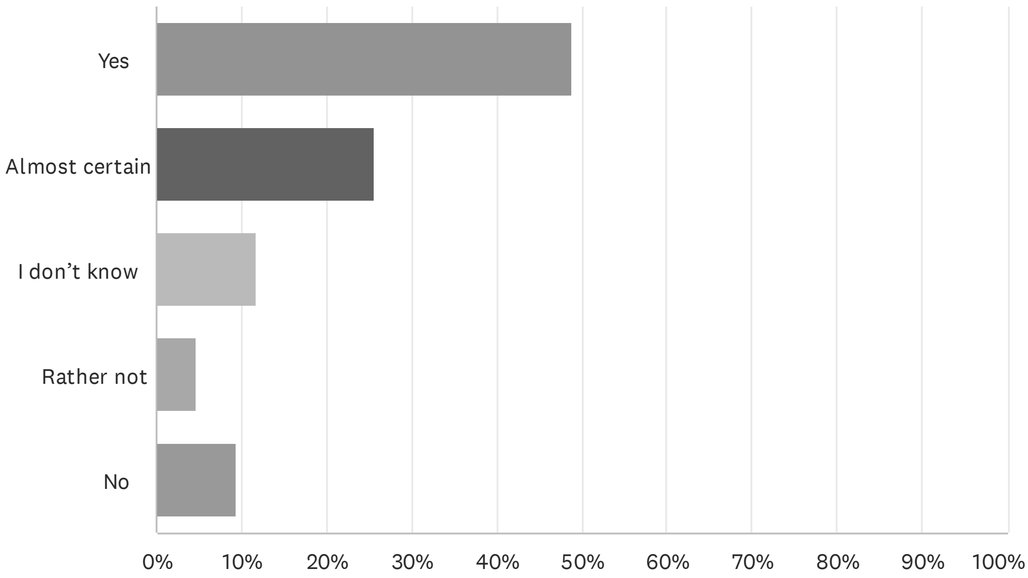

ANSWER CHOICES	RESPONSES	
Yes	48.84%	21
Almost certain	25.58%	11
I don't know	11.63%	5
Rather not	4.65%	2
No	9.30%	4
TOTAL		43

Q28 Would you report relative signals to compare data sets (e.g. fold differences in photon fluxes or average radiance)?

Answered: 43 Skipped: 108

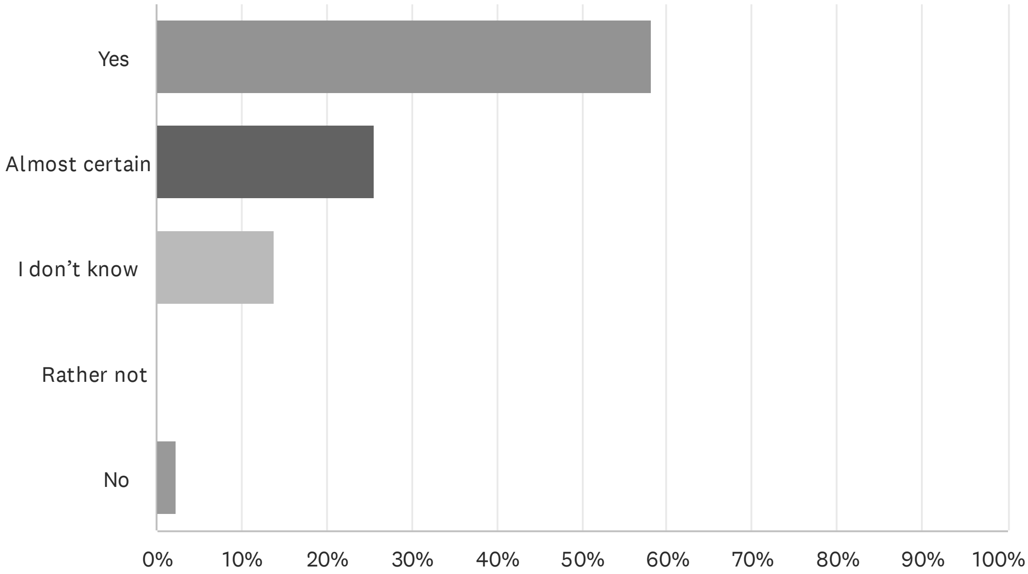

ANSWER CHOICES	RESPONSES	
Yes	58.14%	25
Almost certain	25.58%	11
I don't know	13.95%	6
Rather not	0.00%	0
No	2.33%	1
TOTAL		43

Q29 In your opinion - which parameters should be included in the description of experiments to ensure reproducibility.(tick all relevant items)

Answered: 41 Skipped: 110

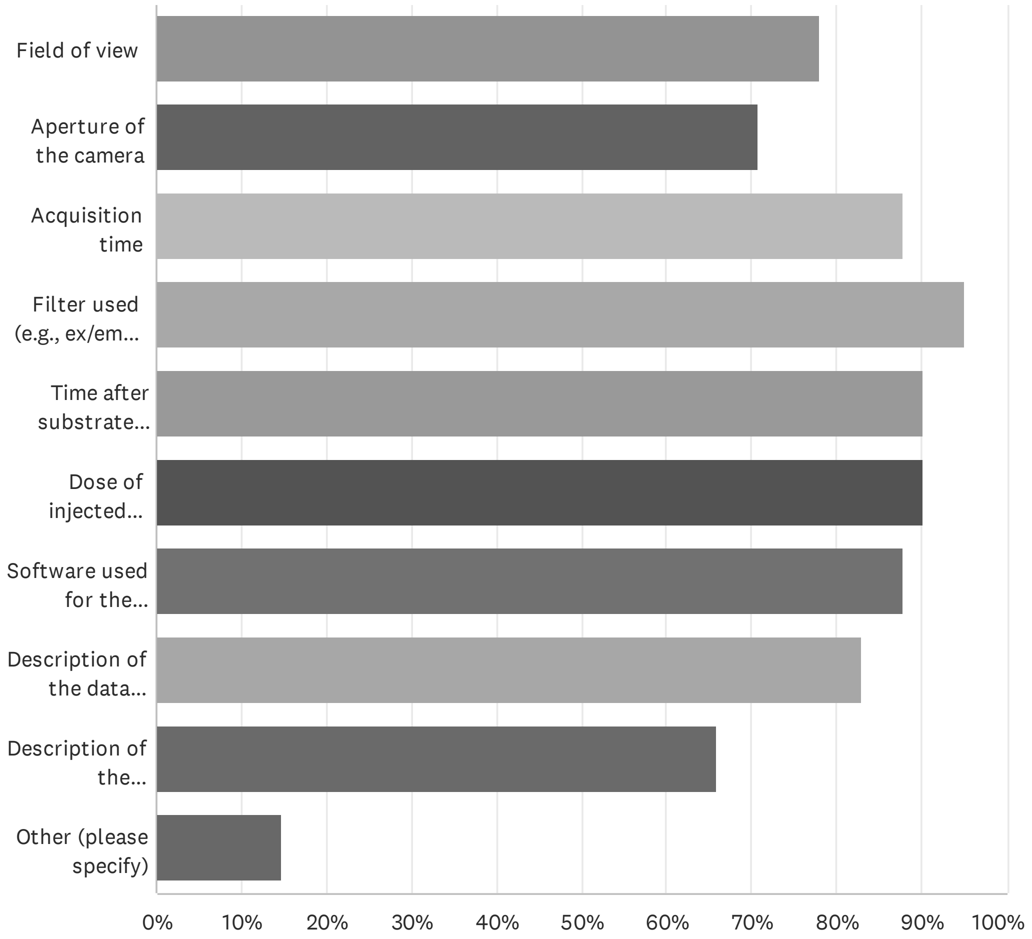

ANSWER CHOICES	RESPONSES	
Field of view	78.05%	32
Aperture of the camera	70.73%	29
Acquisition time	87.80%	36
Filter used (e.g., ex/em, no filters)	95.12%	39
Time after substrate injection for bioluminescence	90.24%	37
Dose of injected substrate	90.24%	37
Software used for the analysis	87.80%	36
Description of the data analysis	82.93%	34
Description of the reconstruction parameters in 3D analysis	65.85%	27
Other (please specify)	14.63%	6
Total Respondents: 41		

#	OTHER (PLEASE SPECIFY)	DATE
1	Time for peak signal in BLI studies	1/27/2022 2:10 AM
2	Type of imaging objective, excitation sources, type of detectors used.	1/26/2022 9:17 AM
3	system does not allow change of acquisition time (LiCor Pearl)	12/12/2021 5:41 PM
4	script for analysis, camera type, lenses type, PSF	12/10/2021 7:08 PM
5	all of it	12/10/2021 2:31 PM
6	I don't know	12/10/2021 2:01 PM

Q30 Are you working with/using preclinical SPECT Device(s)?

Answered: 132 Skipped: 19

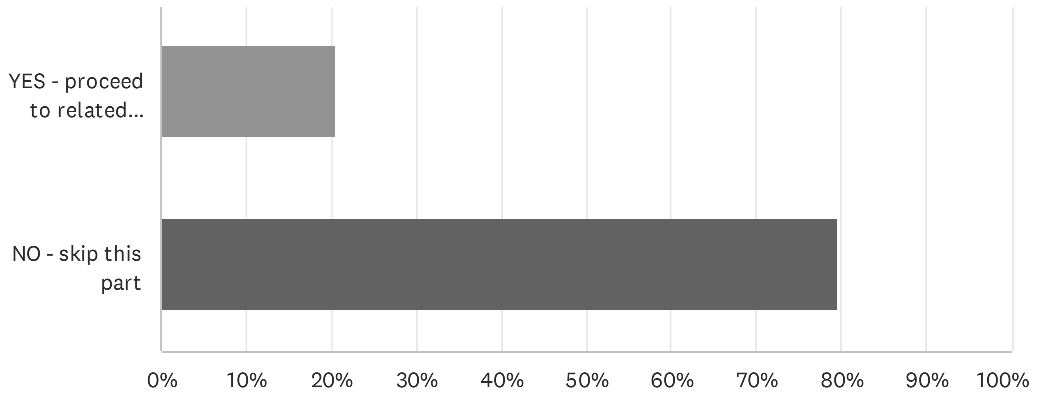

ANSWER CHOICES	RESPONSES	
YES - proceed to related questions	20.45%	27
NO - skip this part	79.55%	105
Total Respondents: 132		

Q31 What kind of imaging experiments do you perform?(tick all relevant items)

Answered: 26 Skipped: 125

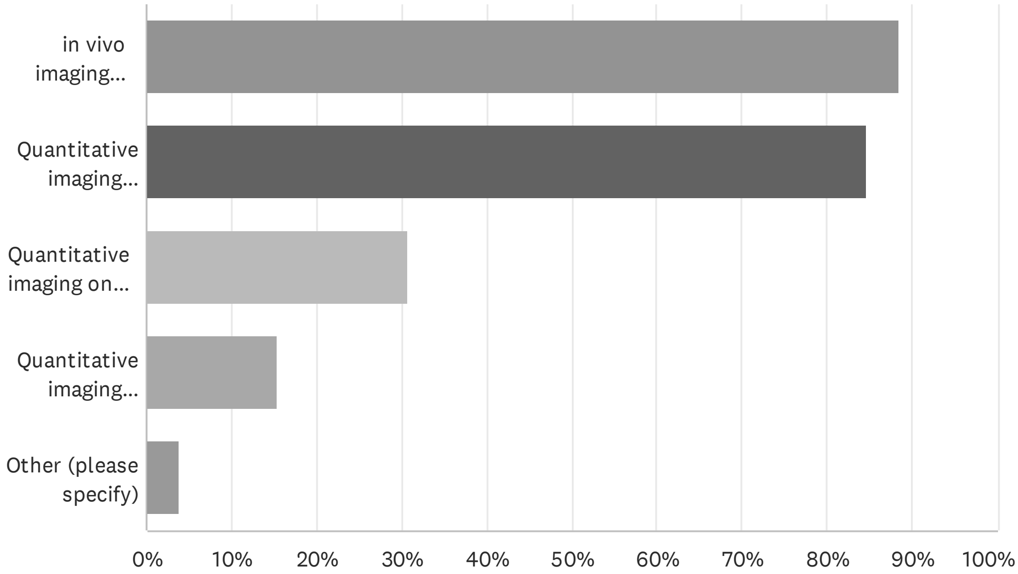

ANSWER CHOICES	RESPONSES	
in vivo imaging experiments	88.46%	23
Quantitative imaging experiments in vivo	84.62%	22
Quantitative imaging on cadaver material or ex vivo	30.77%	8
Quantitative imaging experiments in vitro	15.38%	4
Other (please specify)	3.85%	1
Total Respondents: 26		

#	OTHER (PLEASE SPECIFY)	DATE
1	phantom studies	12/13/2021 11:50 AM

Q32 What is the frequency of regular scanner-maintenance by the manufacturer?'

Answered: 26 Skipped: 125

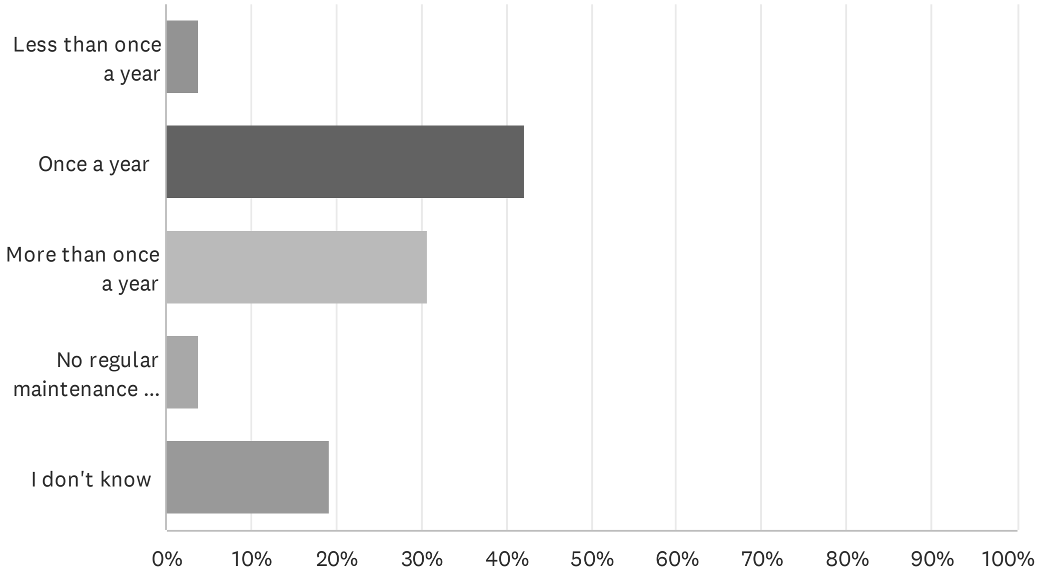

ANSWER CHOICES	RESPONSES	
Less than once a year	3.85%	1
Once a year	42.31%	11
More than once a year	30.77%	8
No regular maintenance by the manfacturer	3.85%	1
I don't know	19.23%	5
TOTAL		26

Q33 What is the frequency of quantitative scanner calibration?

Answered: 26 Skipped: 125

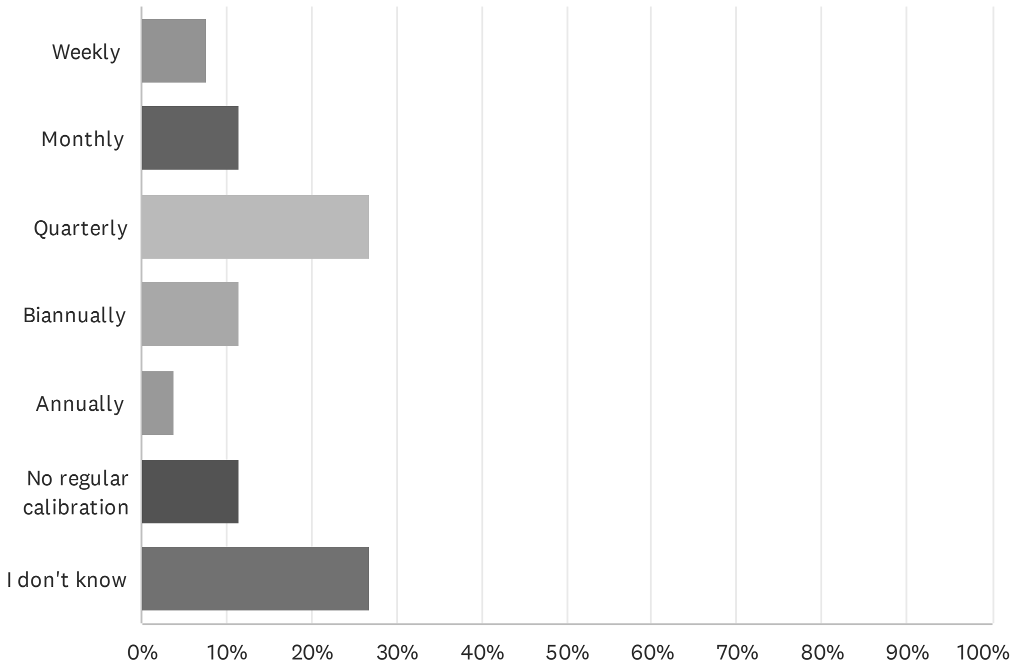

ANSWER CHOICES	RESPONSES	
Weekly	7.69%	2
Monthly	11.54%	3
Quarterly	26.92%	7
Biannually	11.54%	3
Annually	3.85%	1
No regular calibration	11.54%	3
I don't know	26.92%	7
TOTAL		26

Q34 How often do you perform cross-calibration between dose calibrator and SPECT scanner

Answered: 26 Skipped: 125

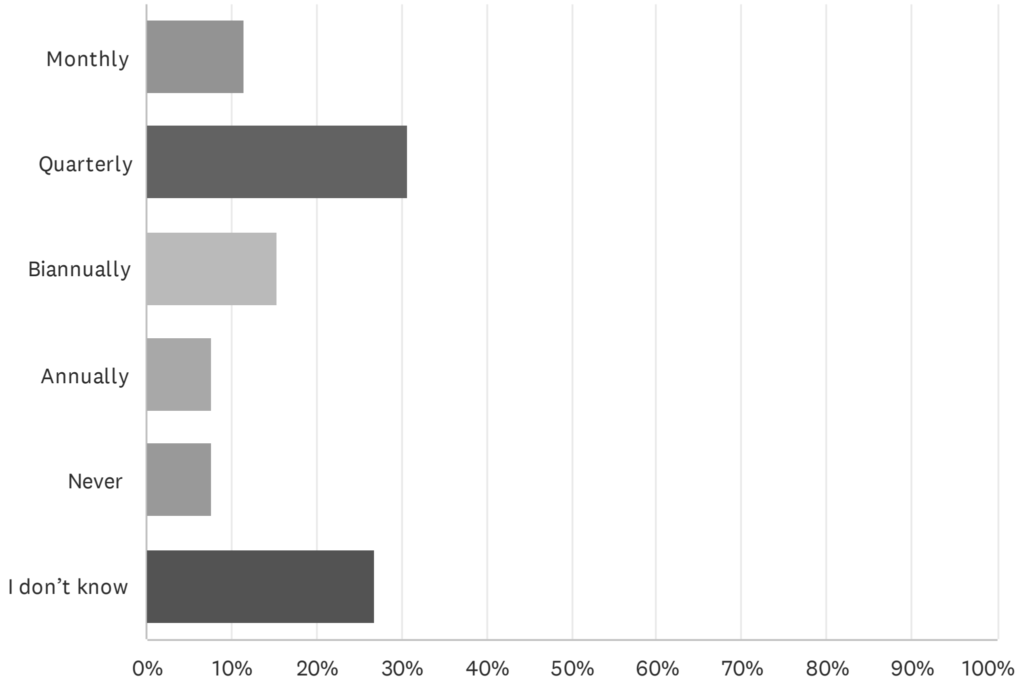

ANSWER CHOICES	RESPONSES	
Monthly	11.54%	3
Quarterly	30.77%	8
Biannually	15.38%	4
Annually	7.69%	2
Never	7.69%	2
I don’t know	26.92%	7
TOTAL		26

Q35 Does cross-calibration also include the gamma and/or well counter? only applicable if cross-calibration is performed

Answered: 24 Skipped: 127

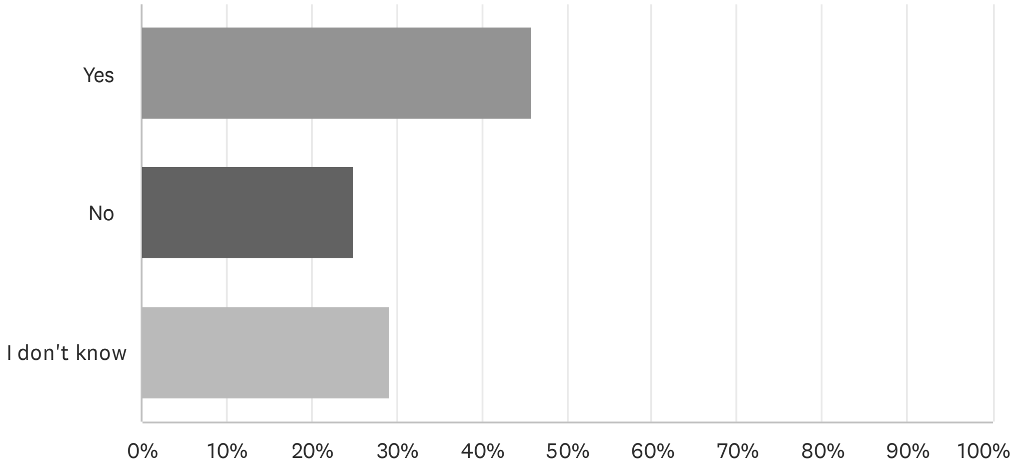

ANSWER CHOICES	RESPONSES	
Yes	45.83%	11
No	25.00%	6
I don't know	29.17%	7
TOTAL		24

Q36 How frequently do you check photopeak drift?

Answered: 25 Skipped: 126

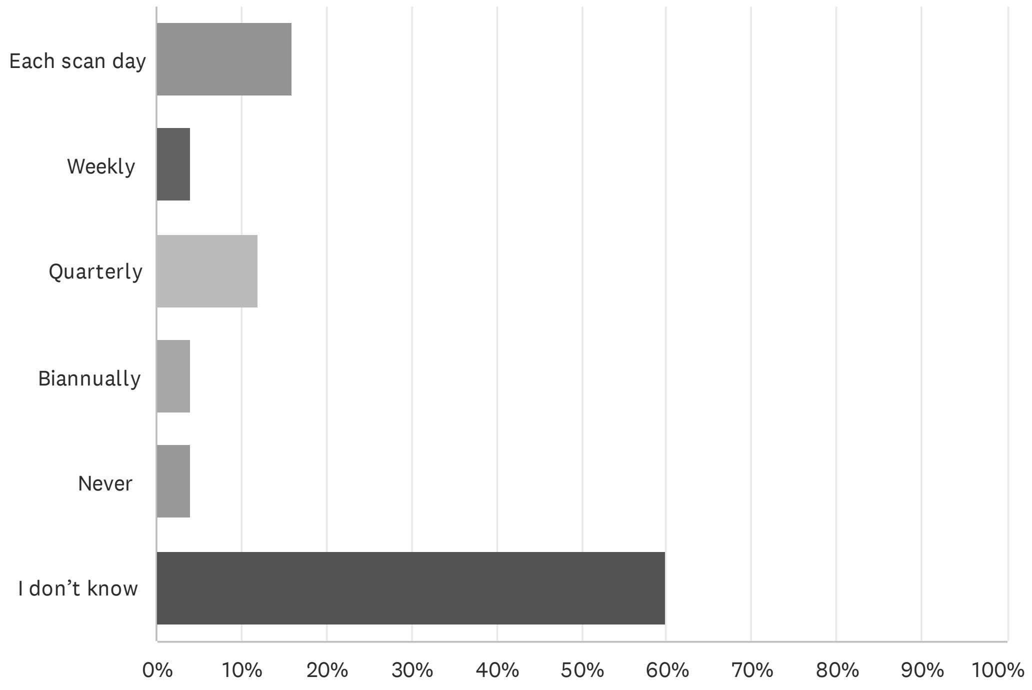

ANSWER CHOICES	RESPONSES	
Each scan day	16.00%	4
Weekly	4.00%	1
Quarterly	12.00%	3
Biannually	4.00%	1
Never	4.00%	1
I don't know	60.00%	15
Total Respondents: 25		

Q37 How frequently do you perform uniformity tests?

Answered: 25 Skipped: 126

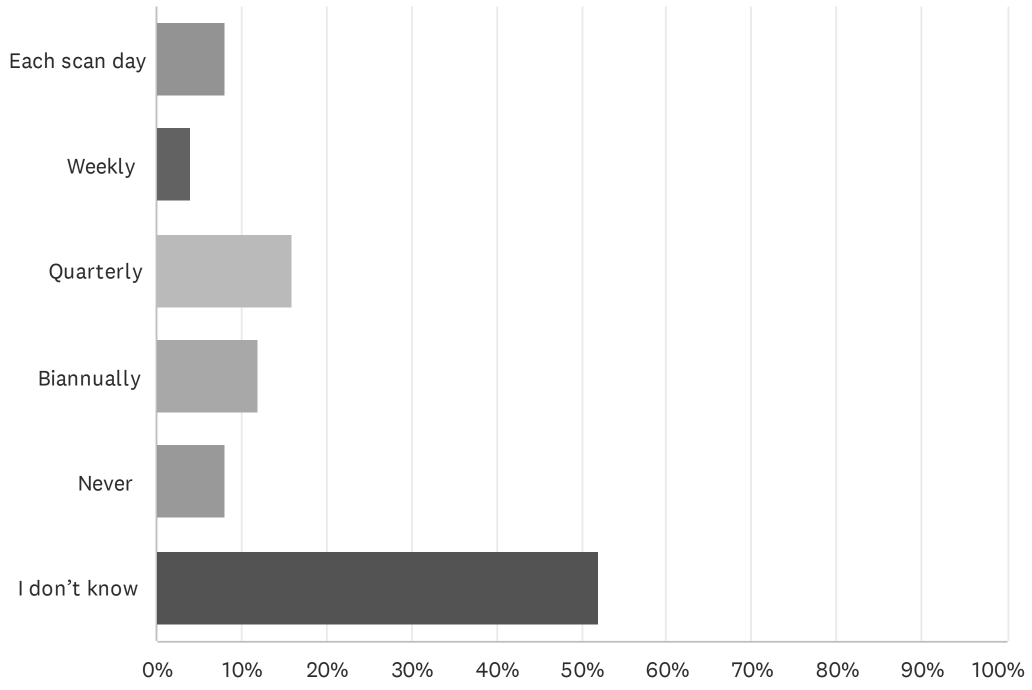

ANSWER CHOICES	RESPONSES	
Each scan day	8.00%	2
Weekly	4.00%	1
Quarterly	16.00%	4
Biannually	12.00%	3
Never	8.00%	2
I don't know	52.00%	13
TOTAL		25

Q38 How frequently do you check collimator and detector stability?

Answered: 25 Skipped: 126

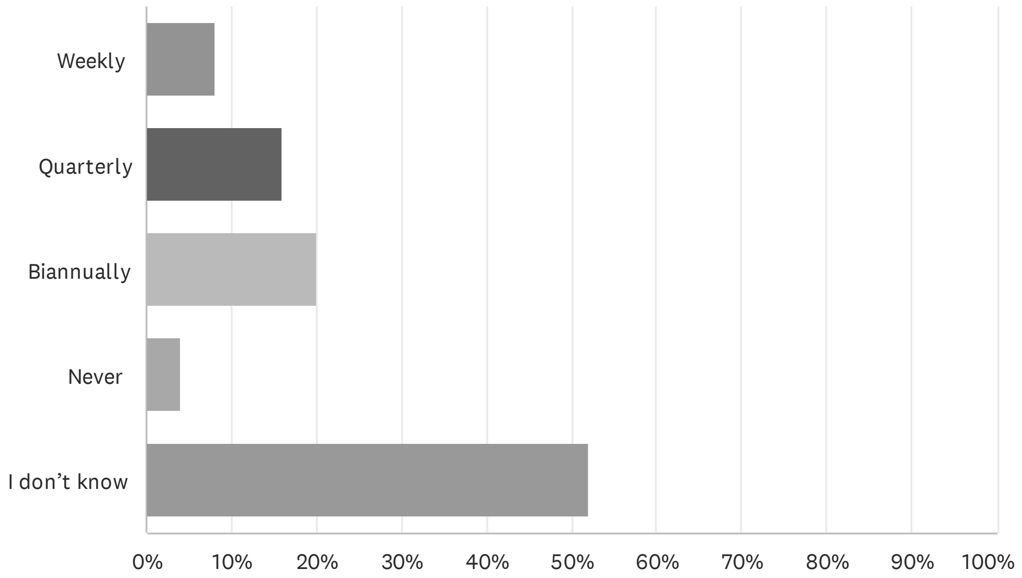

ANSWER CHOICES	RESPONSES	
Weekly	8.00%	2
Quarterly	16.00%	4
Biannually	20.00%	5
Never	4.00%	1
I don’t know	52.00%	13
TOTAL		25

Q39 How frequently do you perform multimodal registration checks on your system?

Answered: 25 Skipped: 126

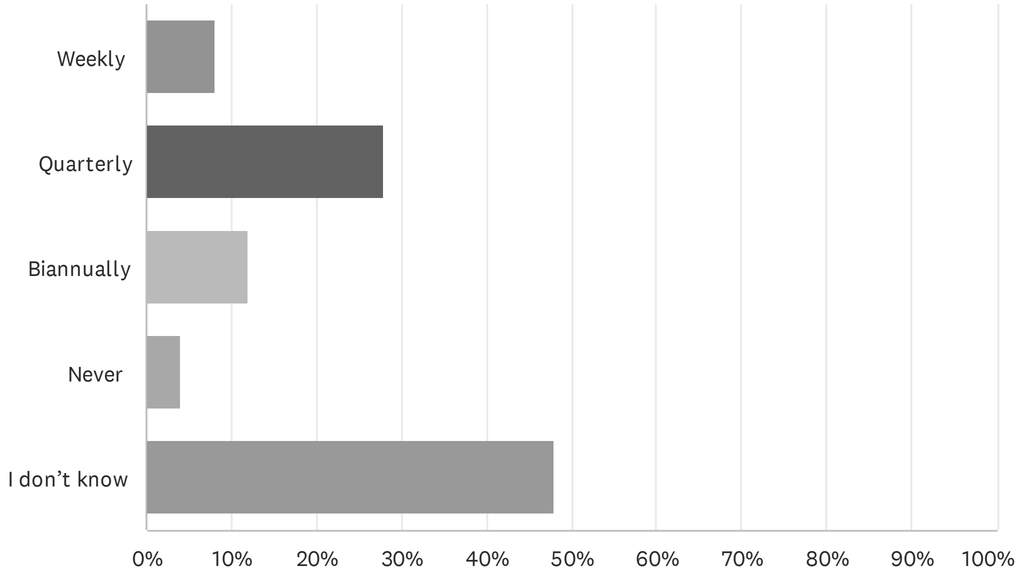

ANSWER CHOICES	RESPONSES	
Weekly	8.00%	2
Quarterly	28.00%	7
Biannually	12.00%	3
Never	4.00%	1
I don’t know	48.00%	12
TOTAL		25

Q40 Do you use and/or provide methods for reconstruction?

Answered: 25 Skipped: 126

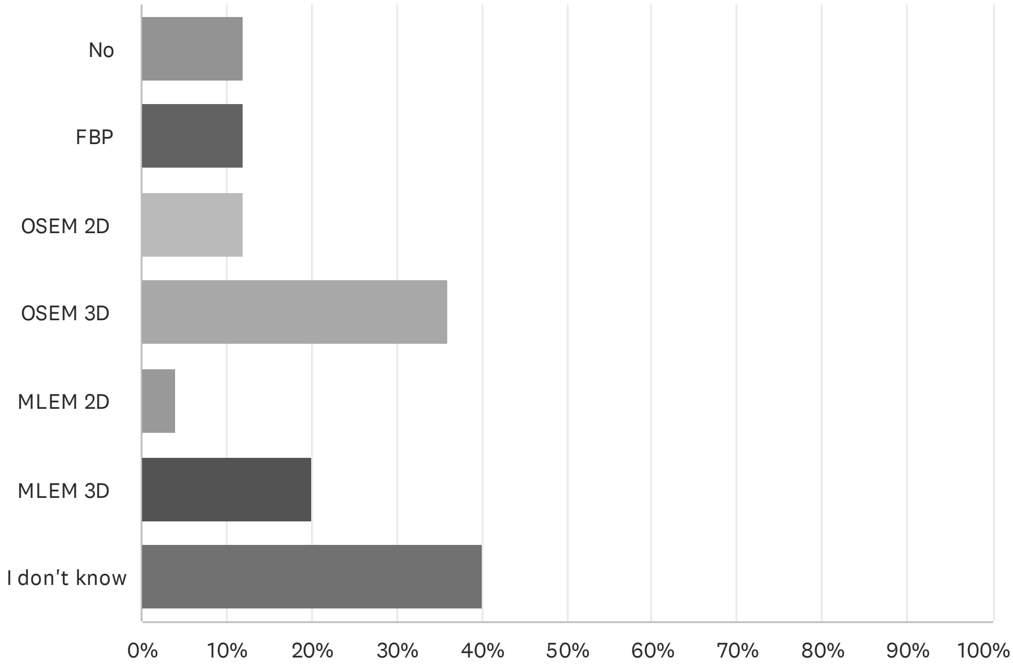

ANSWER CHOICES	RESPONSES	
No	12.00%	3
FBP	12.00%	3
OSEM 2D	12.00%	3
OSEM 3D	36.00%	9
MLEM 2D	4.00%	1
MLEM 3D	20.00%	5
I don't know	40.00%	10
Total Respondents: 25		

Q41 Are you working with/using preclinical PET Device(s)?

Answered: 132 Skipped: 19

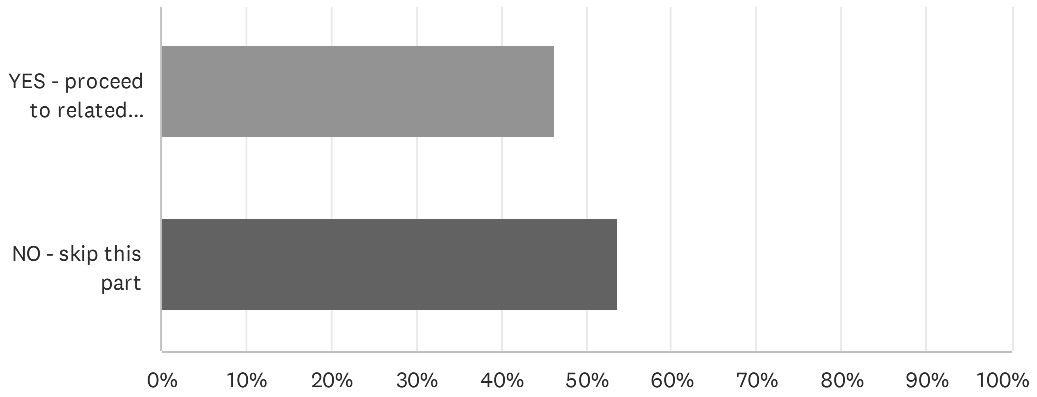

ANSWER CHOICES	RESPONSES	
YES - proceed to related questions	46.21%	61
NO - skip this part	53.79%	71
Total Respondents: 132		

Q42 What is the frequency of regular scanner-maintenance by the manufacturer?'

Answered: 57 Skipped: 94

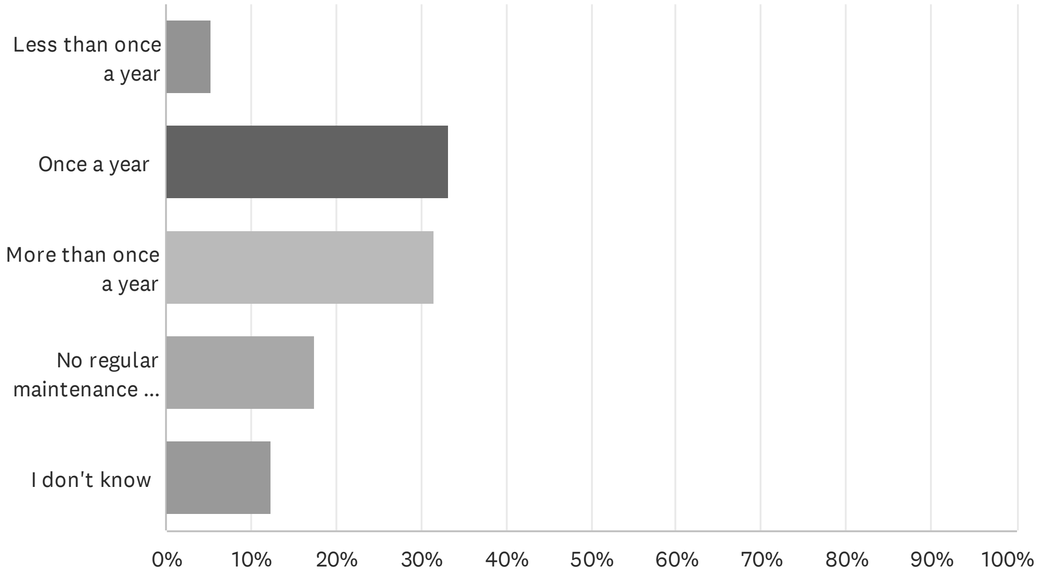

ANSWER CHOICES	RESPONSES	
Less than once a year	5.26%	3
Once a year	33.33%	19
More than once a year	31.58%	18
No regular maintenance by the manfacturer	17.54%	10
I don't know	12.28%	7
TOTAL		57

Q43 How ofte is the scanner calibrated?

Answered: 57 Skipped: 94

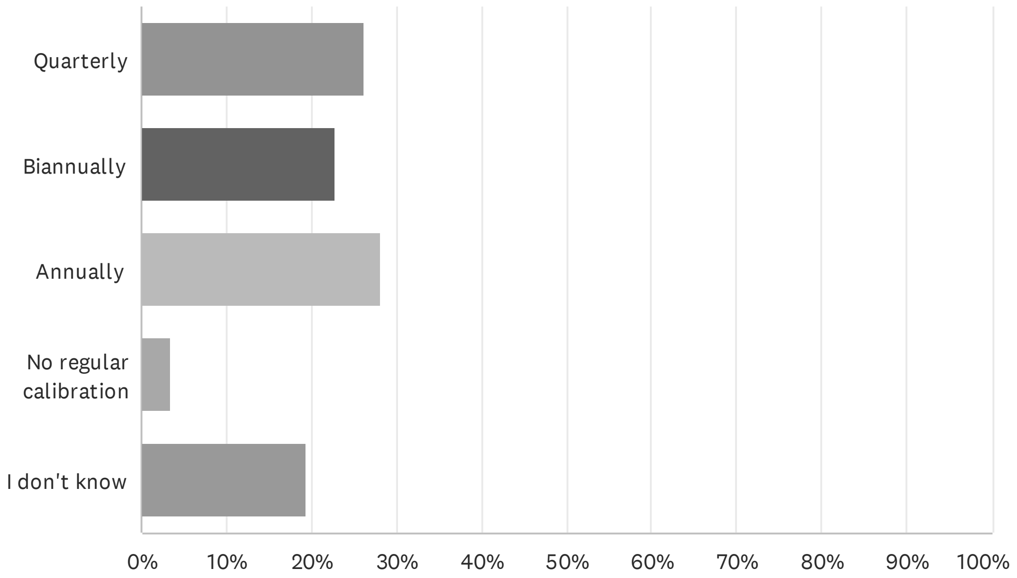

ANSWER CHOICES	RESPONSES	
Quarterly	26.32%	15
Biannually	22.81%	13
Annually	28.07%	16
No regular calibration	3.51%	2
I don't know	19.30%	11
TOTAL		57

Q44 Does this calibration include check of...(tick all items of relevance)

Answered: 56 Skipped: 95

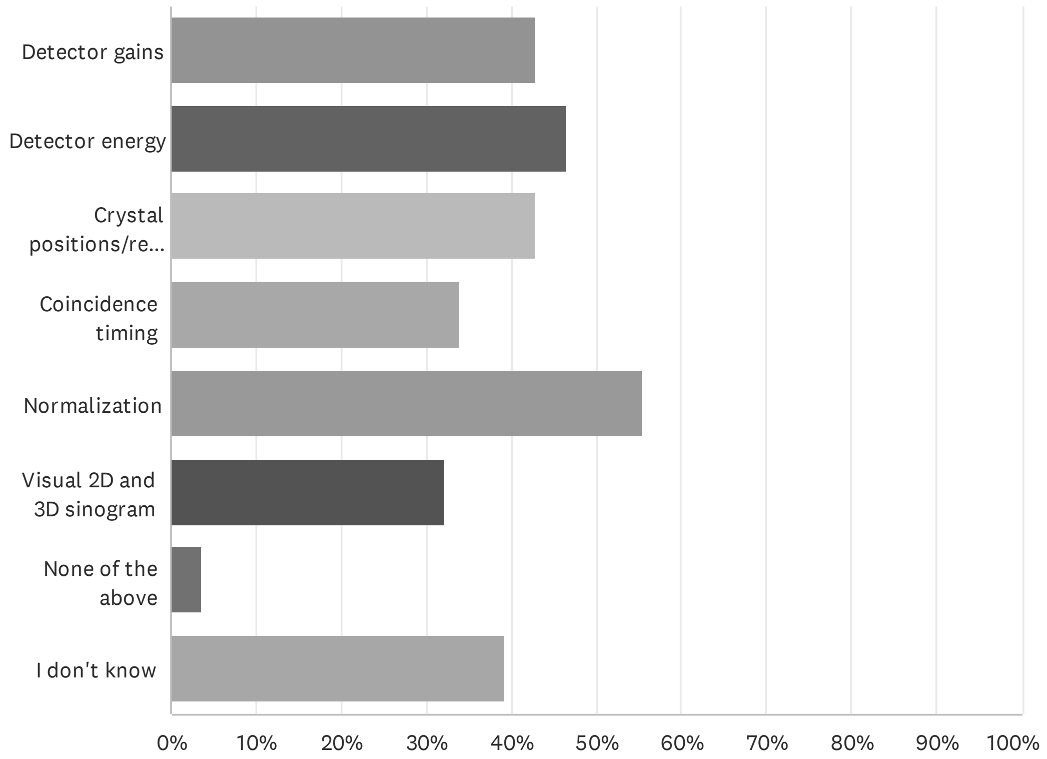

ANSWER CHOICES	RESPONSES	
Detector gains	42.86%	24
Detector energy	46.43%	26
Crystal positions/read-out (crystal maps)	42.86%	24
Coincidence timing	33.93%	19
Normalization	55.36%	31
Visual 2D and 3D sinogram	32.14%	18
None of the above	3.57%	2
I don't know	39.29%	22
Total Respondents: 56		

Q45 Are cross-calibrations performed between the calibrator, PET scanner and/or the gamma counter?

Answered: 56 Skipped: 95

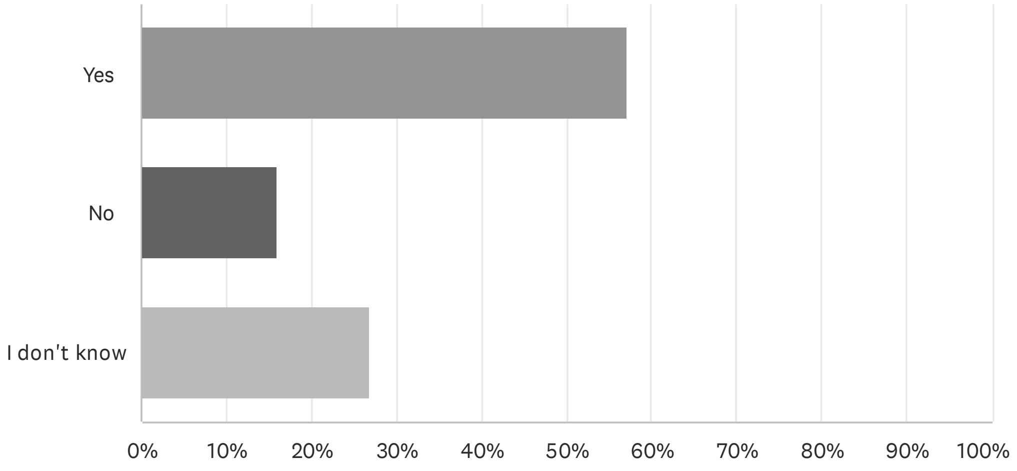

ANSWER CHOICES	RESPONSES	
Yes	57.14%	32
No	16.07%	9
I don't know	26.79%	15
TOTAL		56

Q46 Is regular Quality Control performed:(tick all relevant items)

Answered: 56 Skipped: 95

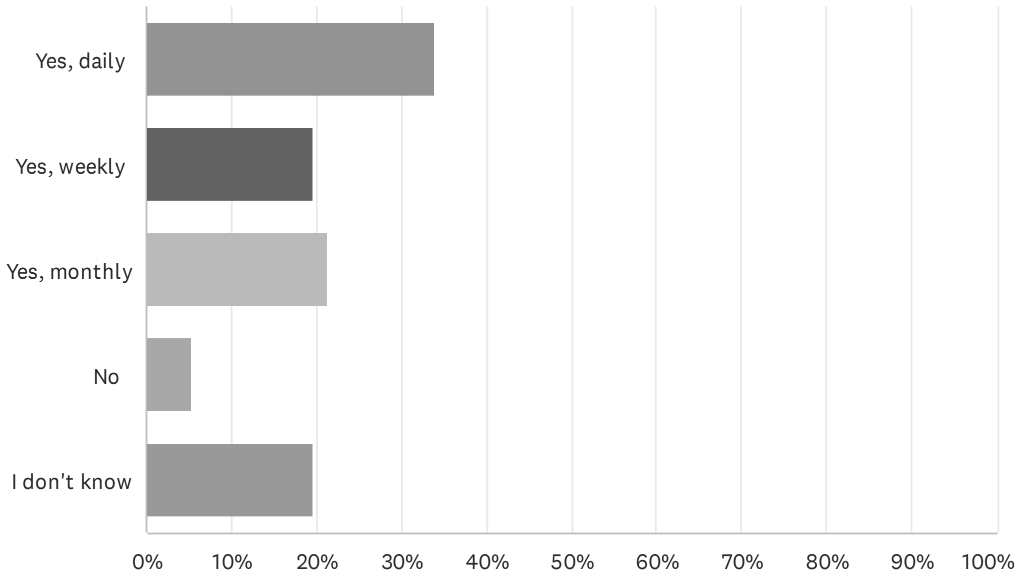

ANSWER CHOICES	RESPONSES	
Yes, daily	33.93%	19
Yes, weekly	19.64%	11
Yes, monthly	21.43%	12
No	5.36%	3
I don't know	19.64%	11
TOTAL		56

Q47 Does regular Quality Control include evaluating the scanner towards:(tick all relevant items)

Answered: 54 Skipped: 97

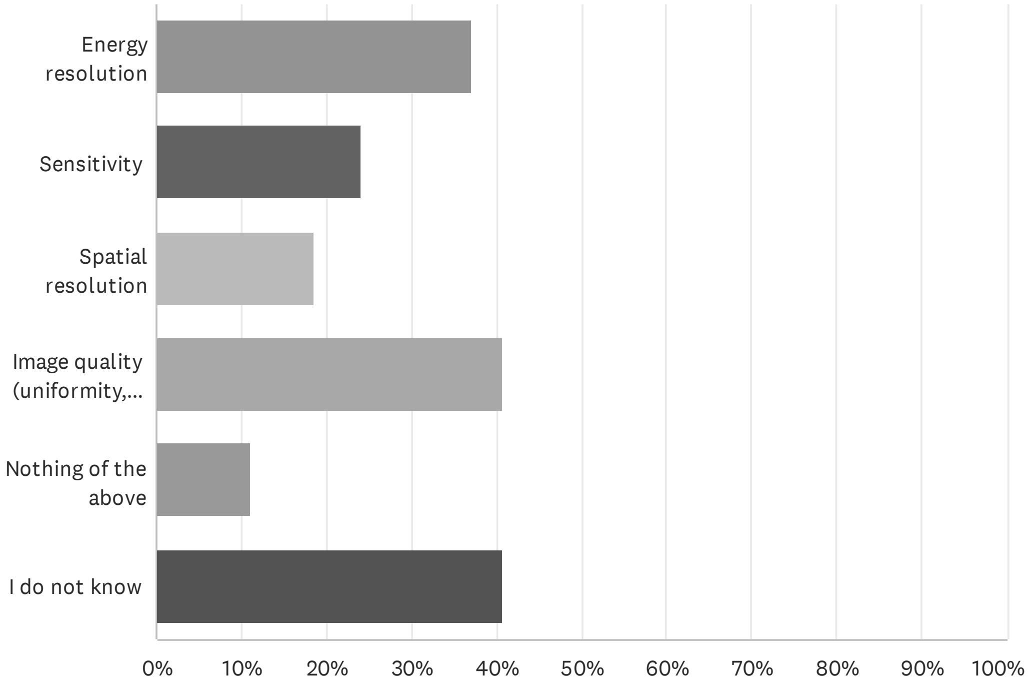

ANSWER CHOICES	RESPONSES	
Energy resolution	37.04%	20
Sensitivity	24.07%	13
Spatial resolution	18.52%	10
Image quality (uniformity, recovery coefficients, spill-out ratios)	40.74%	22
Nothing of the above	11.11%	6
I do not know	40.74%	22
Total Respondents: 54		

Q48 On scan-days: Do you perform scanner-checks towards:

Answered: 55 Skipped: 96

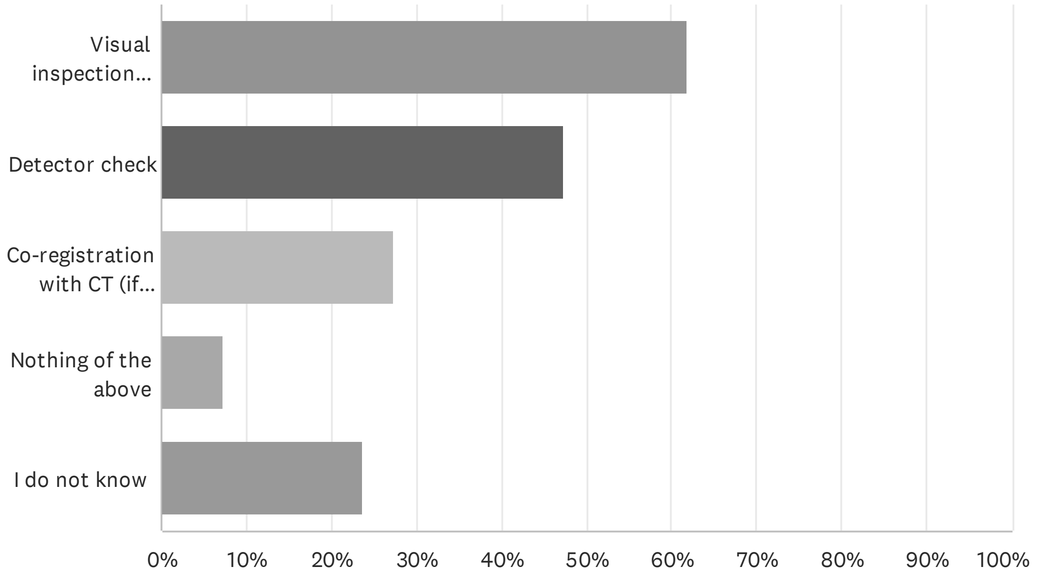

ANSWER CHOICES	RESPONSES	
Visual inspection (artefacts)	61.82%	34
Detector check	47.27%	26
Co-registration with CT (if applicable)	27.27%	15
Nothing of the above	7.27%	4
I do not know	23.64%	13
Total Respondents: 55		

Q49 Are there standard routine preclinical reconstruction protocols in place/recommended in your institute?

Answered: 54 Skipped: 97

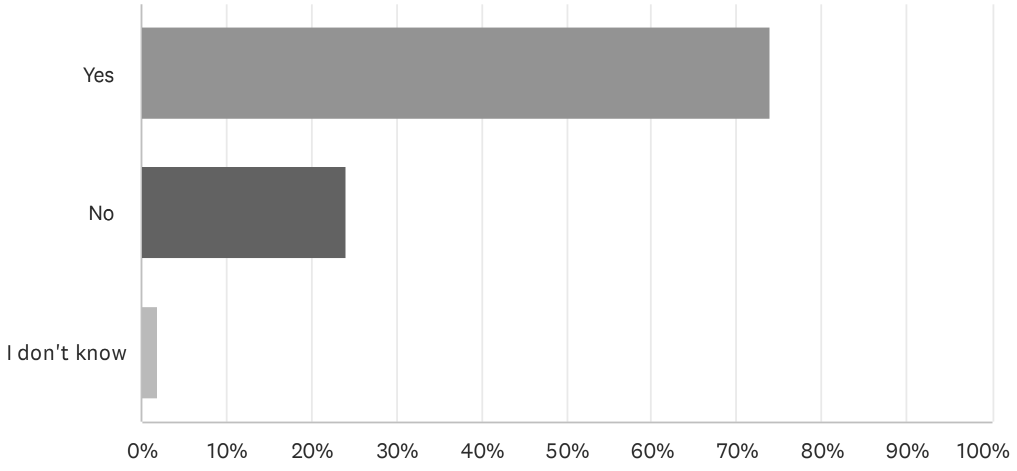

ANSWER CHOICES	RESPONSES	
Yes	74.07%	40
No	24.07%	13
I don't know	1.85%	1
TOTAL		54

Q50 Do you use and/or provide methods for reconstruction?

Answered: 55 Skipped: 96

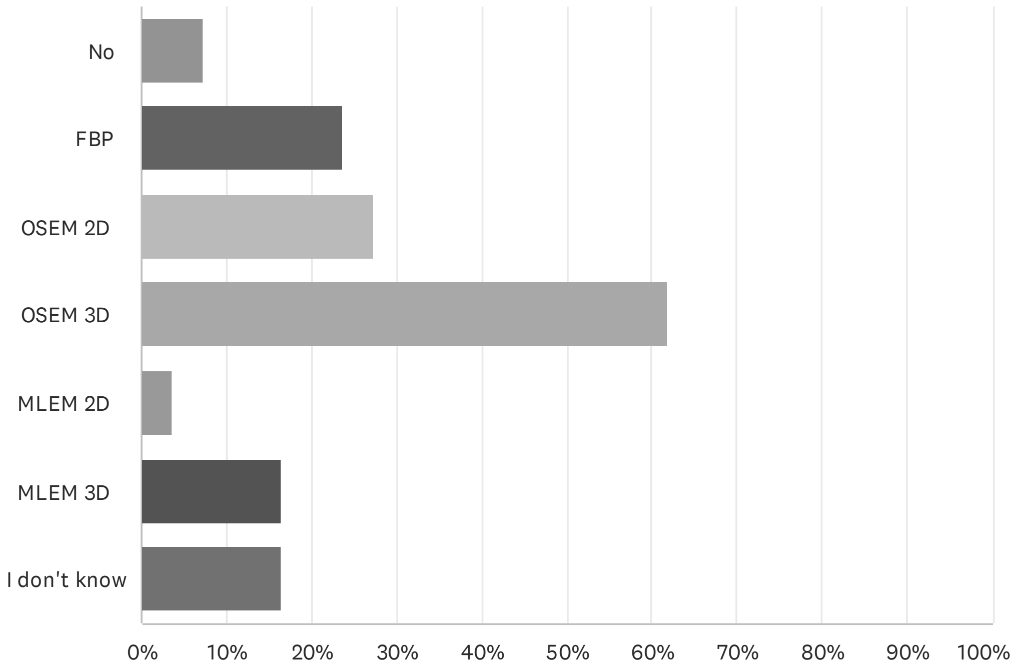

ANSWER CHOICES	RESPONSES	
No	7.27%	4
FBP	23.64%	13
OSEM 2D	27.27%	15
OSEM 3D	61.82%	34
MLEM 2D	3.64%	2
MLEM 3D	16.36%	9
I don't know	16.36%	9
Total Respondents: 55		

Q51 Are you working with/using preclinical µCT Device(s)?

Answered: 130 Skipped: 21

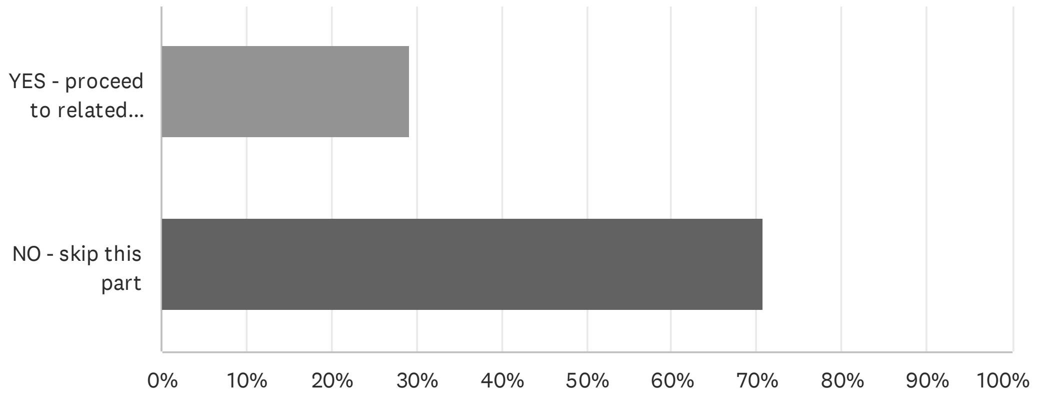

ANSWER CHOICES	RESPONSES	
YES - proceed to related questions	29.23%	38
NO - skip this part	70.77%	92
Total Respondents: 130		

Q52 Which µCT are you working with?

Answered: 32 Skipped: 119

#	RESPONSES	DATE
1	MILabs Vector+	1/27/2022 11:08 AM
2	Quantum GX 2	1/27/2022 2:13 AM
3	Bruker	1/26/2022 11:47 AM
4	MILabs Vector	1/26/2022 9:55 AM
5	Molecubes	1/24/2022 2:36 PM
6	skyscan1176, quantum gxII	1/21/2022 2:28 PM
7	inveon	1/21/2022 1:45 PM
8	MIlabs	1/21/2022 1:36 PM
9	Bruker	1/18/2022 11:25 AM
10	eXplore CT120, GE	1/17/2022 12:27 PM
11	XCUBE, Molecubes	1/15/2022 10:00 PM
12	Inveon CT	1/5/2022 1:55 PM
13	X-cube	12/21/2021 12:19 PM
14	INVEON	12/16/2021 11:36 AM
15	hybrid NanoScanPET/CT camera and microSPECT/CT+	12/15/2021 3:19 PM
16	SkyScan 1276	12/15/2021 10:49 AM
17	Siemens Inveon	12/14/2021 4:02 PM
18	IVIS CT	12/13/2021 1:28 PM
19	uCT of MILAbs in VECTor5	12/13/2021 11:55 AM
20	Siemens Inveon MM-CT, TriFoil Imaging eXplore CT120	12/13/2021 10:36 AM
21	X-Cube	12/13/2021 8:48 AM
22	nanoScan PET-CT (Mediso)	12/10/2021 4:52 PM
23	SARRP	12/10/2021 3:18 PM
24	skyscan1278, 1076, x-cube	12/10/2021 2:33 PM
25	QuantumFX, Skyscan 1272, Synchrotron microCT	12/10/2021 1:58 PM
26	Mediso PET-CT	12/10/2021 1:55 PM
27	Siemens Inveon	12/10/2021 1:50 PM
28	Molecubes X-cube	12/10/2021 1:48 PM
29	molecubes x-cube	12/10/2021 1:38 PM
30	MILabs' µCT	12/10/2021 1:37 PM
31	Inveon microCT	12/10/2021 1:17 PM
32	Molecubes	12/10/2021 1:01 PM

Q53 What is the frequency of regular scanner-maintenance by the manufacturer?'

Answered: 34 Skipped: 117

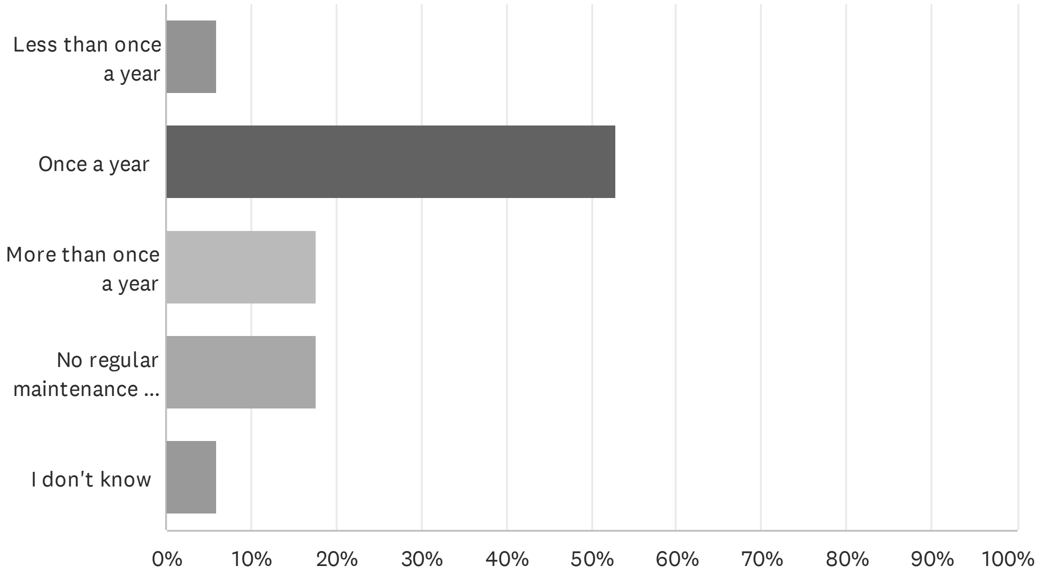

ANSWER CHOICES	RESPONSES	
Less than once a year	5.88%	2
Once a year	52.94%	18
More than once a year	17.65%	6
No regular maintenance by the manfacturer	17.65%	6
I don't know	5.88%	2
TOTAL		34

Q54 How ofter is the scanner calibrated?

Answered: 35 Skipped: 116

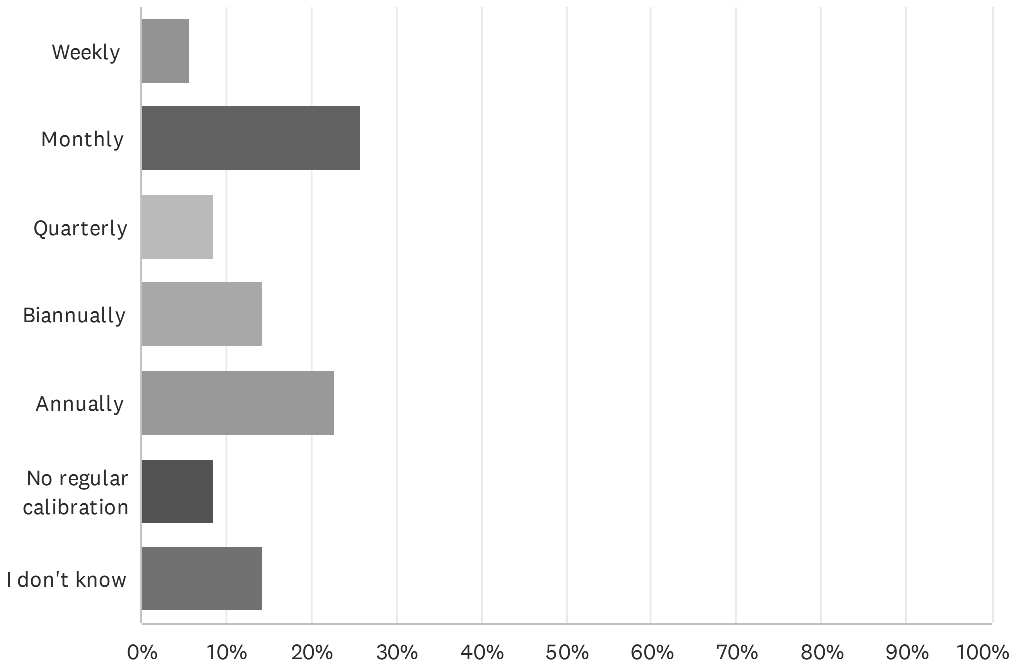

ANSWER CHOICES	RESPONSES	
Weekly	5.71%	2
Monthly	25.71%	9
Quarterly	8.57%	3
Biannually	14.29%	5
Annually	22.86%	8
No regular calibration	8.57%	3
I don't know	14.29%	5
TOTAL		35

Q55 What phantom(s) do you use for calibration or quality control?

Answered: 35 Skipped: 116

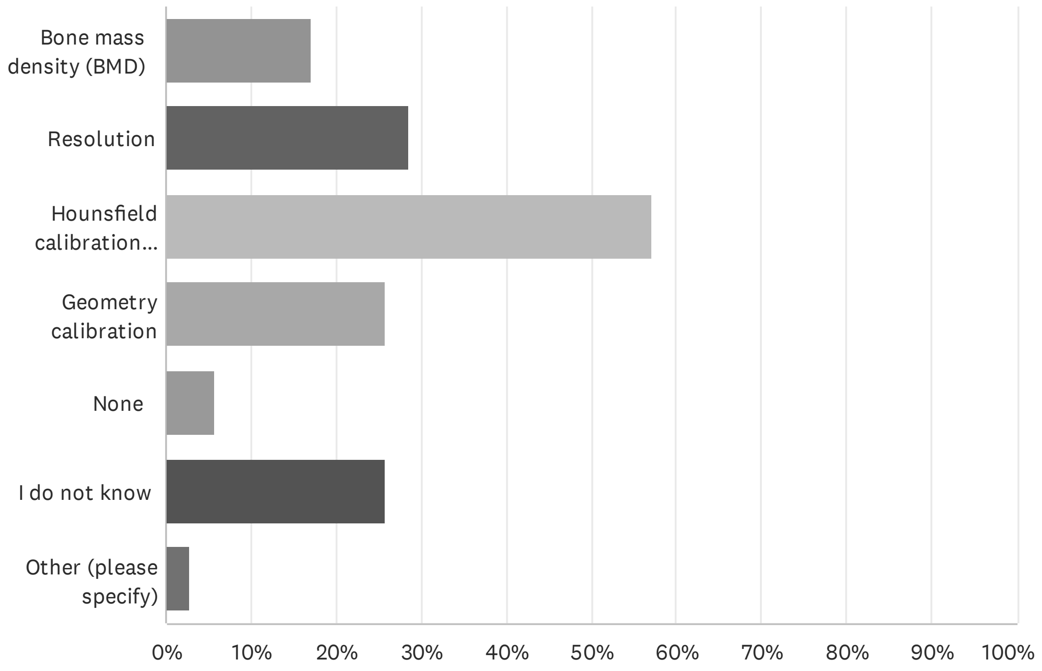

ANSWER CHOICES	RESPONSES	
Bone mass density (BMD)	17.14%	6
Resolution	28.57%	10
Hounsfield calibration (water)	57.14%	20
Geometry calibration	25.71%	9
None	5.71%	2
I do not know	25.71%	9
Other (please specify)	2.86%	1
Total Respondents: 35		

#	OTHER (PLEASE SPECIFY)	DATE
1	Co-registration with bcube	12/21/2021 12:19 PM

Q56 In your opinion - which parameters should be included in the description of experiments to ensure reproducibility.(tick all relevant items)

Answered: 33 Skipped: 118

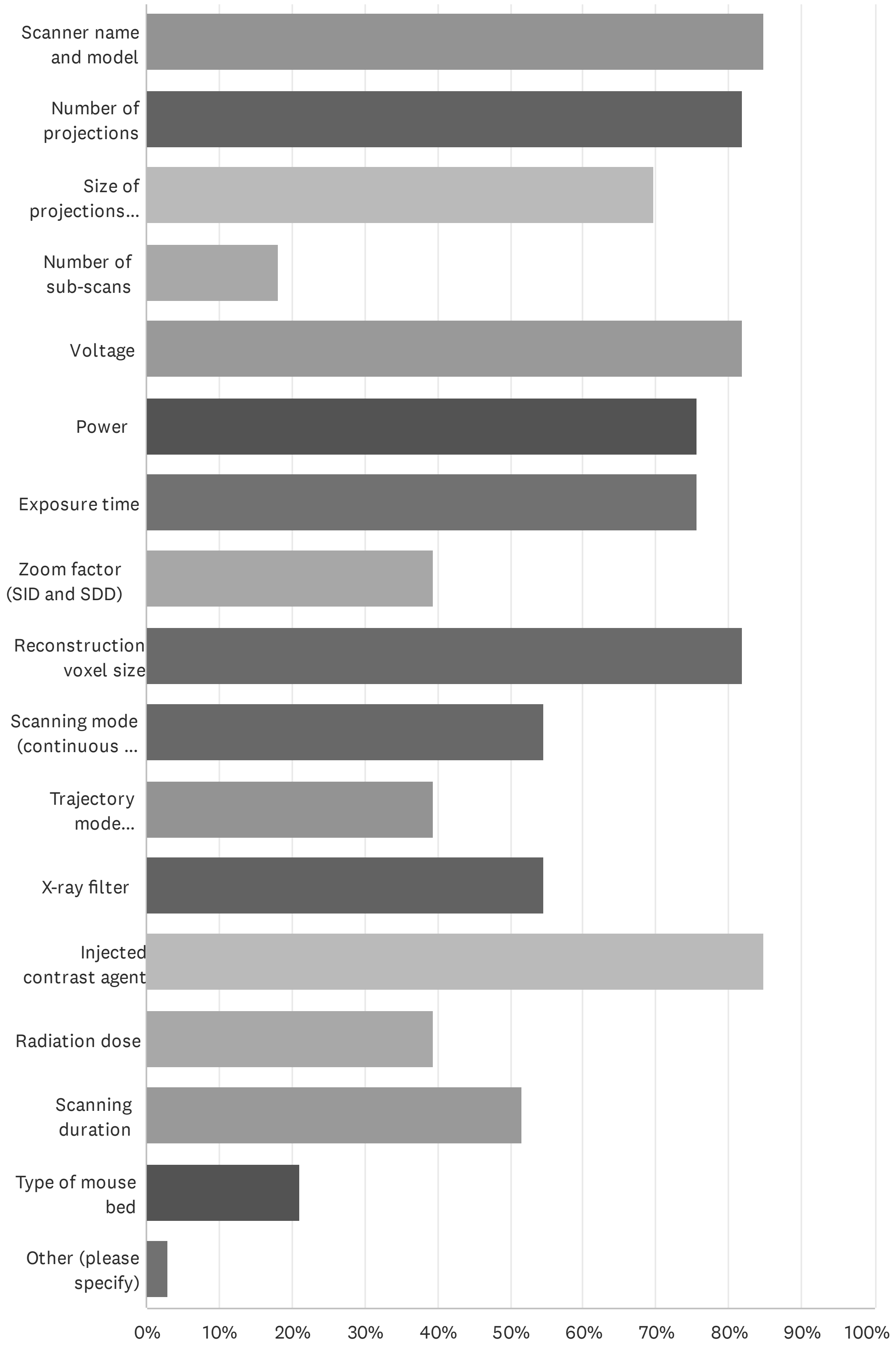

ANSWER CHOICES	RESPONSES	
Scanner name and model	84.85%	28
Number of projections	81.82%	27
Size of projections (and binning mode)	69.70%	23
Number of sub-scans	18.18%	6
Voltage	81.82%	27
Power	75.76%	25
Exposure time	75.76%	25
Zoom factor (SID and SDD)	39.39%	13
Reconstruction voxel size	81.82%	27
Scanning mode (continuous or step&shoot)	54.55%	18
Trajectory mode (circular/helical)	39.39%	13
X-ray filter	54.55%	18
Injected contrast agent	84.85%	28
Radiation dose	39.39%	13
Scanning duration	51.52%	17
Type of mouse bed	21.21%	7
Other (please specify)	3.03%	1
Total Respondents: 33		

#	OTHER (PLEASE SPECIFY)	DATE
1	all relevant - also including reconstruction parameters	12/10/2021 2:33 PM

Q57 Does your reconstruction software provide these features?

Answered: 35 Skipped: 116

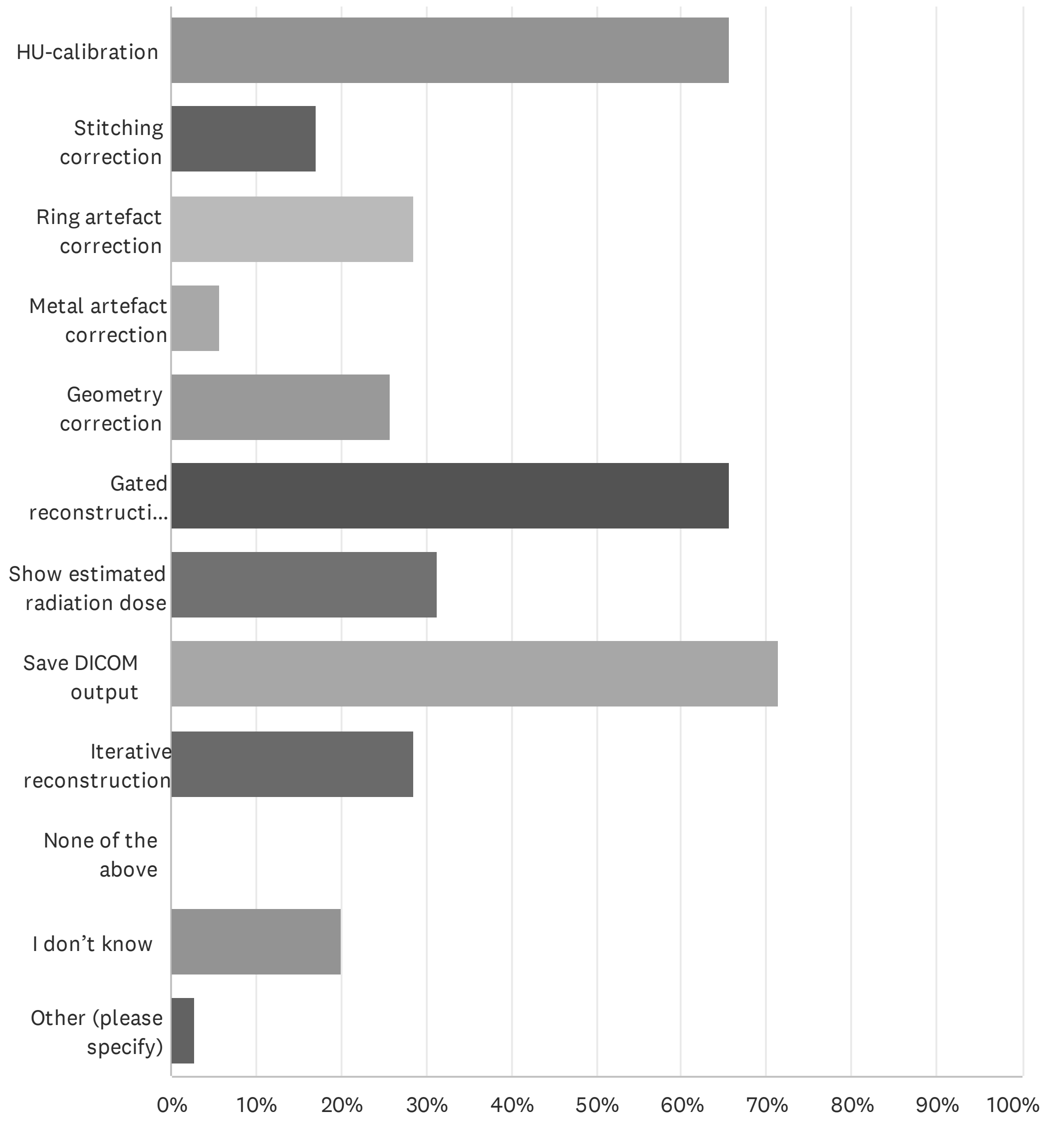

ANSWER CHOICES	RESPONSES	
HU-calibration	65.71%	23
Stitching correction	17.14%	6
Ring artefact correction	28.57%	10
Metal artefact correction	5.71%	2
Geometry correction	25.71%	9
Gated reconstruction (breathing/cardiac)	65.71%	23
Show estimated radiation dose	31.43%	11
Save DICOM output	71.43%	25
Iterative reconstruction	28.57%	10
None of the above	0.00%	0
I don’t know	20.00%	7
Other (please specify)	2.86%	1
Total Respondents: 35		

#	OTHER (PLEASE SPECIFY)	DATE
1	We used it mainly for anatomy	12/21/2021 12:19 PM

Q58 Are you working with/using preclinical MRI Device(s)?

Answered: 128 Skipped: 23

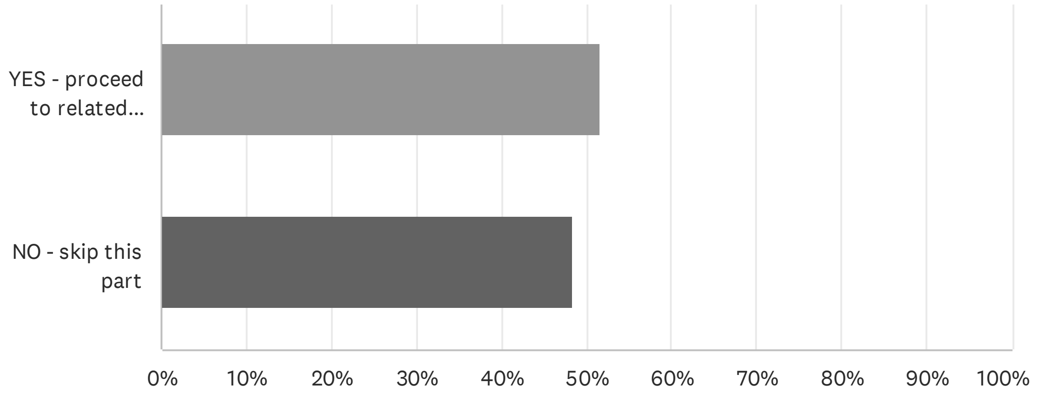

ANSWER CHOICES	RESPONSES	
YES - proceed to related questions	51.56%	66
NO - skip this part	48.44%	62
Total Respondents: 128		

Q59 Specify your scanner hardware (e.g. Bruker 9.4T with cryoprobe)

Answered: 57 Skipped: 94

#	RESPONSES	DATE
1	Bruker 9.4T with cryoprobe	1/26/2022 4:43 PM
2	Bruker 9.4T	1/25/2022 9:30 AM
3	Bruker 7 Teslas PT000111 & Bruker 11 Teslas PT000207	1/24/2022 2:40 PM
4	Bruker 7T, 13C cryocoil, RAPID and Bruker volume and surface coils	1/24/2022 2:19 PM
5	Bruker 7T with micro-imaging probe	1/24/2022 11:41 AM
6	Bruker Biospec 70/30, Bruker Clinscan 7T	1/21/2022 3:09 PM
7	Bruker	1/21/2022 2:21 PM
8	Bruker 11.7T & Bruker 7T	1/21/2022 2:08 PM
9	Bruker 9.4T with cryoprobe	1/21/2022 1:56 PM
10	bruker 9.4 t	1/21/2022 1:47 PM
11	Bruker 11T & Bruker 7T	1/21/2022 1:45 PM
12	Bruker 7T	1/21/2022 1:37 PM
13	Bruker	1/21/2022 1:34 PM
14	Bruker	1/18/2022 11:27 AM
15	Bruker BioSpec 4.7T & 11.7T / Bruker WB 9.4T	1/17/2022 12:29 PM
16	Bruder 9.4T, Bruket 1T	1/5/2022 1:58 PM
17	Bruker 7T	12/16/2021 12:30 PM
18	BRUKER 7T, avance I	12/16/2021 11:59 AM
19	Bruker 11T	12/16/2021 8:35 AM
20	7.0 T Bruker with 1H cryoprobe	12/15/2021 10:49 AM
21	Bruker BioSpec	12/14/2021 4:03 PM
22	Bruker Pharmascan 7T	12/14/2021 1:33 PM
23	Bruker 7T	12/13/2021 6:50 PM
24	Bruker 94/20 and 70/30	12/13/2021 3:45 PM
25	Bruker 7T	12/13/2021 2:38 PM
26	Bruker 9.4T with cryoprobe	12/13/2021 1:30 PM
27	Bruker 9.4T with cryoprobe, 7T, MRS 4.7T	12/13/2021 12:30 PM
28	Bruker 7 T	12/13/2021 11:52 AM
29	Varian/Agilent 9.4 T & Bruker 7T magnet with Philips console	12/13/2021 11:02 AM
30	Agilent 9.4T, Magritek Spinsolve Ultra, GE 750 3T	12/13/2021 8:55 AM
31	Bruker 7T PharmaScan 70/16	12/13/2021 8:49 AM
32	Bruker 7T Biospec	12/13/2021 6:51 AM
33	Bruker 1T (Icon 1T)	12/12/2021 2:12 AM
34	Bruker 11.7 T / Bruker 7 T	12/11/2021 2:21 PM
35	Agilent 7T interfaced to a bruker advance III console	12/11/2021 8:23 AM

36	Bruker 7T	12/11/2021 6:14 AM
37	Bruker 7T	12/11/2021 3:16 AM
38	BRUKER 9.4T	12/10/2021 8:37 PM
39	nanoScan Mediso 1T (2011)	12/10/2021 7:25 PM
40	Agilent Magnet with Bruker console (Paravision 7)	12/10/2021 5:52 PM
41	Bruker Biospec systems	12/10/2021 4:29 PM
42	Bruker 7T spectrometer and 9.4T animal scanner, Siemes scanners	12/10/2021 4:26 PM
43	Agilent 7T with varian console	12/10/2021 3:48 PM
44	Varian/Agilent, Siemens	12/10/2021 3:30 PM
45	Bruker 9.4T	12/10/2021 3:21 PM
46	Bruker Biospec 3T	12/10/2021 3:08 PM
47	Bruker 7 T	12/10/2021 2:51 PM
48	bruker 7t, 9.4T	12/10/2021 2:34 PM
49	Bruker 7T with cryoprobe	12/10/2021 2:29 PM
50	Bruker 7T	12/10/2021 2:16 PM
51	Bruker 7T, 4.7T	12/10/2021 2:04 PM
52	MR Solutions 7T MRI	12/10/2021 1:56 PM
53	Bruker PHS70/16	12/10/2021 1:52 PM
54	Mediso NanoScanPET/MRI 1T	12/10/2021 1:51 PM
55	Bruker 4.7	12/10/2021 1:50 PM
56	Bruker 11.7T , Clinscan 7T	12/10/2021 1:46 PM
57	Bruker 9.4T with cryoprobe	12/10/2021 1:44 PM

Q60 Do you follow Quality Assurance (QA) procedures?(e.g. AAPM 100 protocol on "Acceptance Testing and Quality Assurance Procedures for Magnetic Resonance Imaging Facilities www.aapm.org/pubs/reports/rpt_100.pdf)?

Answered: 63 Skipped: 88

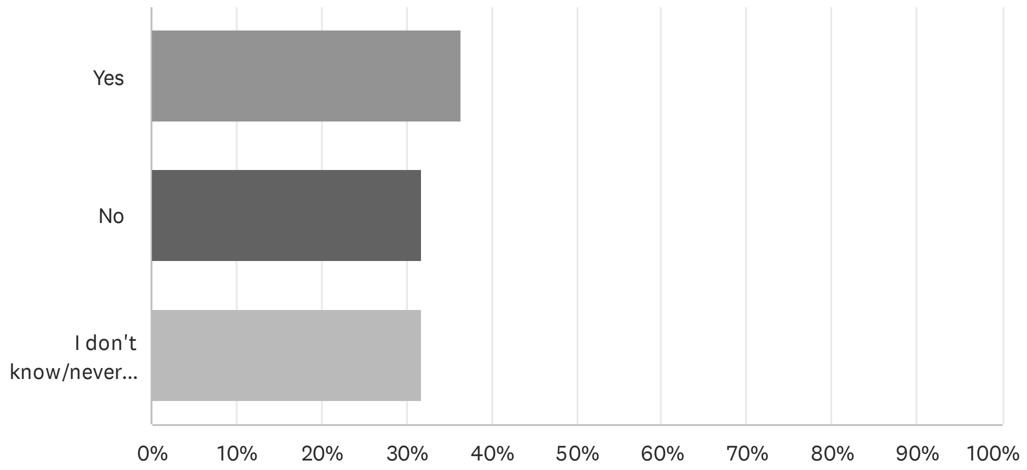

ANSWER CHOICES	RESPONSES	
Yes	36.51%	23
No	31.75%	20
I don't know/never heard of it.	31.75%	20
TOTAL		63

Q61 Which scans do you perform?

Answered: 60 Skipped: 91

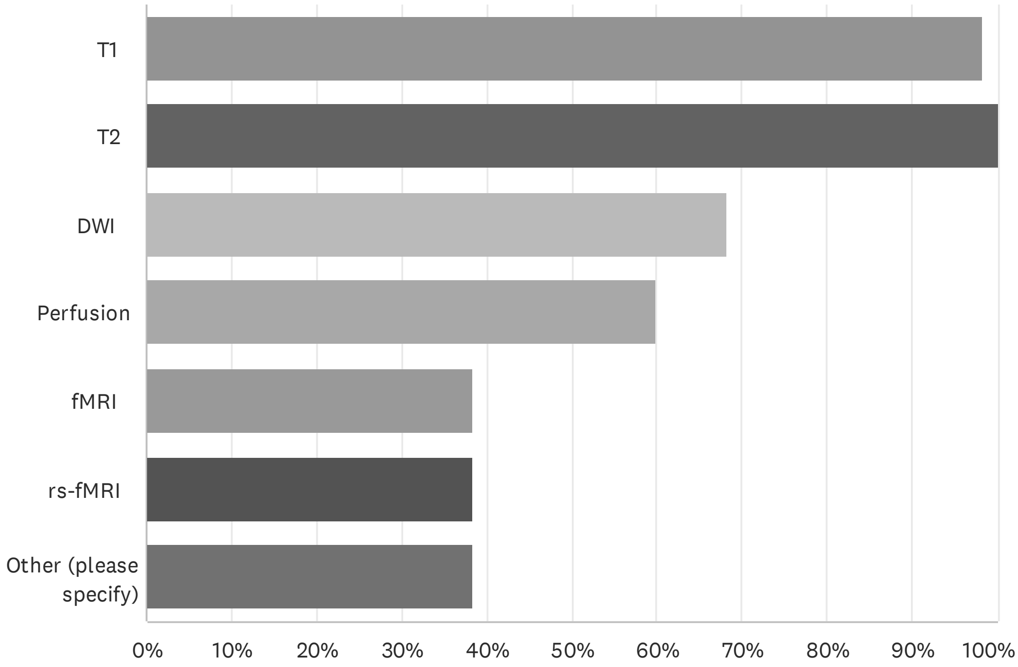

ANSWER CHOICES	RESPONSES	
T1	98.33%	59
T2	100.00%	60
DWI	68.33%	41
Perfusion	60.00%	36
fMRI	38.33%	23
rs-fMRI	38.33%	23
Other (please specify)	38.33%	23
Total Respondents: 60		

#	OTHER (PLEASE SPECIFY)	DATE
1	relaxometry, CSI, bSSFP, non-imaging spectroscopy	1/24/2022 2:19 PM
2	MRS	1/21/2022 2:08 PM
3	cardiac cine, angiography, spectroscopy, CEST	1/21/2022 1:56 PM
4	bold, told, vam, cest , specrtoscopy	1/21/2022 1:47 PM
5	UTE (2D, 3D), cardiac MRI, EPI,	1/21/2022 1:45 PM
6	CEST MRI	1/21/2022 1:34 PM
7	MRS, 19F MRI, 13C MRS, 31P MRS, CEST, MRA, T2*, CMRI, ...	1/18/2022 11:27 AM
8	CEST, SWI, MRS	12/22/2021 8:58 AM
9	DTI, 1H-spectroscopy, 19F-MRI	12/13/2021 6:50 PM
10	QSM, IVIM, MRS, T1rho, DENSE,	12/13/2021 3:45 PM

11	angiography	12/13/2021 11:02 AM
12	13C, 23Na, 31P, parametric imaging, CEST, spectroscopy	12/13/2021 8:55 AM
13	SWI, CEST, MTR	12/11/2021 2:21 PM
14	CEST, ASL, DCEMRI	12/11/2021 8:23 AM
15	DCE	12/10/2021 5:52 PM
16	19F imaging	12/10/2021 4:26 PM
17	SNR checks	12/10/2021 3:48 PM
18	MRS, Cine	12/10/2021 3:30 PM
19	DTI	12/10/2021 2:51 PM
20	Hyperpolarized MRI	12/10/2021 2:16 PM
21	CEST	12/10/2021 2:04 PM
22	CEST	12/10/2021 1:52 PM
23	CEST	12/10/2021 1:44 PM

Q62 What is the frequency of regular scanner-maintenance by the manufacturer?'

Answered: 62 Skipped: 89

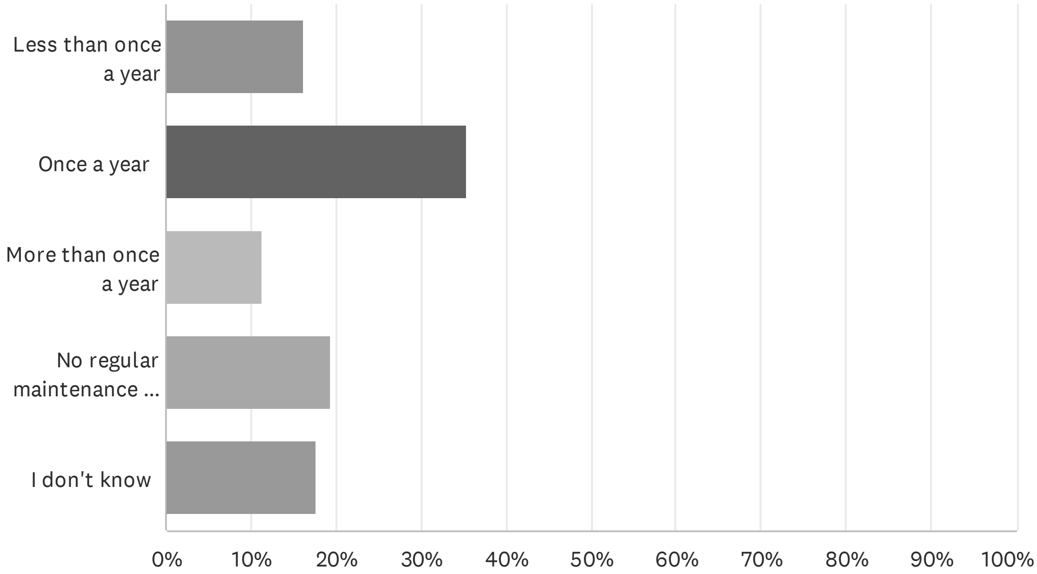

ANSWER CHOICES	RESPONSES	
Less than once a year	16.13%	10
Once a year	35.48%	22
More than once a year	11.29%	7
No regular maintenance by the manfacturer	19.35%	12
I don't know	17.74%	11
TOTAL		62

Q63 How ofter is the scanner calibrated?

Answered: 63 Skipped: 88

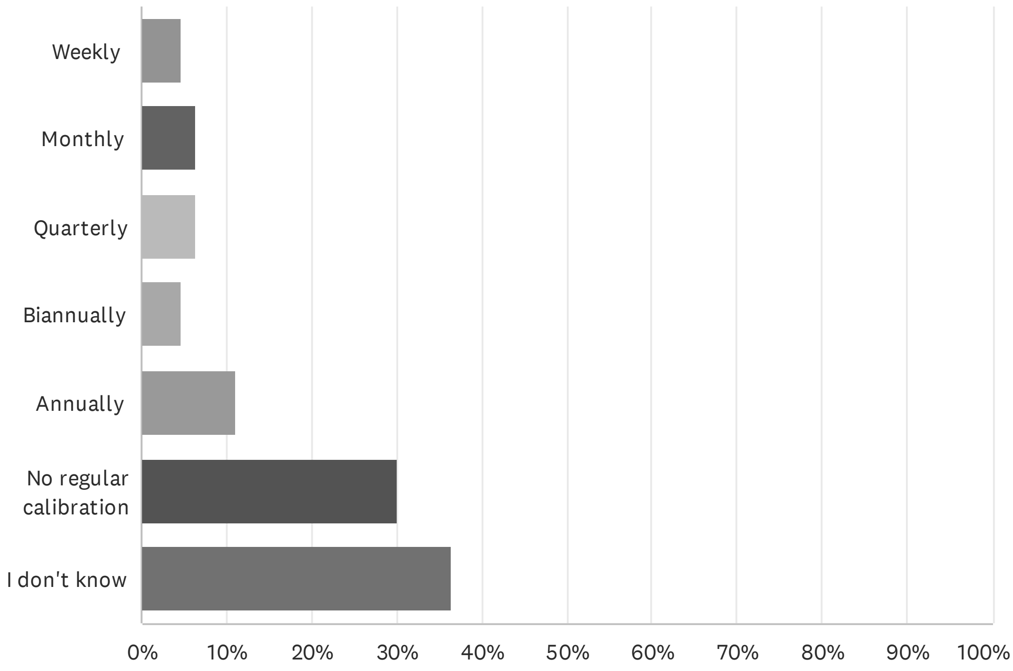

ANSWER CHOICES	RESPONSES	
Weekly	4.76%	3
Monthly	6.35%	4
Quarterly	6.35%	4
Biannually	4.76%	3
Annually	11.11%	7
No regular calibration	30.16%	19
I don't know	36.51%	23
TOTAL		63

Q64 What phantom(s) do you use for calibration or quality control?(if none are used, leave question unanswered)

Answered: 33 Skipped: 118

#	RESPONSES	DATE
1	Manufacturer supplied phantoms, custom-made tube and vials of 13C compounds	1/24/2022 2:19 PM
2	Home-made phantom	1/24/2022 11:41 AM
3	1H-water and x-nuclei phantoms	1/21/2022 3:09 PM
4	Manufacturer's quality control phantoms	1/21/2022 2:08 PM
5	Manufacturer provides aqueous solutions to mimic tissue properties.	1/21/2022 1:56 PM
6	mri phantoms	1/21/2022 1:47 PM
7	T1, T2 fantoms	1/21/2022 1:45 PM
8	My home-built phantoms	1/21/2022 1:34 PM
9	standard Bruker phantoms	1/18/2022 11:27 AM
10	'Rat head phantom' (i.e., doped salted water) + home-made lego phantom	1/17/2022 12:29 PM
11	Lego	12/22/2021 8:58 AM
12	NiSO4, CuSO4, Agar, Gd-dopped, NaCl	12/16/2021 11:59 AM
13	manufacturer provided	12/16/2021 8:35 AM
14	water	12/15/2021 10:49 AM
15	Vendor's phantoms	12/13/2021 6:50 PM
16	resolution phantom, and CuSO4 phantom for SNR	12/13/2021 3:45 PM
17	Phantoms with defined geometry	12/13/2021 1:30 PM
18	I don't know	12/13/2021 12:30 PM
19	home-made spherical phantoms or tube phantoms	12/13/2021 11:02 AM
20	in-house phantoms, manufacture phantoms, CaliberMRI phantoms	12/13/2021 8:55 AM
21	Water phantom	12/13/2021 8:49 AM
22	None	12/11/2021 8:23 AM
23	A number of home built phantoms	12/10/2021 8:37 PM
24	done by Mediso	12/10/2021 7:25 PM
25	Sphere phantom	12/10/2021 3:48 PM
26	water	12/10/2021 3:21 PM
27	up to the manifacturer, they do the annual maintenance once a year and they use their phantoms	12/10/2021 3:08 PM
28	Water, AGAR, DWI phantoms	12/10/2021 2:51 PM
29	Home-Built phantoms for different purposes	12/10/2021 2:16 PM
30	Own	12/10/2021 2:13 PM
31	my own phantoms	12/10/2021 2:04 PM
32	T1-Phantom, Brain-Phantom, Geometry-Phantom	12/10/2021 1:52 PM
33	nickel chloride	12/10/2021 1:51 PM

Q65 Does regular Quality Control include testing of:(tick all relevant items)

Answered: 60 Skipped: 91

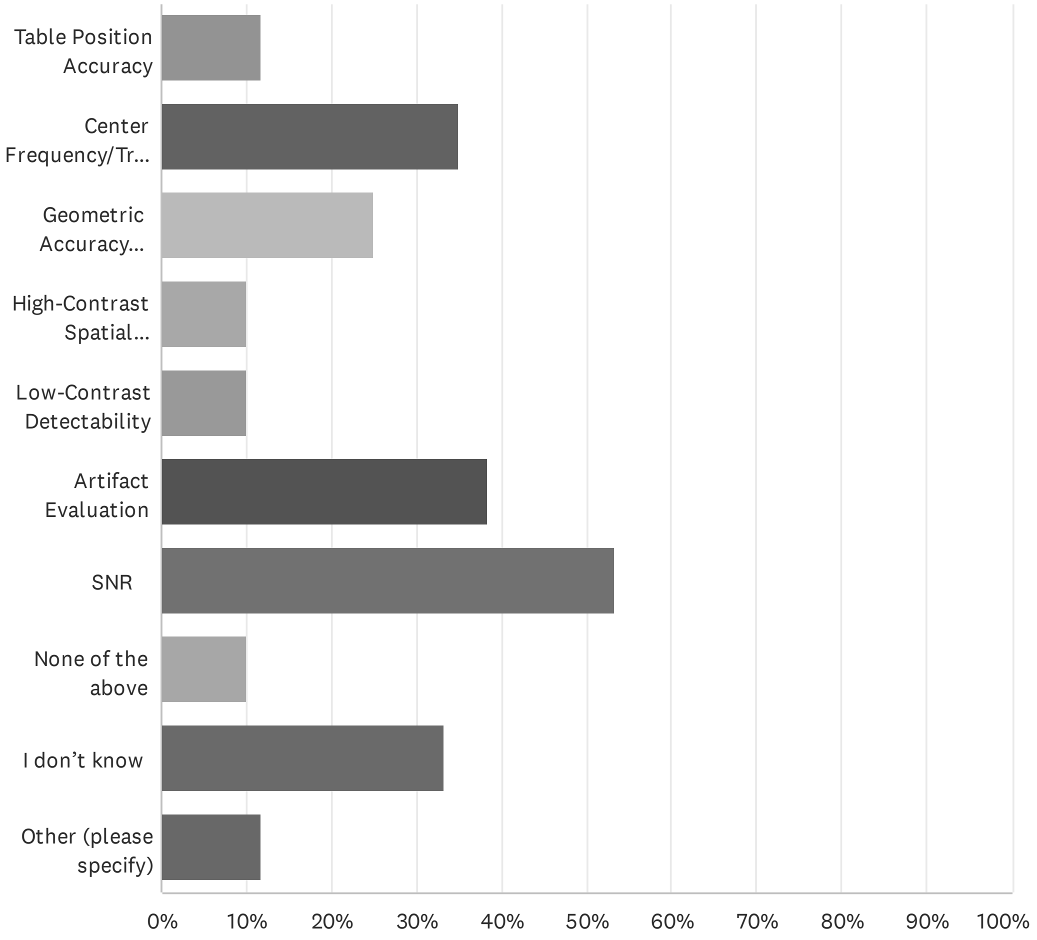

ANSWER CHOICES	RESPONSES	
Table Position Accuracy	11.67%	7
Center Frequency/Transmitter Gain or Attenuation	35.00%	21
Geometric Accuracy Measurements	25.00%	15
High-Contrast Spatial Resolution	10.00%	6
Low-Contrast Detectability	10.00%	6
Artifact Evaluation	38.33%	23
SNR	53.33%	32
None of the above	10.00%	6
I don’t know	33.33%	20
Other (please specify)	11.67%	7
Total Respondents: 60		

#	OTHER (PLEASE SPECIFY)	DATE

1	coil B1 and image uniformity	1/24/2022 2:19 PM
2	B0, B1 maps	1/21/2022 1:34 PM
3	Gradient linearity	12/16/2021 11:59 AM
4	field homogeneity, B1 homogeneity for each transmit coil, water suppression performance,	12/13/2021 3:45 PM
5	Coil perfomance, gradient noise	12/13/2021 8:55 AM
6	(other member in our team responsible for this)	12/10/2021 2:34 PM
7	i check coil performance (SNR, axial FOV); B1 and B0; with and without my PET insert; I have a CEST phantom	12/10/2021 2:04 PM

Q66 In which country are you working?

Answered: 115 Skipped: 36

ANSWER CHOICES	RESPONSES	
Name	0.00%	0
Company	0.00%	0
Address	0.00%	0
Address 2	0.00%	0
City/Town	0.00%	0
State/Province	0.00%	0
ZIP/Postal Code	0.00%	0
Country	100.00%	115
Email Address	0.00%	0
Phone Number	0.00%	0

#	NAME	DATE
	There are no responses.	

#	COMPANY	DATE
	There are no responses.	

#	ADDRESS	DATE
	There are no responses.	

#	ADDRESS 2	DATE
	There are no responses.	

#	CITY/TOWN	DATE
	There are no responses.	

#	STATE/PROVINCE	DATE
	There are no responses.	

#	ZIP/POSTAL CODE	DATE
	There are no responses.	

#	COUNTRY	DATE
1	Belgium	1/27/2022 11:09 AM
2	United States	1/27/2022 2:14 AM
3	Belgium	1/26/2022 4:44 PM
4	Spain	1/26/2022 11:48 AM
5	Germany	1/26/2022 9:56 AM
6	Netherlands	1/26/2022 9:19 AM
7	Austria	1/25/2022 9:31 AM
8	Netherlands	1/24/2022 8:20 PM
9	Spain	1/24/2022 2:40 PM

10	Germany	1/24/2022 2:20 PM
11	Italy	1/24/2022 11:42 AM
12	Germany	1/24/2022 9:03 AM
13	Belgium	1/24/2022 8:28 AM
14	United Kingdom	1/22/2022 10:23 AM
15	Germany	1/21/2022 3:14 PM
16	UK	1/21/2022 2:29 PM
17	Germany	1/21/2022 2:21 PM
18	Spain	1/21/2022 2:09 PM
19	Germany	1/21/2022 1:56 PM
20	austria	1/21/2022 1:47 PM
21	Spain	1/21/2022 1:46 PM
22	Germany	1/21/2022 1:37 PM
23	USA	1/21/2022 1:35 PM
24	Belgium	1/18/2022 11:28 AM
25	France	1/17/2022 12:30 PM
26	Usa	1/15/2022 10:01 PM
27	United States	1/10/2022 6:21 PM
28	Belgium	1/5/2022 5:21 PM
29	Austria	1/5/2022 1:59 PM
30	DE	12/22/2021 8:59 AM
31	Italy	12/21/2021 12:19 PM
32	Ukraine	12/21/2021 12:04 AM
33	UK	12/16/2021 5:33 PM
34	Croatia	12/16/2021 12:31 PM
35	France	12/16/2021 12:00 PM
36	spain	12/16/2021 8:36 AM
37	France	12/15/2021 3:22 PM
38	Germany	12/15/2021 10:49 AM
39	Germany	12/14/2021 4:03 PM
40	Italy	12/14/2021 1:34 PM
41	UK	12/14/2021 12:05 PM
42	Belgium	12/14/2021 9:26 AM
43	Italy	12/13/2021 6:51 PM
44	UK	12/13/2021 4:54 PM
45	The Netherlands	12/13/2021 3:56 PM
46	Netherlands	12/13/2021 3:53 PM
47	Belgium	12/13/2021 3:45 PM
48	Germany	12/13/2021 2:39 PM
49	Germany	12/13/2021 1:30 PM
50	Belgium	12/13/2021 12:32 PM

51	Netherlands	12/13/2021 11:56 AM
52	Italy	12/13/2021 11:52 AM
53	Netherlands	12/13/2021 11:03 AM
54	Spain	12/13/2021 11:01 AM
55	Germany	12/13/2021 10:37 AM
56	Netherlands	12/13/2021 10:21 AM
57	Germany	12/13/2021 9:24 AM
58	Denmark	12/13/2021 8:56 AM
59	Belgium	12/13/2021 8:50 AM
60	UK	12/13/2021 6:52 AM
61	Germany	12/12/2021 5:43 PM
62	Japan	12/12/2021 2:18 AM
63	United Kingdom	12/12/2021 1:59 AM
64	Greece	12/11/2021 6:07 PM
65	Spain	12/11/2021 2:22 PM
66	UK	12/11/2021 8:23 AM
67	Germany	12/11/2021 6:15 AM
68	Germany	12/11/2021 3:16 AM
69	Israel	12/10/2021 8:37 PM
70	Germany	12/10/2021 7:25 PM
71	Czechia	12/10/2021 7:09 PM
72	USA	12/10/2021 6:51 PM
73	United Kingdom	12/10/2021 6:06 PM
74	France	12/10/2021 4:52 PM
75	Germany	12/10/2021 4:48 PM
76	Germany	12/10/2021 4:30 PM
77	Germany	12/10/2021 4:27 PM
78	United States	12/10/2021 4:07 PM
79	netherlands	12/10/2021 4:01 PM
80	UK	12/10/2021 3:49 PM
81	Netherlands	12/10/2021 3:44 PM
82	Switzerland	12/10/2021 3:30 PM
83	Israel	12/10/2021 3:21 PM
84	Scotland	12/10/2021 3:18 PM
85	Italy	12/10/2021 3:15 PM
86	Germany	12/10/2021 3:09 PM
87	Rome	12/10/2021 3:02 PM
88	Germany	12/10/2021 2:51 PM
89	germany	12/10/2021 2:35 PM
90	België	12/10/2021 2:34 PM
91	Italy	12/10/2021 2:31 PM

92	Switzerland	12/10/2021 2:30 PM
93	spain	12/10/2021 2:19 PM
94	France	12/10/2021 2:18 PM
95	Germany	12/10/2021 2:16 PM
96	France	12/10/2021 2:15 PM
97	USA	12/10/2021 2:05 PM
98	Netherlands	12/10/2021 2:04 PM
99	Germany	12/10/2021 1:59 PM
100	Norway	12/10/2021 1:57 PM
101	Polska	12/10/2021 1:55 PM
102	Germany	12/10/2021 1:53 PM
103	United Kingdom	12/10/2021 1:51 PM
104	France	12/10/2021 1:50 PM
105	Belgium	12/10/2021 1:49 PM
106	NL	12/10/2021 1:46 PM
107	Germany	12/10/2021 1:45 PM
108	UK	12/10/2021 1:42 PM
109	Switzerland	12/10/2021 1:42 PM
110	Norway	12/10/2021 1:40 PM
111	Germany	12/10/2021 1:39 PM
112	Germany	12/10/2021 1:38 PM
113	China	12/10/2021 1:36 PM
114	Belgium	12/10/2021 1:17 PM
115	Belgium	12/10/2021 1:01 PM

#	EMAIL ADDRESS	DATE
	There are no responses.	

#	PHONE NUMBER	DATE
	There are no responses.	

Q67 I am a

Answered: 114 Skipped: 37

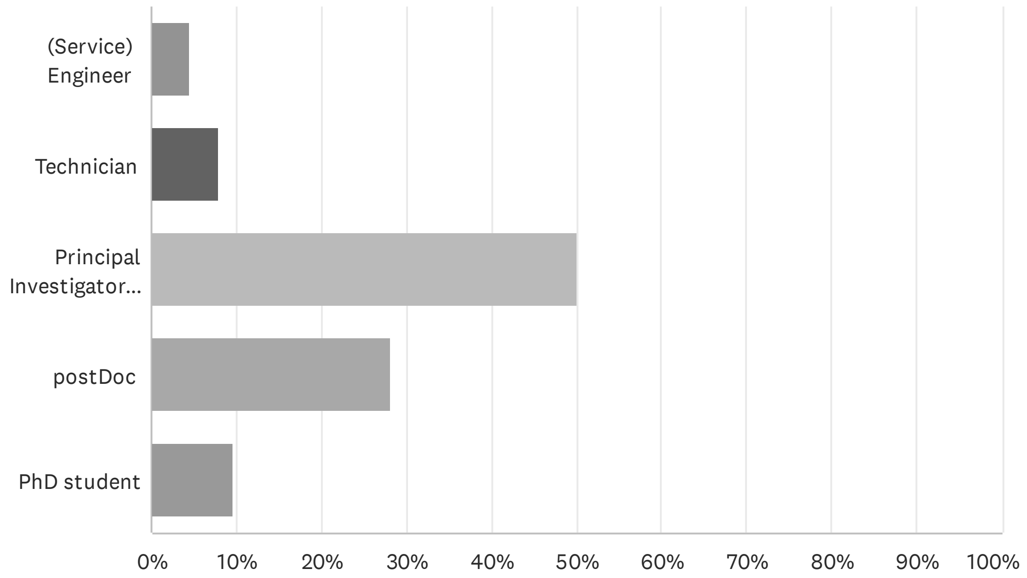

ANSWER CHOICES	RESPONSES	
(Service) Engineer	4.39%	5
Technician	7.89%	9
Principal Investigator/Group Leader	50.00%	57
postDoc	28.07%	32
PhD student	9.65%	11
TOTAL		114
